# Bridge Cross-Coupling of Bicyclo[1.1.0]butanes

**DOI:** 10.1021/acs.orglett.3c04030

**Published:** 2023-12-29

**Authors:** Ryan E. McNamee, Ayan Dasgupta, Kirsten E. Christensen, Edward A. Anderson

**Affiliations:** Chemistry Research Laboratory, Department of Chemistry, University of Oxford, 12 Mansfield Road, Oxford OX1 3TA, U.K.

## Abstract

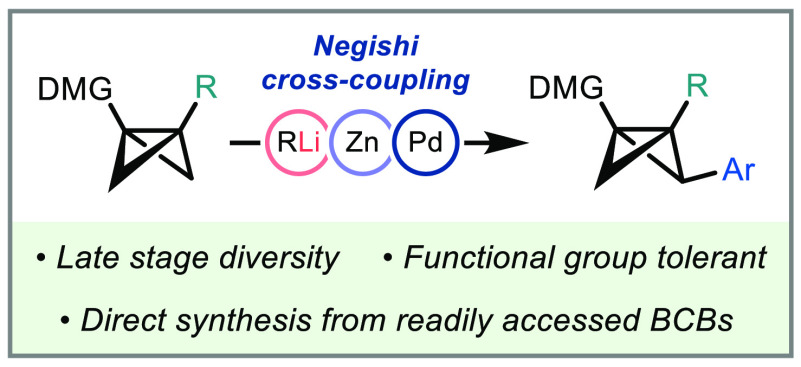

Bicyclo[1.1.0]butanes (BCBs) have gained growing popularity in “strain release” chemistry for the synthesis of four-membered-ring systems and *para*- and *meta*-disubstituted arene bioisosteres as well as applications in chemoselective bioconjugation. However, functionalization of the bridge position of BCBs can be challenging due to the inherent strain of the ring system and reactivity of the central C–C bond. Here we report the first late-stage bridge cross-coupling of BCBs, mediated by directed metalation/palladium catalysis.

Bicyclo[1.1.0]butanes (BCBs) (Figure 1a) are a class of highly strained hydrocarbons that have become valuable tools for “strain release” chemistry.^1^ These reagents possess the impressive ability to react with nucleophiles,^2^ radicals,^3^ electrophiles,^4^ and transition metal catalysts,^5^ with applications ranging from the synthesis of natural products^2d^ and *para-*(6) and *meta-*substituted^7^ arene bioisosteres to use as cystine-selective bioconjugation agents.^2b,8^ Access to these building blocks has been streamlined with the recent developments of late-stage bridgehead and bridge metalation protocols that deliver a broad portfolio of BCBs,^9^ including a convenient one-pot sulfone-based reaction sequence that affords exceptional diversity.^10^

**Figure 1:**
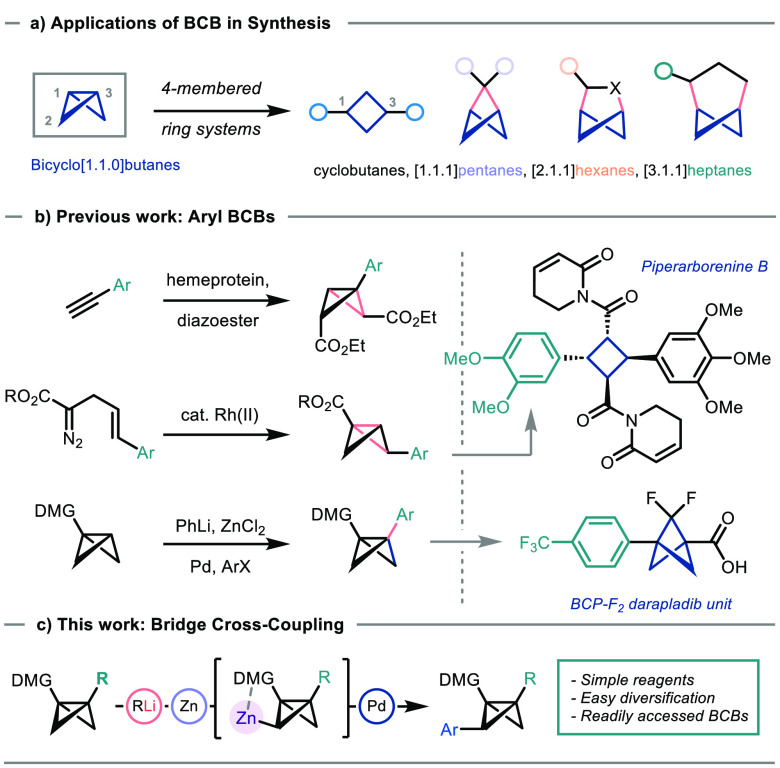
(a) Applications of BCBs in cyclobutane and bioisostere synthesis. (b) Previous work toward the construction of aryl-substituted BCBs. (c) Bridge directed metalation and cross-coupling of BCBs. DMG = directing metalation group.

BCBs that are aryl-substituted at the bridge carbon atoms are attractive targets due to their potential use in accessing arene-functionalized products upon ring opening. Specifically, access to these products would open up new avenues for medicinal chemists in bicyclo[1.1.1]pentane, -[2.1.1]hexane, and -[3.1.1]heptane synthesis for reaching novel chemical space.^7b^ Recent methodologies developed for the synthesis of aryl-substituted BCBs include (1) biocatalytic double diazo–alkyne condensation that introduces two identical *endo*/*exo* bridge ester substituents (bridgehead aryl, Figure 1b);^11^ (2) asymmetric intramolecular diazo insertion into styrenes, catalyzed by rhodium(II) (bridge aryl);^2e,12^ and (3) bridgehead-directed metalation and cross-coupling (bridgehead aryl).^9a^ The methods outlined in each case address different challenges, such as the latter providing a divergent synthesis of bicyclopentylation reagents, and the asymmetric diazo insertion facilitating a route toward the total synthesis of piperarborenine B.^2d^ However, although intramolecular diazo insertion offers a powerful method for asymmetric bridge-arylated BCB synthesis, it suffers from the drawback of synthetic linearity rather than late-stage diversification.

We previously developed a method that enables late-stage bridge functionalization through directed metalation/electrophilic quench,^9b^ although this tactic did not enable the introduction of aryl and alkenyl substituents. We questioned whether we might be able to extend this approach to bridge cross-coupling by transmetalation of the intermediate organolithium, enabling the rapid delivery of bridge aryl-substituted strain release reagents (Figure 1c). Notably, a similar strategy has been employed in the elegant polyfunctionalization of cubanes.^13^

Reaction development began by employing three potential BCB organometallic coupling partners, boronic acid **1a**, stannane **1b**, and organozinc **1c** (prepared from metalation of BCB **2a** with organolithiums and electrophilic quench (**1a**/**b**) or transmetalation to ZnCl_2_ (**1c**)), in Suzuki, Stille, and Negishi coupling protocols, respectively (Table 1, entries 1–3). Interestingly, the former two strategies led only to complete decomposition of the starting material with no observable product, while entry 3 returned **2a** with no sign of degradation. This was surprising given previous reports on cyclopropylzinc Negishi couplings as well as our own work on BCB bridgehead Negishi reactivity,^9a,14^ and it was hypothesized that TMEDA might be interfering with the reaction. To our delight, the use of TMEDA-free metalation in the generation of **3a** (*t*-BuLi in THF) and submission to equivalent coupling conditions (Pd(dba)_2_/2PPh_3_) achieved cross-coupling in 28% yield (as determined by ^1^H NMR spectroscopy; entry 4). A screen of 13 phosphine ligands was conducted, with the Buchwald-based ligands producing the highest yields and CyJPhos being optimal (48%; entry 5).^15^ A temperature and solvent screen identified THF at 65 °C as being crucial for this transformation (entries 6 and 7). Increasing the equivalents of iodobenzene led to a further increase in the yield (60%; entry 8). While a useful result, the conversion could be further enhanced by increasing the catalyst loading to 15 mol %, giving **3a** in 71% yield (entry 9). On scale-up, it became apparent that stirring the reaction mixture for 1 h at room temperature was crucial; otherwise, the reaction would fail due to Pd black formation.

**Table 1:**
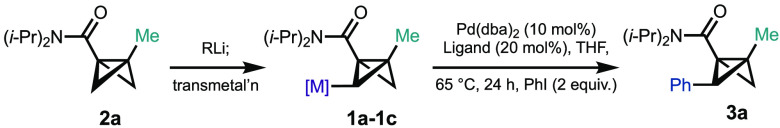
BCB Cross-Coupling Optimization^a^

entry	[M]	ligand, variation of conditions	yield (%)^b^
1	B(OH)_2_ (**1a**)	PPh_3_	0
2	Sn(*n*-Bu)_3_ (**1b**)	AsPh_3_	0
3	ZnCl·LiCl (**1c**)	PPh_3_, TMEDA (1.1 equiv)	0
4	ZnCl·LiCl (**1c**)	PPh_3_	28
5	ZnCl·LiCl (**1c**)	CyJPhos	48
6	ZnCl·LiCl (**1c**)	CyJPhos, 20 °C	6
7	ZnCl·LiCl (**1c**)	CyJPhos, DMF/Tol/1,4-dioxane^c^	–d
8	ZnCl·LiCl (**1c**)	CyJPhos, PhI (4.0 equiv)	60
9	ZnCl·LiCl (**1c**)	CyJPhos_2_ (30 mol %) and Pd(dba)_2_ (15 mol %)^e^	71

a Reactions were conducted on a 0.1 mmol scale. See the Supporting Information for details of metalation protocols in the synthesis of **1a**–**1c**.

b Determined by ^1^H NMR spectroscopic analysis of the crude reaction mixtures using 1,3,6-trimethoxybenzene as an internal standard.

c Heated at 110 °C.

d Extensive decomposition was observed in these solvents.

e Stirred for 1 h at room temperature before heating.

With optimized metalation and cross-coupling conditions in hand, we then examined the scope of the reaction (Scheme 1). A selection of aryl iodides bearing electron-withdrawing and -donating groups at the *para* position was first investigated. To our delight, these couplings proceeded in good to excellent yields (**3a**–**3e**, 60–84%). Reaction efficiency was maintained with *ortho*-substituted aryl iodide derivatives (**3f** and **3g**). The introduction of biorelevant functionality was also possible, for example, incorporating a galactose-bearing side chain in excellent yield (**3h**, 91%, 1:1 *dr* due to the racemic generation of **1c**).

**Scheme 1:**
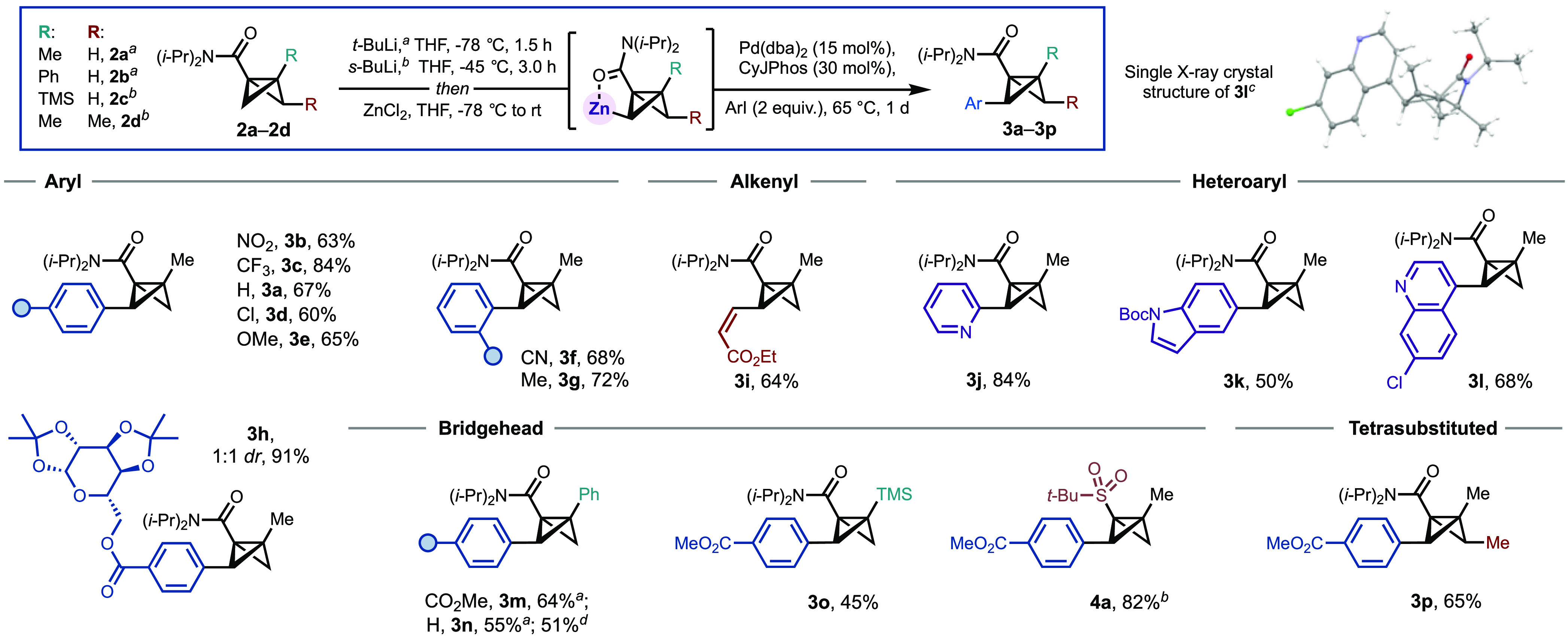
Synthesis of Aryl Bridge-Substituted BCBs Reaction conditions, unless stated otherwise: **2a** or **2b** (0.20 mmol, 1.0 equiv); 1. Lithiation: ^*a*^*t*-BuLi (0.22 mmol, 1.1 equiv, 1.1–1.3 M in pentane, THF, −78 °C, 1.5 h; ^*b*^*s*-BuLi (0.22 mmol, 1.1 equiv, 1.1–1.5 M in cyclohexane), THF, −45 °C, 3.0 h; 2. Transmetalation/coupling: ZnCl_2_ (0.22 mmol, 1.1 equiv), THF, −78 °C (or −45 °C) to rt; Pd(dba)_2_ (15 mol %), CyJPhos (30 mol %), R–I (0.4 mmol, 2.0 equiv) THF, 1 h, 20 °C; 1 d, 65 °C. ^*c*^Structure of **3l** from single-crystal X-ray diffraction studies (displacement ellipsoids drawn at 50% probability). ^*d*^The reaction was conducted on a 1 mmol scale.

Alkenyl iodides were also compatible with the coupling conditions (**3i**, 64%); however, alkenes bearing an electron-withdrawing group were essential for the product stability. Heterocycle cross-coupling is also highly appealing from a medicinal chemistry stance due to the application of BCBs in *para*- and *meta*-arene bioisostere synthesis. We were therefore delighted to find that a representative range of azacycles could be installed in good yields (50–84%), including 2-substituted pyridine (**3j**), indole (**3k**), and quinoline (**3i**). Pleasingly, these conditions could also be applied to BCB **2b**, which is more sterically demanding at the bridgehead position (Ph substituent), giving **3m** (64%) and **3n** (55%). The latter coupling was also carried out on a 1 mmol scale without significant detriment to the yield (51%).

Cross-coupling on other BCBs, such as trimethylsilyl-substituted BCB **2c** and trisubstituted BCB **2d** would demonstrate the feasibility of constructing more complex derivatives, including a tetrasubstituted product. However, no product was observed when **2c** and **2d** were subjected to the developed metalation and cross-coupling conditions, with neither undergoing productive metalation with *t*-BuLi at −78 °C in THF. Fortunately, TMEDA-free conditions for directed lithiation were identified (*s*-BuLi at −45 °C in THF)^15^ that could be applied to **2c** and **2d**. These substrates were then subjected to the cross-coupling conditions and, to our delight, produced silyl-substituted BCB **3o** in 45% yield and tetrasubstituted BCB **3p** in 65% yield. Resolving the cross-coupling issue of **2c** and **2d** inspired us to examine sulfone substrates; pleasingly, **4a** could be obtained in an excellent yield of 82% with the *s*-BuLi metalation conditions.

Having successfully demonstrated cross-coupling with trisubstituted BCB **2d**, we questioned whether a complementary approach could be established through a second directed bridge metalation *after* bridge arylation (Scheme 2). BCB **3c** was chosen as a candidate, as the bridge arene possesses a *para* electron-withdrawing group (CF_3_) that is tolerant of organolithiums. This substrate presents a regioselectivity challenge: the possibility of directed metalation (**5a**, Scheme 2) or benzylic deprotonation (**5b**), both of which would provide a useful class of novel BCBs. Surprisingly, when **3c** was subjected to the optimized conditions, neither the unsubstituted bridge nor the benzylic bridge underwent lithiation. Instead, *s*-BuLi was directed to the bridgehead methyl group, which then underwent BCB ring opening to give the corresponding enolate; quenching with allyl bromide afforded polysubstituted exocyclic cyclobutene **6**. This observation of this alternative metalation pathway may relate to restricted rotation of the directing group in substrate **3c**, which prevents access to the unsubstituted bridge, as observed in the X-ray crystal structure of **3l** and ^1^H NMR spectrum of the bridge aryl-BCB derivatives.^15^

**Scheme 2:**
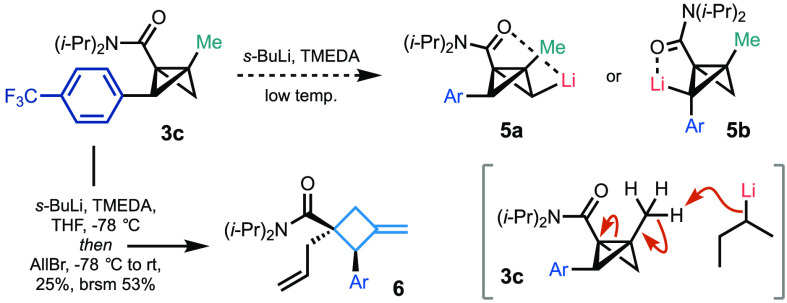
Unexpected Fragmentation in the Metalation of Bridge-Arylated BCB **3c** The reaction was run on a 0.12 mmol scale using optimized conditions with allyl bromide (0.6 mmol, 5.0 equiv) quench.

In summary, we have developed a convenient and general late-stage Negishi cross-coupling strategy to access sp^2^-bridge-substituted BCBs. This approach enables the introduction of arenes, heteroarenes, and alkenes with broad functional group tolerance with respect to the arene: nitro, ester, halide, silyl, nitrile, ether, and acetal groups are all accommodated, which can allow for further manipulation. This approach enables the rapid delivery of new strain release reagents, which we expect to be of use to the wider chemical community for small-ring and bioisostere construction.

## Data Availability

The data underlying this study are available in the published article and its Supporting Information.

## Supporting Information Available

Additional experimental discussion, experimental procedures, and copies of ^1^H and ^13^C NMR spectra (PDF)
