# Supplementary material for: Bridge Cross-Coupling of Bicyclo[1.1.0]butanes
Source: Org Lett. 2023 Dec 29;26(1):360–4. doi: 10.1021/acs.orglett.3c04030 (PMC10789093; doi:10.1021/acs.orglett.3c04030)
Supplement: Supplementary file 1 — ol3c04030_si_001.pdf [file ol3c04030_si_001.pdf]

Supporting Information

Bridge Cross-Coupling of Bicyclo[1.1.0]butanes

Ryan E. McNamee, Ayan Dasgupta, Kirsten E. Christensen,

and Edward A. Anderson*

Chemistry Research Laboratory, Department of Chemistry, University of Oxford, 12 Mansfield Road, Oxford, OX1 3TA, U.K.

Email: edward.anderson@chem.ox.ac.uk

Contents

1. Experimental procedures 2
   1.1 General comments 2
   1.2 Compounds prepared according to literature procedures 3
   1.3 Coupling screen 4
   1.4 General cross-coupling procedure 5
   1.5 Procedures and characterization data 5
2. X-ray crystallographic data 18
3. Copies of NMR spectra 21
4. References 75

1. Experimental procedures

1.1 General comments

NMR Spectroscopy: Proton (^{1}H), carbon (^{13}C), fluorine (^{19}F) and phosphorus (^{31}P) NMR spectra were recorded on Bruker AVIII HD 400, NEO 400, AVIII HD 500, AVII 500, AVII 600 or AVII 700 spectrometers. ^{1}H, ^{13}C, ^{19}F and ^{31}P chemical shifts (δ) are quoted in parts per million (ppm). ^{1}H NMR spectra were recorded using an internal deuterium lock for the residual protons in chloroform-*d* (δ = 7.26) or benzene-d_6 (δ = 7.16). ^{13}C NMR spectra were recorded using an internal deuterium lock in chloroform-*d* (δ = 77.16) or benzene-d_6 (δ = 128.06). Assignments were determined either on the basis of unambiguous chemical shift or coupling patterns, COSY, HSQC, HMBC and/or NOESY experiments. Peak multiplicities are defined as s (singlet), d (doublet), t (triplet), q (quartet), quin (quintet), sept (septet), m (multiplet) and br (broad). Coupling constants (*J*) are reported to the nearest 0.1 Hz.

Mass Spectroscopy: High-resolution mass spectra (HRMS) were recorded by the Departmental Mass Spectrometry Service, University of Oxford on a Thermo Scientific Exactive Mass Spectrometer (Waters Equity autosampler and pump) for electrospray ionization (ESI) and an Agilent 7200 Accurate Mass QTOF GCMS (using a SIM Direct Insertion Probe) for electron ionization (EI) and chemical ionization (CI). High resolution values are calculated to 4 decimal places from the molecular formula, and all values are within a tolerance of 5 ppm.

Infrared Spectroscopy: Infrared spectra were obtained on a Bruker Tensor 27 FT-IR spectrometer. The sample was prepared as a thin film on a diamond ATR module. Wavelengths of maximum absorbance (ν_{max}) are quoted in cm^{-1}. Only selected, characteristic IR absorption data are provided for each compound.

Chromatography: Column chromatography refers to normal phase column chromatography and was performed on silica gel obtained from Merck (Silica gel Si 60, 0.040-0.063 mm) under a positive pressure of nitrogen, using the stated solvent system. Analytical thin-layer chromatography was performed on pre-coated aluminium-backed plates (Merck Kieselgel 60 F_{254} plates) with visualization by ultraviolet light (254 nm) and/or by staining with phosphomolybdic acid or potassium permanganate. Retention factors (R_f) are reported with the solvent system in parentheses. High performance liquid chromatography (HPLC) for enantiomeric excess (*ee*) determination was carried out using a Phenomenex Lux i-Amylose-1 (250 mm x 4.6 mm ID) or DAICEL CHIRALPAK IB or IC (250 mm x 4.6 mm) column (wavelength: 210 nm or 250 nm) with purified material.

Materials/procedures: All air- or moisture-sensitive reactions were carried out in anhydrous solvents in heatgun-dried glassware under an inert atmosphere of argon or nitrogen. Light sensitive reactions were carried out under aluminium foil protection. Heating was performed using an oil bath. Dry tetrahydrofuran, CH_2Cl_2, pyridine and diethyl ether were collected fresh from an mBraun SPS-800 solvent purification system, having been passed through anhydrous alumina columns. All other commercially available reagents and solvents, where appropriate, were dried and purified before use, using standard procedures.

1.2 Compounds prepared according to literature procedures

J. Am. Chem. Soc. 2021, 143, 50, 21246

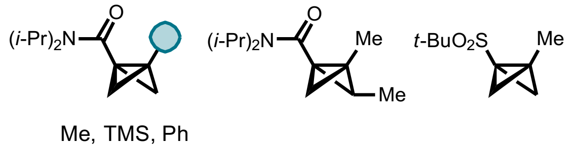

Org. Lett. 2021, 23, 9664

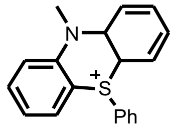

Chem. Sci., 2021, 12, 7480

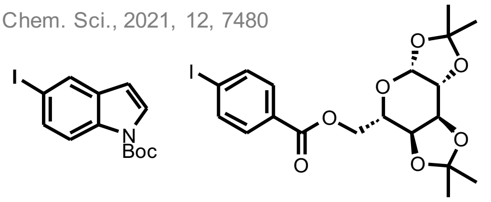

1.3 Coupling screen

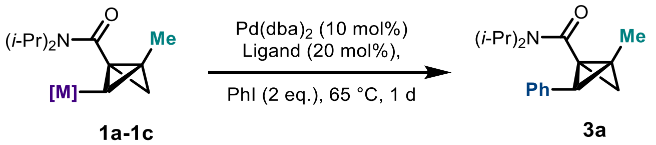

Entry[a]	Metal [M]	Ligand	Solvent (0.2 M)	Temp. (°C)	Note	Yield (%)[d]
1	$B(OH)_2$	PPh_3	THF	65	/	0
2	Sn(*n*-Bu)$_3$	$AsPh_3$	THF	65	/	0
3[b]	ZnCl.LiCl	PPh_3	THF	65	1.1 equiv. of TMEDA	0
4[c]	ZnCl.LiCl	PPh_3	THF	65	/	28
5[c]	ZnCl.LiCl	tfp	THF	65	/	27
6[c]	ZnCl.LiCl	SPhos	THF	65	/	45
7[c]	ZnCl.LiCl	DPEPhos	THF	65	/	26
8[c]	ZnCl.LiCl	Dppe	THF	65	/	4
9[c]	ZnCl.LiCl	Dppf	THF	65	/	36
10[c]	ZnCl.LiCl	P(o-tol)$_3$	THF	65	/	11
11[c]	ZnCl.LiCl	CyJPhos	THF	65	/	48
12[c]	ZnCl.LiCl	DPhos	THF	65	/	42
13[c]	ZnCl.LiCl	RuPhos	THF	65	/	35
14[c]	ZnCl.LiCl	P(*t*-Bu)$_3$	THF	65	/	42
15[c]	ZnCl.LiCl	Dcpe	THF	65	/	0
16[c]	ZnCl.LiCl	CPhos	THF	65	/	40
17[c]	ZnCl.LiCl	XPhos	THF	65	/	40
18[c]	ZnCl.LiCl	CyJPhos	THF	65	20 mol% Pd(dba)$_2$ and 40 mol% CyJPhos	61
19[c]	ZnCl.LiCl	CyJPhos	THF	65	4 equiv. of PhI	60
20[c]	ZnCl.LiCl	CyJPhos	Toluene	110	Complete decomp.	0
21[c]	ZnCl.LiCl	CyJPhos	1,4-dioxane	110	Complete decomp.	0
22[c]	ZnCl.LiCl	CyJPhos	DMF	110	Significant decomp.	n.d.
23[c]	ZnCl.LiCl	CyJPhos	THF	85	/	67
24[c]	ZnCl.LiCl	CyJPhos	THF	rt	/	6
25[c]	ZnCl.LiCl	CyJPhos	THF	65	15 mol% Pd(dba)$_2$ and 30 mol% CyJPhos	71*

Table 1. [a]Cross-coupling run on 0.1 mmol scale; Organozinc was prepared from **2a** using 1.1 equiv. of *s*-BuLi[b]/*t*-BuLi[c] and $ZnCl_2$ in THF; [d]Yield determined from the ^1H NMR spectrum of the crude reaction mixture using trimethoxybenzene as an internal standard; temp. = temperature; decomp. = decomposition; n.d. = not determined; rt = room temperature. *On scale-up it was observed that the reaction failed (immediate formation of Pd black) or yield was significantly reduced if heated directly after sample preparation. Stirring the mixture for 1 h at rt before heating resolved this issue.

1.4 General cross-coupling procedure

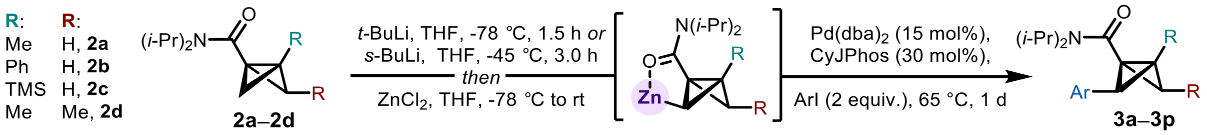

To a solution of BCB (0.20 mmol, 1.0 eq.) in THF (0.5 mL) was added *t*-BuLi (1.0 M in pentane, 0.22 mL, 1.1 eq.) dropwise at –78 °C.[A] The mixture was stirred for 1.5 h at –78 °C before addition of a solution of $ZnCl_2$ (30 mg, 0.22 mmol, 1.1 eq.) in THF (0.5 mL) at –78 °C. The mixture was stirred for 5 min at –78 °C before bringing to rt and stirring for a further 5-10 min. The solution of organozinc was transferred via syringe to a vial containing $Pd(dba)_2$ (17.0 mg, 0.03 mol, 15 mol%), CyJohnPhos (21 mg, 0.06 mmol, 30 mol%) and the coupling partner[B] (0.4 mmol, 2.0 eq.). The reaction mixture was stirred for 1 h at rt before bringing to 65 °C and stirring for 1 d. The mixture was diluted with Et_2O (5 mL), washed with water (5 mL), dried with $MgSO_4$ and filtered. The filtrate was evaporated in vacuo and the residue purified by flash chromatography.

Note A: *t*-BuLi was added dropwise directly into the solution. Running *t*-BuLi down the side of the vessel causes a build-up of white solid that takes considerable time to dissolve and can lead to incomplete metalation. Compounds **2c** and **2d** were metalated using *s*-BuLi (1.0 M in cyclohexane, 0.22 mL, 1.1 eq.) at –45 °C for 3 h.

Note B: If coupling partner is a solid, or liquid with unknown density, it was added to the vial before addition of the organozinc. If the liquid had a known density it was added after addition of the organozinc.

1.5 Procedures and characterization data

(1*S,3*S**)-*N*,*N*-diisopropyl-3-methyl-2-phenylbicyclo[1.1.0]butane-1-carboxamide, 3a**

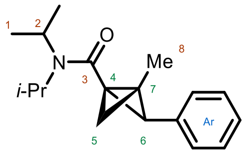

Prepared according to the general procedure using iodobenzene (44 µL, 0.4 mmol, 2.0 eq.) as coupling partner. The residue was purified *via* flash chromatography (1st 10→30% Et_2O in pentane, 1% NEt_3; 2nd 20→80% MeCN in water on Biotage® SNAP Ultra C18 12 g) to yield the title compound as a yellow oil (37 mg, 0.13 mmol, 67%).

R_f = 0.20 (20% Et_2O in pentane)

IR (thin film, ν_{max} / cm^{-1}): 1624, 1499, 1369, 1329

HRMS (ESI+) calc. for $C_{18}H_{26}ON$ $[M+H]^+$ 272.2009, found 272.2009

^{1}H NMR (600 MHz, Chloroform-*d*): δ 7.31–7.25 (m, 2H, Ar), 7.20 (tt, *J* = 6.8 Hz, 1H, Ar), 7.13–7.08 (m, 2H, Ar), 4.47 (sept, *J* = 6.7 Hz, 1H, 2), 3.25 (sept, *J* = 6.8 Hz, 1H, 2), 2.03 (s, 1H, 6), 1.82 (s, 1H, 5-exo), 1.66 (s, 3H, 8), 1.48 (d, *J* = 6.8 Hz, 3H, 1), 1.35 (d, *J* = 6.8 Hz, 3H, 1), 1.10 (d, *J* = 6.7 Hz, 3H, 1), 0.92 (s, 1H, 5-endo), 0.34 (d, *J* = 6.7 Hz, 3H, 1)

^{13}**C NMR** (151 MHz, Chloroform-*d*): δ 168.0 (3), 136.1 (Ar), 128.2 (Ar), 128.0 (Ar), 126.7 (Ar), 50.9 (6), 49.1 (2), 45.8 (2), 34.1 (5), 21.7 (1), 21.3 (1), 20.9 (4), 20.0 (1), 19.5 (1), 17.4 (7), 5.8 (8)

(1*S,3*S**)-*N*,*N*-diisopropyl-3-methyl-2-(4-nitrophenyl)bicyclo[1.1.0]butane-1-carboxamide, 3b**

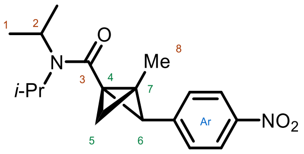

Prepared according to the general procedure using 1-iodo-4-nitrobenzene (100 mg, 0.4 mmol, 2.0 eq.) as coupling partner. The residue was purified *via* flash chromatography (5→40% EtOAc in pentane, 1% NEt_3) to yield the title compound as an orange-brown oil (40 mg, 0.13 mmol, 63%).

$\boldsymbol{R_f}$ = 0.3 (20% EtOAc in pentane)

IR (thin film, ν_{max} / cm^{-1}): 1625, 1532, 1444, 1346

HRMS (ESI^+) calc. for $C_{18}H_{25}O_3N_2$ $[M+H]^+$ 317.1860, found 317.1858

^{1}**H NMR** (600 MHz, Chloroform-*d*): δ 8.15 (d, *J* = 8.8 Hz, 2H, Ar), 7.29 (d, *J* = 8.8 Hz, 2H, Ar), 4.43 (sept, *J* = 6.8 Hz, 1H, 2), 3.28 (sept, *J* = 6.8 Hz, 1H, 2), 2.10 (s, 1H, 6), 1.82 (s, 1H, 5-exo), 1.65 (s, 3H, 8), 1.47 (d, *J* = 6.8 Hz, 3H, 1), 1.33 (d, *J* = 6.8 Hz, 3H, 1), 1.13 (d, *J* = 6.7 Hz, 3H, 1), 0.97 (s, 1H, 5-endo), 0.45 (d, *J* = 6.7 Hz, 3H, 1)

^{13}**C NMR** (151 MHz, Chloroform-*d*): δ 166.9 (3), 146.8 (Ar), 143.8 (Ar), 128.6 (Ar), 123.5 (Ar), 50.0 (6 and 2), 46.0 (2), 33.9 (5), 21.9 (4), 21.5 (1), 21.1 (1), 20.2 (1), 20.1 (1), 18.8 (7), 5.7 (8)

(1*S,3*S**)-*N*,*N*-diisopropyl-3-methyl-2-(4-(trifluoromethyl)phenyl)bicyclo[1.1.0]butane-1-carboxamide, 3c**

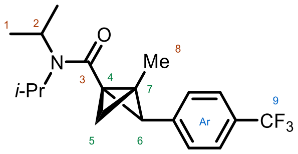

Prepared according to the general procedure using 1-iodo-4-(trifluoromethyl)benzene (59 µL, 0.4 mmol, 2.0 eq.) as coupling partner. The residue was purified *via* flash chromatography (5→30% Et_2O in pentane, 1% NEt_3) to yield the title compound as a colourless solid (57 mg, 0.17 mmol, 84%).

$\boldsymbol{R_f}$ = 0.21 (20% Et_2O in pentane)

IR (thin film, ν_{max} / cm^{-1}): 1626, 1330, 1130

HRMS (ESI^+) calc. for $C_{19}H_{25}ONF_3$ $[M+H]^+$ 340.1884, found 340.1883

^{1}**H NMR** (600 MHz, Chloroform-*d*): δ 7.53 (d, *J* = 8.1 Hz, 2H, Ar), 7.23 (d, *J* = 8.1 Hz, 2H, Ar), 4.44 (sept, *J* = 6.7 Hz, 1H, 2), 3.27 (sept, *J* = 6.8 Hz, 1H, 2), 2.06 (s, 1H, 6), 1.82 (s, 1H, 5-exo), 1.64 (s, 3H, 8), 1.47 (d, *J* = 6.8 Hz, 3H, 1), 1.33 (d, *J* = 6.9 Hz, 3H, 1), 1.11 (d, *J* = 6.8 Hz, 3H, 1), 0.94 (s, 1H, 5-endo), 0.40 (d, *J* = 6.7 Hz, 3H, 1)

^{13}**C NMR** (151 MHz, Chloroform-*d*): δ 167.2 (3), 140.1 (Ar), 128.9 (q, *J* = 32.6 Hz, Ar), 125.0 (q, *J* = 3.8 Hz, Ar), 124.2 (q, *J* = 271.9 Hz, 9), 50.0 (6), 49.8 (2), 45.8 (2), 33.9 (5), 21.4 (1), 21.2 (4), 21.0 (1), 20.0 (1), 19.7 (1), 17.9 (7), 5.6 (8)

(1*S,3*S**)-2-(4-chlorophenyl)-*N*,*N*-diisopropyl-3-methylbicyclo[1.1.0]butane-1-carboxamide, 3d**

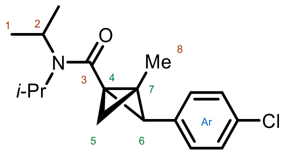

Prepared according to the general procedure using 1-chloro-4-iodobenzene (96 mg, 0.4 mmol, 2.0 eq.) as coupling partner. The residue was purified *via* flash chromatography (5→30% Et_2O in pentane, 1% NEt_3) to yield the title compound as a colourless solid (38 mg, 0.12 mmol, 60%).

R$_f$ = 0.23 (20% Et_2O in pentane)

IR (thin film, ν_{max} / cm^{-1}): 1625, 1495, 1443, 1369, 1328

HRMS (ESI$^+$) calc. for $C_{18}H_{25}O_1N^{35}Cl$ $[M+H]^+$ 306.1619, found 306.1619

^{1}**H NMR** (600 MHz, Chloroform-*d*): δ 7.25 (d, *J* = 9.0 Hz, 2H, Ar), 7.04 (d, *J* = 8.7 Hz, 2H, Ar), 4.46 (sept, *J* = 6.7 Hz, 1H, 2), 3.27 (sept, *J* = 6.8 Hz, 1H, 2), 1.99 (s, 1H, 6), 1.80 (s, 1H, 5-exo), 1.62 (s, 3H, 8), 1.47 (d, *J* = 6.8 Hz, 3H, 1), 1.33 (d, *J* = 6.8 Hz, 3H, 1), 1.11 (d, *J* = 6.7 Hz, 3H, 1), 0.91 (s, 1H, 5-endo), 0.44 (d, *J* = 6.7 Hz, 3H, 1)

^{13}**C NMR** (151 MHz, Chloroform-*d*): δ 167.6 (3), 134.7 (Ar), 132.6 (Ar), 129.2 (Ar), 128.4 (Ar), 50.1 (6), 49.9 (2), 45.9 (2), 34.0 (5), 21.6 (1), 21.2 (1), 20.9 (4), 20.1 (1), 19.9 (1), 17.6 (7), 5.7 (8)

(1*S,3*S**)-*N*,*N*-diisopropyl-2-(4-methoxyphenyl)-3-methylbicyclo[1.1.0]butane-1-carboxamide, 3e**

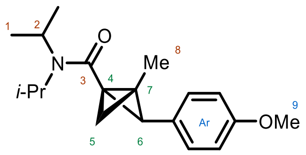

Prepared according to the general procedure using 1-iodo-4-methoxybenzene (94 mg, 0.4 mmol, 2.0 eq.) as coupling partner. The residue was purified *via* flash chromatography (5→40% Et_2O in pentane, 1% NEt_3) to yield the title compound as light brown solid (39 mg, 0.13 mmol, 65%).

R$_f$ = 0.36 (20% EtOAc in pentane)

IR (thin film, ν_{max} / cm^{-1}): 1621, 1517, 1443, 1329, 1252

HRMS (ESI$^+$) calc. for $C_{19}H_{28}O_2N$ [M+H]$^+$ 302.2115, found 302.2113

^{1}H NMR (600 MHz, Chloroform-*d*): δ 7.02 (d, *J* = 8.7 Hz, 2H, Ar), 6.82 (d, *J* = 8.7 Hz, 2H, Ar), 4.49 (sept, *J* = 6.7 Hz, 1H, 2), 3.78 (s, 3H, 9), 3.25 (sept, *J* = 6.8 Hz, 1H, 2), 2.00 (s, 1H, 6), 1.80 (s, 1H, 5-exo), 1.63 (s, 3H, 8), 1.47 (d, *J* = 6.7 Hz, 3H, 1), 1.34 (d, *J* = 6.8 Hz, 3H, 1), 1.10 (d, *J* = 6.7 Hz, 3H, 1), 0.89 (s, 1H, 5-endo), 0.40 (d, *J* = 6.7 Hz, 3H, 1)

^{13}C NMR (151 MHz, Chloroform-*d*): δ 168.2 (3), 158.6 (Ar), 129.0 (Ar), 128.4 (Ar), 113.6 (Ar), 55.4 (9), 50.4 (6), 49.8 (2), 45.8 (2), 34.0 (5), 21.7 (1), 21.3 (1), 20.6 (4), 20.1 (1), 19.7 (1), 17.1 (7), 5.8 (8)

(1*S,3*S**)-2-(2-cyanophenyl)-*N*,*N*-diisopropyl-3-methylbicyclo[1.1.0]butane-1-carboxamide, 3f**

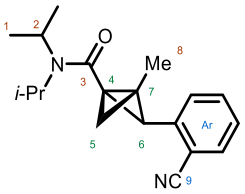

Prepared according to the general procedure using 2-iodobenzonitrile (92 mg, 0.4 mmol, 2.0 eq.) as coupling partner. The residue was purified *via* flash chromatography (5→30% Et_2O in pentane, 1% NEt_3) and cold pentane trituration to yield the title compound as a cream solid (40 mg, 0.14 mmol, 68%).

R$_f$ = 0.24 (40% Et_2O in pentane)

IR (thin film, ν_{max} / cm^{-1}): 2224, 1624, 1447, 1328

HRMS (ESI$^+$) calc. for $C_{19}H_{25}ON_2$ [M+H]$^+$ 297.1961, found 297.1962

^{1}H NMR (600 MHz, Chloroform-*d*): δ 7.63 (d, *J* = 7.7 Hz, 1H, Ar), 7.50 (t, *J* = 7.8 Hz, 1H, Ar), 7.30 (t, *J* = 7.6 Hz, 1H, Ar), 7.15 (d, *J* = 8.1 Hz, 1H, Ar), 4.46 (sept, *J* = 6.7 Hz, 1H, 2), 3.27 (sept, *J* = 6.8 Hz, 1H, 2), 2.48 (s, 1H, 6), 1.80 (s, 1H, 5-exo), 1.65 (s, 3H, 8), 1.45 (d, *J* = 6.8 Hz, 3H, 1), 1.30 (d, *J* = 6.8 Hz, 3H, 1), 1.13 (d, *J* = 6.7 Hz, 3H, 1), 1.02 (s, 1H, 5-endo), 0.46 (d, *J* = 6.7 Hz, 3H, 1)

^{13}C NMR (151 MHz, Chloroform-*d*): δ 167.0 (3), 139.7 (Ar), 133.2 (Ar), 132.3 (Ar), 128.2 (Ar), 127.0 (Ar), 118.1 (9), 112.1 (Ar), 50.0 (6), 47.2 (2), 45.9 (2), 33.5 (5), 21.5 (1), 21.3 (4), 21.1 (1), 20.1 (1), 19.8 (1), 18.5 (7), 5.7 (8)

(1*S,3*S**)-*N*,*N*-diisopropyl-3-methyl-2-(o-tolyl)bicyclo[1.1.0]butane-1-carboxamide, 3g**

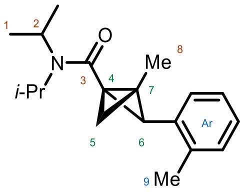

Prepared according to the general procedure using 1-iodo-2-methylbenzene (51 µL, 0.4 mmol, 2.0 eq.) as coupling partner. The residue was purified *via* flash chromatography (1st 5→30% Et_2O in pentane, 1% NEt_3; 2nd 2→8% acetone in toluene, 1% NEt_3) to yield the title compound as a colourless oil (41 mg, 0.14 mmol, 72%).

***R*~f~** = 0.20 (20% Et_2O in pentane)

IR (thin film, ν_{max} / cm^{-1}):1626, 1443, 1328

HRMS (ESI^+) calc. for $C_{19}H_{28}ON$ $[M+H]^+$ 286.2165, found 286.2162

^{1}H NMR (600 MHz, Chloroform-*d*): δ 7.13 (d, *J* = 7.0 Hz, 1H, Ar), 7.12 – 7.05 (m, 2H, Ar), 6.91 (dd, *J* = 7.4, 1.7 Hz, 1H, Ar), 4.49 (sept, *J* = 6.7 Hz, 1H, 2), 3.28 (sept, *J* = 6.8 Hz, 1H, 2), 2.38 (s, 3H, 9), 2.15 (s, 1H, 6), 1.79 (s, 1H, 5-exo), 1.62 (s, 3H, 8), 1.47 (d, *J* = 6.8 Hz, 3H, 1), 1.36 (d, *J* = 6.8 Hz, 3H, 1), 1.12 (d, *J* = 6.7 Hz, 3H, 1), 0.90 (s, 1H, 5-endo), 0.46 (d, *J* = 6.7 Hz, 3H, 1)

^{13}C NMR (151 MHz, Chloroform-*d*): δ 168.3 (3), 136.1 (Ar), 134.0 (Ar), 130.4 (Ar), 127.6 (Ar), 126.3 (Ar), 125.6 (Ar), 49.8 (6), 46.3 (2), 45.8 (2), 33.9 (5), 21.7 (1), 21.3 (1), 20.7 (4), 20.2 (9), 20.0 (1), 19.6 (1), 17.1 (7), 6.1 (8)

((3a*R*,5*R*,5a*S*,8a*S*,8b*R*)-2,2,7,7-tetramethyltetrahydro-5*H*-bis([1,3]dioxolo)[4,5-b:4',5'-d]pyran-5-yl)methyl 4-((1*S*/*R,2*S*/*R**,3*S*/*R**)-1-(diisopropylcarbamoyl)-3-methylbicyclo[1.1.0]butan-2-yl)benzoate, 3h**

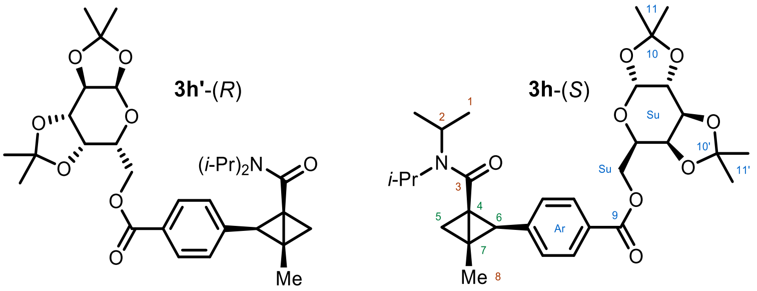

Prepared according to the general procedure using ((3a*R*,5*S*,5a*S*,8a*S*,8b*R*)-2,2,7,7-tetramethyltetrahydro-5*H*-bis([1,3]dioxolo)[4,5-b:4',5'-*d*]pyran-5-yl)methyl 4-iodobenzoate (196 mg, 0.4 mmol, 2.0 eq.) as coupling partner. The residue was purified *via* flash chromatography (1st 20→65% Et_2O in pentane, 1% NEt_3; 2nd 5→10% acetone in toluene, 1% NEt_3) to yield the title compound as a colourless oil (103 mg, 0.18 mmol, 91%).

***R*~f~** = 0.29 (60% Et_2O in pentane)

IR (thin film, ν_{max} / cm^{-1}): 1719, 1614, 1276, 1070

HRMS (ESI$^+$) calc. for $C_{31}H_{44}O_8N$ $[M+H]^+$ 558.3061, found 558.3058

^{1}H NMR (600 MHz, Chloroform-*d*): δ 7.96 (d, *J* = 8.2 Hz, 2H Ar), 7.17 (d, *J* = 8.2 Hz, 2H, Ar), 5.56 (d, *J* = 2.6 Hz, 0.5H, Su), 5.55 (d, *J* = 2.6 Hz, 0.5H, Su), 4.65 (d, *J* = 7.9 Hz, 1H, Su), 4.54 – 4.39 (m, 3H, 2 and Su), 4.38 – 4.29 (m, 2H, Su), 4.20 – 4.15 (m, 1H, Su), 3.26 (sept, *J* = 6.8 Hz, 1H, 2), 2.06 (s, 1H, 6), 1.82 (s, 1H, 5-exo), 1.64 (s, 3H, 8), 1.51 (d, *J* = 6.9 Hz, 3H, 1), 1.48 – 1.45 (m, 6H, 1 and 11/11'), 1.37 – 1.32 (m, 9H, 11/11'), 1.10 (d, *J* = 6.7 Hz, 3H, 1), 0.94 (s, 1H, 5-endo), 0.39 (d, *J* = 3.3 Hz, 1.5H, 1), 0.38 (d, *J* = 3.3 Hz, 1.5H, 1). 1:1 mixture of BCB diastereomers.

^{13}C NMR (151 MHz, Chloroform-*d*): δ 167.47 (3), 167.45 (3), 166.5 (9), 166.4 (9), 141.5 (Ar), 129.7 (Ar), 128.5 (Ar), 127.9 (Ar), 109.88 (10/10'), 109.85 (10/10'), 108.98 (10/10'), 108.95 (10/10'), 96.5 (Su), 71.4 (Su), 71.3 (Su), 70.9 (Su), 70.7 (Su), 66.4 (Su), 64.11 (Su), 64.07 (Su), 50.52 (6), 50.51 (6), 49.9 (2), 45.9 (2), 34.1 (5), 26.2 (1), 26.1 (11/11'), 25.2 (11/11'), 24.6 (11/11'), 21.60 (1), 21.58 (4), 21.56 (4), 21.21 (1), 20.15 (11/11'), 20.10 (11/11'), 19.9 (1), 18.2 (7), 18.1 (7), 5.8 (8). Mixture contains overlapping signals in the ^{13}C NMR spectrum.

Ethyl (*Z*)-3-((1*S,3*S**)-1-(diisopropylcarbamoyl)-3-methylbicyclo[1.1.0]butan-2-yl)acrylate, 3i**

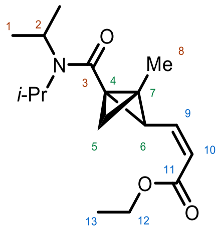

Prepared according to the general procedure using ethyl (*Z*)-3-iodoacrylate (51 µL, 0.4 mmol, 2.0 eq.) as coupling partner. The residue was purified *via* flash chromatography (10→20% EtOAc in pentane, 1% NEt_3 and 5→30% Et_2O in pentane, 1% NEt_3) to yield the title compound as a yellow oil (38 mg, 0.13 mmol, 64%).

R$_f$ = 0.19 (20% Et_2O in pentane)

IR (thin film, ν_{max} / cm^{-1}): 1720, 1628, 1444, 1334, 1189

HRMS (ESI$^+$) calc. for $C_{17}H_{28}O_3N$ $[M+H]^+$ 294.2064, found 294.2065

^{1}H NMR (600 MHz, Chloroform-*d*): δ 6.18 (dd, *J* = 11.8, 10.6 Hz, 1H, 9), 5.80 (d, *J* = 11.8 Hz, 1H, 10), 4.62 (sept, *J* = 6.6 Hz, 1H, 2), 4.17 (qd, *J* = 7.1, 3.0 Hz, 2H, 12), 3.39 (sept, *J* = 6.8 Hz, 1H, 2), 3.01 (d, *J* = 10.6 Hz, 1H, 6), 1.68 (s, 1H, 5-exo), 1.64 (s, 3H, 8), 1.42 (d, *J* = 6.8 Hz, 3H, 1), 1.38 (d, *J* = 6.8 Hz, 3H, 1), 1.28 (t, *J* = 7.1 Hz, 3H, 13), 1.16 (d, *J* = 6.7 Hz, 3H, 1), 1.13 (d, *J* = 6.7 Hz, 3H, 1), 0.95 (s, 1H, 5-endo)

^{13}C NMR (151 MHz, Chloroform-*d*): δ 167.6 (3), 166.4 (11), 143.3 (9), 120.2 (10), 60.1 (12), 49.8 (2), 45.9 (2), 45.6 (6), 34.6 (5), 22.7 (4), 21.4 (1), 20.9 (1), 18.1 (7), 14.4 (13), 8.3 (8)

(1*S,3*S**)-*N*,*N*-diisopropyl-3-methyl-2-(pyridin-2-yl)bicyclo[1.1.0]butane-1-carboxamide, 3j**

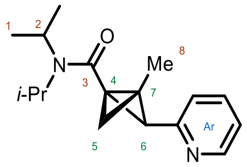

Prepared according to the general procedure using 2-iodopyridine (43 µL, 0.4 mmol, 2.0 eq.) as coupling partner. The residue was purified *via* flash chromatography (10→40% EtOAc in pentane, 1% NEt_3) to yield the title compound as a yellow oil (46 mg, 0.17 mmol, 84%).

***R**$_f$* = 0.30 (40% EtOAc in pentane)

IR (thin film, ν_{max} / cm^{-1}): 1625, 1442, 1330

HRMS (ESI^+) calc. for $C_{17}H_{25}ON_2$ $[M+H]^+$ 273.1961, found 273.1961

^{1}H NMR (600 MHz, Chloroform-*d*): δ 8.51 (d, *J* = 4.8 Hz, 1H, Ar), 7.58 (td, *J* = 7.7, 1.8 Hz, 1H, Ar), 7.11 (dd, *J* = 7.5, 4.8 Hz, 1H, Ar), 7.04 (d, *J* = 8.0 Hz, 1H, Ar), 4.50 (sept, *J* = 6.7 Hz, 1H, 2), 3.25 (sept, *J* = 6.8 Hz, 1H, 2), 2.27 (s, 1H, 6), 1.80 (s, 1H, 5-exo), 1.68 (s, 3H, 8), 1.46 (d, *J* = 6.8 Hz, 3H, 1), 1.33 (d, *J* = 6.8 Hz, 3H, 1), 1.10 (d, *J* = 6.8 Hz, 3H, 1), 0.96 (s, 1H, 5-endo), 0.34 (d, *J* = 6.7 Hz, 3H, 1)

^{13}C NMR (151 MHz, Chloroform-*d*): δ 167.7 (3), 155.7 (Ar), 149.2 (Ar), 135.8 (Ar), 122.2 (Ar), 121.7 (Ar), 51.9 (6), 49.7 (2), 45.8 (2), 33.9 (5), 21.6 (1), 21.3 (4), 21.3 (1), 20.2 (1), 19.5 (1), 18.0 (7), 6.1 (8)

***Tert-butyl* 5-((1S*,3S*)-1-(diisopropylcarbamoyl)-3-methylbicyclo[1.1.0]butan-2-yl)-1*H*-indole-1-carboxylate, 3k**

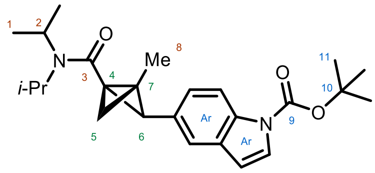

Prepared according to the general procedure using *tert*-butyl 5-iodo-1*H*-indole-1-carboxylate (137 mg, 0.4 mmol, 2.0 eq.) as coupling partner. The residue was purified *via* flash chromatography (5→40% Et_2O in pentane, 1% NEt_3) to yield the title compound as a colourless semi-solid (41 mg, 0.1 mmol, 50%).

***R**$_f$* = 0.23 (30% Et_2O in pentane)

IR (thin film, ν_{max} / cm^{-1}): 1619, 1327, 1070

HRMS (ESI^+) calc. for $C_{25}H_{35}O_3N_2$ $[M+H]^+$ 411.2642, found 411.2618

^{1}H NMR (600 MHz, Chloroform-*d*): δ 8.02 (d, *J* = 8.5 Hz, 1H, Ar), 7.55 (d, *J* = 3.7 Hz, 1H, Ar), 7.25 (d, *J* = 1.8 Hz, 1H, Ar), 7.07 (dd, *J* = 8.6, 1.8 Hz, 1H, Ar), 6.48 (d, *J* = 3.6 Hz, 1H, Ar), 4.52 (sept, *J* = 6.7 Hz, 1H, 2), 3.22 (sept, *J* = 6.8

Hz, 1H, 2), 2.13 (s, 1H, 6), 1.84 (s, 1H, 5-exo), 1.66 (s, 3H, 8), 1.66 (s, 9H, 11), 1.48 (d, *J* = 6.8 Hz, 3H, 1), 1.35 (d, *J* = 6.8 Hz, 3H, 1), 1.08 (d, *J* = 6.7 Hz, 3H, 1), 0.93 (s, 1H, 5-endo), 0.27 (d, *J* = 6.6 Hz, 3H, 1)

^{13}C NMR (151 MHz, Chloroform-*d*): δ 168.3 (3), 149.8 (9), 134.2 (Ar), 130.5 (Ar), 126.4 (Ar), 124.6 (Ar), 120.0 (Ar), 114.7 (Ar), 107.3 (Ar), 83.8 (10), 50.9 (6), 49.9 (2), 45.8 (2), 34.0 (5), 28.3 (11), 21.6 (1), 21.3 (1), 21.0 (4), 19.9 (1), 19.8 (1), 17.2 (7), 5.8 (8)

(1*S,3*S**)-2-(7-chloroquinolin-4-yl)-*N*,*N*-diisopropyl-3-methylbicyclo[1.1.0]butane-1-carboxamide, 3l**

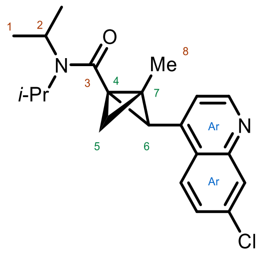

Prepared according to the general procedure using 7-chloro-4-iodoquinoline (116 mg, 0.4 mmol, 2.0 eq.) as coupling partner. The residue was purified *via* flash chromatography (10→40% EtOAc in pentane, 1% NEt_3 and 20→80% Et_2O in pentane, 1% NEt_3) to yield the title compound as a cream solid (48 mg, 0.14 mmol, 68%).

R_f = 0.21 (30% EtOAc in pentane)

IR (thin film, ν_{max} / cm^{-1}): 1625, 1446, 1327

HRMS (ESI^+) calc. for $C_{21}H_{26}ON_2Cl$ $[M+H]^+$ 357.1726, found 357.1726

^{1}H NMR (600 MHz, Chloroform-*d*): δ 8.80 (d, *J* = 4.7 Hz, 1H, Ar), 8.16 (d, *J* = 9.1 Hz, 1H, Ar), 8.11 (d, *J* = 2.2 Hz, 1H, Ar), 7.53 (dd, *J* = 9.0, 2.2 Hz, 1H, Ar), 7.08 (d, *J* = 4.6 Hz, 1H, Ar), 4.50 (sept, *J* = 6.7 Hz, 1H, 2), 3.34 (sept, *J* = 6.8 Hz, 1H, 2), 2.63 (s, 1H, 6), 1.86 (s, 1H, 5-exo), 1.62 (s, 3H, 8), 1.48 (d, *J* = 6.8 Hz, 3H, 1), 1.40 (d, *J* = 6.8 Hz, 3H, 1), 1.13 (d, *J* = 6.7 Hz, 3H, 1), 1.08 (s, 1H, 5-endo), 0.51 (d, *J* = 6.7 Hz, 3H, 1)

^{13}C NMR (151 MHz, Chloroform-*d*): δ 166.9 (3), 150.8 (Ar), 149.1 (Ar), 141.6 (Ar), 135.3 (Ar), 129.4 (Ar), 127.8 (Ar), 125.9 (Ar), 124.8 (Ar), 119.9 (Ar), 50.0 (6), 46.1 (2), 44.3 (2), 34.0 (5), 22.1 (4), 21.4 (1), 21.1 (1), 20.3 (1), 20.2 (1), 17.9 (7), 6.1 (8)

Single Crystal Data for **3l**: C21H25ClN2O, Mr =356.89. 150 K – Monoclinic, P 2_1/c, a = 10.09997(2) Å, b = 14.1102(3) Å, c = 13.8140(2) Å, β = 99.8086(17)° ,V = 1939.84(6) Å, Data/restraints/parameters – 4033/0/227, Rint = 0.020, Final R1 = 0.0323, wR2 = 0.0856 (I>2σ(I)).

Methyl 4-((1*S,3*S**)-1-(diisopropylcarbamoyl)-3-phenylbicyclo[1.1.0]butan-2-yl)benzoate, 3m**

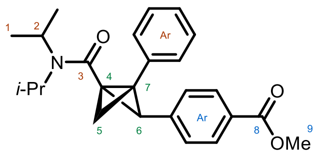

Prepared according to the general procedure using methyl 4-iodobenzoate (105 mg, 0.4 mmol, 2.0 eq.) as coupling partner. The residue was purified *via* flash chromatography (10→40% Et_2O in pentane, 1% NEt_3) followed by trituration with cold pentane to yield the title compound as a colourless solid (50 mg, 0.13 mmol, 64%).

***R*$_f$** = 0.27 (30% Et_2O in pentane)

IR (thin film, ν_{max} / cm^{-1}): 1723, 1626, 1442, 1336, 1280

HRMS (ESI^+) calc. for $C_{25}H_{30}O_3N$ $[M+H]^+$ 392.2220, found 392.2213

^{1}H NMR (600 MHz, Chloroform-*d*): δ 7.83 (d, *J* = 8.3 Hz, 2H, Ar), 7.31 (d, *J* = 7.2 Hz, 2H, Ar), 7.20 (t, *J* = 7.7 Hz, 2H, Ar), 7.15 (t, *J* = 7.3 Hz, 1H, Ar), 7.07 (d, *J* = 8.3 Hz, 2H, Ar), 4.38 (sept, *J* = 6.7 Hz, 1H, 2), 3.87 (s, 3H, 9), 3.22 (sept, *J* = 6.8 Hz, 1H, 2), 2.49 – 2.48 (m, 2H, 6, 5-exo), 1.48 (d, *J* = 6.8 Hz, 3H, 1), 1.37 (s, 1H, 5-endo), 1.22 (d, *J* = 6.8 Hz, 3H, 1), 1.13 (d, *J* = 6.7 Hz, 3H, 1), 0.30 (d, *J* = 6.6 Hz, 3H, 1)

^{13}C NMR (151 MHz, Chloroform-*d*): δ 167.1 (8), 165.9 (3), 140.8 (Ar), 131.7 (Ar), 129.6 (Ar), 129.3 (Ar), 128.7 (Ar), 128.3 (Ar), 128.2 (Ar), 126.4 (Ar), 52.2 (9), 50.4 (6), 49.9 (2), 46.0 (2), 35.7 (5), 28.4 (4), 28.2 (7), 21.8 (1), 21.2 (1), 20.0 (1), 19.8 (1)

(1*S,3*S**)-*N*,*N*-diisopropyl-2,3-diphenylbicyclo[1.1.0]butane-1-carboxamide, 3n**

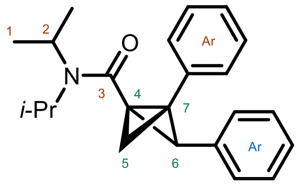

Prepared according to the general procedure using iodobenzene (0.2 mL, 0.4 mmol, 2.0 eq.) as coupling partner. The residue was purified *via* flash chromatography (10→40% Et_2O in pentane, 1% NEt_3) to yield the title compound as an off-white solid (37 mg, 0.11 mmol, 55%).

***R*$_f$** = 0.34 (20% Et_2O in pentane)

IR (thin film, ν_{max} / cm^{-1}): 3032, 2960, 2929, 1626, 1446

HRMS (ESI^+) calc. for $C_{23}H_{28}ON$ $[M+H]^+$ 334.2171, found 334.2165

^{1}**H NMR** (500 MHz, Chloroform-*d*): δ 7.36–7.34 (m, 2H, Ar), 7.22–7.19 (m, 2H, Ar), 7.15–7.12 (m, 4H, Ar), 6.99–6.95 (m, 2H, Ar), 4.40 (sept, *J* = 6.7 Hz, 1H, 2), 3.21 (sept, *J* = 6.8 Hz, 1H, 2), 2.50 (s, 1H, 6-endo), 2.45 (s, 1H, 5-exo), 1.49 (d, *J* = 6.8 Hz, 3H, 1), 1.34 (d, *J* = 1.0 Hz, 1H, 5-endo), 1.21 (d, *J* = 6.8 Hz, 3H, 1), 1.12 (d, *J* = 6.7 Hz, 3H, 1), 0.26 (d, *J* = 6.7 Hz, 3H, 1)

^{13}**C NMR** (126 MHz, Chloroform-*d*): δ 166.4 (3), 135.4 (Ar), 132.4 (Ar), 129.7 (Ar), 128.3 (Ar), 128.04 (Ar), 127.96 (Ar), 126.9 (Ar), 126.1 (Ar), 50.8 (2), 49.8 (2), 45.9 (6), 35.7 (5), 28.3 (4/7), 27.4 (4/7), 22.5 (1), 21.9 (1), 21.3 (1), 19.9 (1).

1 mmol scale

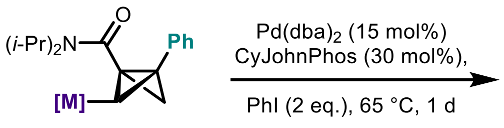

3n

We have performed 1 mmol scale reaction between *N*,*N*-diisopropyl-3-phenylbicyclo[1.1.0]butane-1-carboxamide and iodobenzene. Using general procedure, *N*,*N*-diisopropyl-3-phenylbicyclo[1.1.0]butane-1-carboxamide (257 mg, 1 mmol, 1 eq.), *t*-BuLi (1.0 M in pentane, 1.1 mL, 1.1 eq.), $ZnCl_2$ (150 mg, 1.10 mmol, 1.1 eq.), $Pd(dba)_2$ (86.0 mg, 0.15 mmol, 15 mol%), CyJohnPhos (105 mg, 0.3 mmol, 30 mol%) and iodobenzene (0.2 mL, 2.0 mmol, 2.0 eq.) were employed for the cross coupling reaction. The residue was purified *via* flash chromatography (5→20% Et_2O in pentane, 1% NEt_3) to yield the title compound as a colourless solid (170 mg, 0.51 mmol, 51%).

Methyl 4-((1*R,3*R**)-1-(diisopropylcarbamoyl)-3-(trimethylsilyl)bicyclo[1.1.0]butan-2-yl)benzoate, 3o**

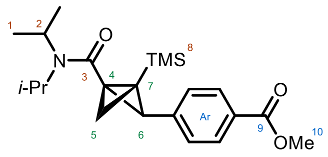

Prepared according to the general procedure using methyl 4-iodobenzoate (105 mg, 0.4 mmol, 2.0 eq.) as coupling partner. The residue was purified *via* flash chromatography (5→20% Et_2O in pentane, 1% NEt_3) to yield the title compound as a yellow solid (35 mg, 0.09 mmol, 45%).

***R**$_f$* = 0.40 (20% Et_2O in pentane)

IR (thin film, ν_{max} / cm^{-1}): 1723, 1626, 1442, 1336, 1280

HRMS (ESI^+) calc. for $C_{22}H_{34}O_3NSi$ $[M+H]^+$ 388.2302, found 388.2296

^{1}**H NMR** (600 MHz, Chloroform-*d*): δ 7.91 (d, *J* = 8.4 Hz, 2H, Ar), 7.25 (d, *J* = 8.5 Hz, 2H, Ar), 4.56 (sept, *J* = 6.7 Hz, 1H, 2), 3.89 (s, 3H, 10), 3.33 (sept, *J* = 6.8 Hz, 1H, 2), 1.83 (s, 1H, 6), 1.57 (d, *J* = 1.2 Hz, 1H, 5-exo), 1.48 (d, *J* = 6.8

Hz, 3H, 1), 1.39 (d, J = 6.8 Hz, 3H, 1), 1.13 (d, J = 6.7 Hz, 3H, 1), 0.57 (s, 1H, 5-endo), 0.51 (d, J = 6.7 Hz, 3H, 1), 0.06 (s, 9H, 8)

^{13}C NMR (151 MHz, Chloroform-*d*): δ 167.2 (3/9), 167.0 (3/9), 143.0 (Ar), 129.3 (Ar), 128.6 (Ar), 127.7 (Ar), 52.2 (10), 51.5 (6), 49.8 (2), 46.0 (2), 32.2 (5), 25.7 (4), 21.6 (1), 21.2 (1), 20.1 (1), 19.9 (1), 12.7 (7), 0.3 (8)

Methyl 4-((1*S,3*S**,4*R**)-1-(diisopropylcarbamoyl)-3,4-dimethylbicyclo[1.1.0]butan-2-yl)benzoate, 3p**

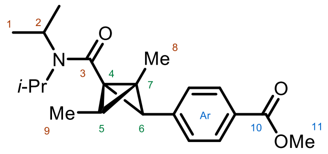

Prepared according to the general procedure using methyl 4-iodobenzoate (105 mg, 0.4 mmol, 2.0 eq.) as coupling partner. The residue was purified *via* flash chromatography (10→30% Et_2O in pentane, 1% NEt_3) followed by dissolving in a minimum amount of pentane and filtering. The organic was evaporated in vacuo to yield the title compound as a yellow oil (44 mg, 0.13 mmol, 65%).

***R*$_f$** = 0.21 (20% Et_2O in pentane)

IR (thin film, $ν_{max}$ / cm^{-1}): 1725, 1624, 1320, 1279

HRMS (ESI^+) calc. for $C_{21}H_{30}O_3N$ $[M+H]^+$ 344.2219, found 344.2219

^{1}H NMR (600 MHz, Chloroform-*d*): δ 7.93 (d, J = 8.4 Hz, 2H, Ar), 7.22 (d, J = 8.5 Hz, 2H, Ar), 4.45 (sept, J = 6.7 Hz, 1H, 2), 3.89 (s, 3H, 11), 3.25 (sept, J = 6.8 Hz, 1H, 2), 1.86 (s, 1H, 6), 1.55 (s, 3H, 8), 1.43 (d, J = 6.7 Hz, 3H, 1), 1.30 (d, J = 6.8 Hz, 3H, 1), 1.22 (d, J = 5.6 Hz, 3H, 9), 1.13 (t, J = 5.5 Hz, 1H, 5), 1.10 (d, J = 6.7 Hz, 3H, 1), 0.49 (d, J = 6.7 Hz, 3H, 1)

^{13}C NMR (151 MHz, Chloroform-*d*): δ 167.2 (3/10), 166.2 (3/10), 141.8 (Ar), 129.5 (Ar), 128.2 (Ar), 127.7 (Ar), 52.2 (11), 49.8 (2), 48.8 (6), 45.8 (2), 39.6 (5), 23.9 (4), 23.0 (7), 21.6 (1), 21.3 (1), 20.3 (1), 20.1 (1), 9.5 (9), 2.5 (8)

Methyl 4-((1*S,3*S**)-1-(*tert*-butylsulfonyl)-3-methylbicyclo[1.1.0]butan-2-yl)benzoate, 4a**

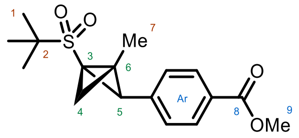

Prepared according to the general procedure using methyl 4-iodobenzoate (105 mg, 0.4 mmol, 2.0 eq.) as coupling partner. The residue was purified *via* flash chromatography (10→50% Et_2O in pentane, 1% NEt_3) followed by trituration with pentane to yield the title compound as a cream solid (52 mg, 0.16 mmol, 82%).

***R*$_f$** = 0.29 (50% Et_2O in pentane)

IR (thin film, ν_{max} / cm^{-1}): 1718, 1292, 1112

HRMS (ESI^+) calc. for $C_{17}H_{23}O_4S$ $[M+H]^+$ 323.1312, found 323.1310

^{1}H NMR (600 MHz, Chloroform-*d*): δ 8.00 (d, *J* = 8.4 Hz, 2H, Ar), 7.54 (d, *J* = 8.4 Hz, 2H, Ar), 3.90 (s, 3H, 9), 2.36 (s, 1H, 5), 2.13 (s, 1H, 4-exo), 1.96 (s, 3H, 7), 1.35 (s, 9H, 1), 1.14 (s, 1H, 4-endo)

^{13}C NMR (151 MHz, Chloroform-*d*): δ 167.0 (8), 137.8 (Ar), 129.7 (Ar), 129.3 (Ar), 129.3 (Ar), 61.2 (2), 53.9 (5), 52.2 (9), 36.9 (4), 25.0 (6/3), 24.9 (6/3), 23.5 (1), 7.4 (7)

(1*S,2*S**)-1-allyl-*N*,*N*-diisopropyl-3-methylene-2-(4-(trifluoromethyl)phenyl)cyclobutane-1-carboxamide, 6**

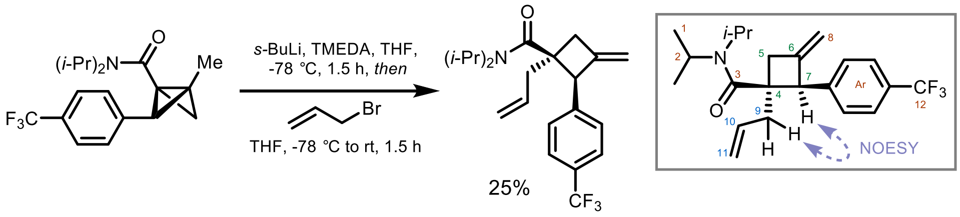

To a solution of **3c** (48 mg, 0.14 mmol, 1.0 eq.) and TMEDA (30 µL, 0.20 mmol, 1.4 eq.) in THF (0.35 mL) was added at *s*-BuLi (1.0 M in cyclohexane, 0.20 mL, 1.4 eq.) at -78 °C. The reaction was stirred for 1.5 h before the addition of allyl bromide (74 µL, 0.86 mmol, 6.0 eq.), and stirring for 30 min -78 °C. The reaction warmed to rt and stirred for 1 h before diluting with Et_2O (5 mL) and washing with water (5 mL). The organics was dried with $MgSO_4$, filtered and evaporated in vacuo. The residue was purified *via* flash chromatography (5→30% Et_2O in pentane) to yield the title compound as a colourless oil (16 mg, 13 mmol, 25%, brsm 53%).

$\boldsymbol{R_f}$ = 0.40 (20% Et_2O in pentane)

IR (thin film, ν_{max} / cm^{-1}): 1619, 1369, 1127

HRMS (ESI^+) calc. for $C_{22}H_{29}ONF_3$ $[M+H]^+$ 380.2196, found 380.2203

^{1}H NMR (600 MHz, Chloroform-*d*): δ 7.53 (d, *J* = 8.1 Hz, 2H, Ar), 7.36 (d, *J* = 8.0 Hz, 2H, Ar), 5.78 – 5.64 (m, 1H, 10), 5.21 – 5.10 (m, 2H, 11), 4.96 (d, *J* = 2.3 Hz, 1H, 8), 4.79 (d, *J* = 2.5 Hz, 1H, 8), 3.80 – 3.69 (m, 2H, 5 and 7), 3.37 (sept, *J* = 6.5 Hz, 1H, 2), 3.06 (sept, *J* = 6.8 Hz, 1H, 2), 2.76 – 2.61 (m, 2H, 8 and 9), 2.53 (dd, *J* = 14.2, 7.7 Hz, 1H, 9), 1.29 (d, *J* = 6.7 Hz, 3H, 1), 1.21 (d, *J* = 6.7 Hz, 3H, 1), 1.11 (d, *J* = 6.5 Hz, 3H, 1), 0.10 (br, 3H, 1)

^{13}C NMR (151 MHz, Chloroform-*d*): δ 170.9 (3), 147.7 (6), 145.4 (Ar), 133.0 (10), 129.23 (q, *J* = 32.3 Hz, Ar), 128.3 (Ar), 125.6 (q, *J* = 3.9 Hz, Ar), 124.3 (q, *J* = 271.9 Hz, 12), 118.5 (11), 109.3 (8), 60.6 (7), 49.6 (4), 48.5 (2), 46.3 (2), 42.8 (9), 40.0 (5), 21.4 (1), 20.4 (1), 20.1 (1), 20.0 (1)

2. X-Ray Crystallographic data

Single crystal growth: A sample of **3l** was dissolved in a minimum volume of hot Et_2O, and then allowed to cool to rt before placing in the fridge overnight. Crystals were collected from this liquor.

Single crystal X-ray diffraction data for **3l** were collected using a (Rigaku) Oxford Diffraction SuperNova diffractometer at 150 K. The single crystal was affixed to a cryoloop using Fomblin® Y oil. Raw frame data were reduced using CrysAlisPro and the structure was solved using 'Superflip'[1] before refinement with CRYSTALS.[2, 3] Refinement was by full-matrix least-squares with anisotropic atomic displacement parameters freely refined for all non-hydrogen atoms. In general, hydrogen atoms were visible in the difference Fourier map. Hydrogens bound to carbon were positioned at calculated positions and refined separately with restraints before being included in the refinement using a riding model.[3] Data for **3l** are deposited with the CCDC: CCDC 2292149.

Crystal data and structure refinement for 3l.

CCDC Identification code	CCDC 2292149	
Empirical formula	C21 H25 Cl N2 O	
Formula weight	356.89	
Temperature	150 K	
Wavelength	1.54184 Å	
Crystal system	Monoclinic	
Space group	P 21/c	
Unit cell dimensions	a = 10.0997(2) Å	α= 90°.
	b = 14.1102(3) Å	β= 99.8086(17)°.
	c = 13.8140(2) Å	γ = 90°.
Volume	1939.84(6) Å3	
Z	4	
Density (calculated)	1.222 Mg/m3	
Absorption coefficient	1.813 mm-1	
F(000)	760	
Crystal size	0.26 x 0.22 x 0.20 mm3	
Theta range for data collection	4.443 to 76.448°.	
Index ranges	-12<=h<=12, -17<=k<=14, -17<=l<=13	
Reflections collected	11727	
Independent reflections	4033 [R(int) = 0.020]	
Completeness to theta = 74.919°	99.6 %	
Absorption correction	Semi-empirical from equivalents	
Max. and min. transmission	0.70 and 0.62	
Refinement method	Full-matrix least-squares on F2	
Data / restraints / parameters	4033 / 0 / 227	
Goodness-of-fit on F2	0.9999	
Final R indices [I>2sigma(I)]	R1 = 0.0323, wR2 = 0.0856	
R indices (all data)	R1 = 0.0344, wR2 = 0.0875	
Extinction coefficient	40(6)	
Largest diff. peak and hole	0.22 and -0.21 e.Å-3	

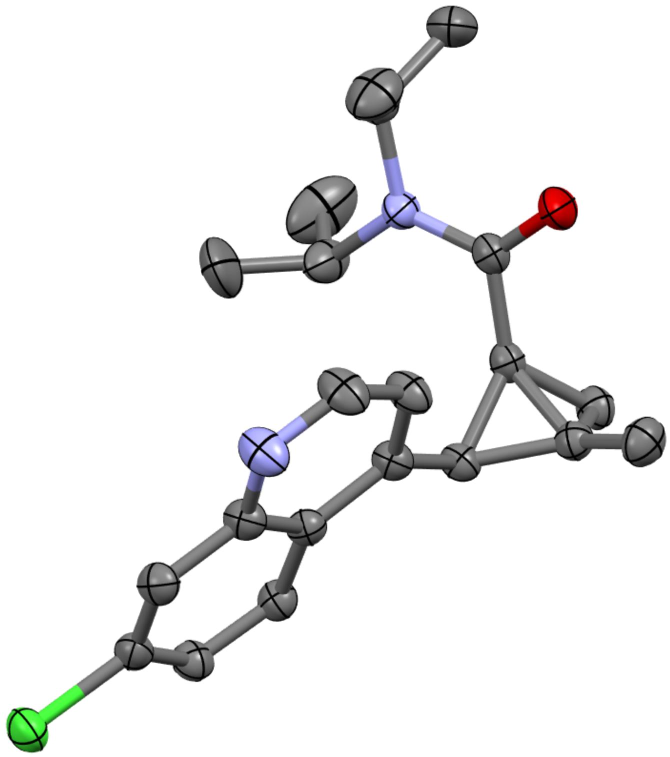

The structure of **3l** determined from X-ray diffraction studies; displacement ellipsoids are drawn at 50% probability and hydrogen atoms are omitted for clarity.

2. Copies of NMR spectra

(1*S**,3*S**)-*N*,*N*-diisopropyl-3-methyl-2-phenylbicyclo[1.1.0]butane-1-carboxamide, 3a

^{1}H NMR (400 MHz, Chloroform-*d*)

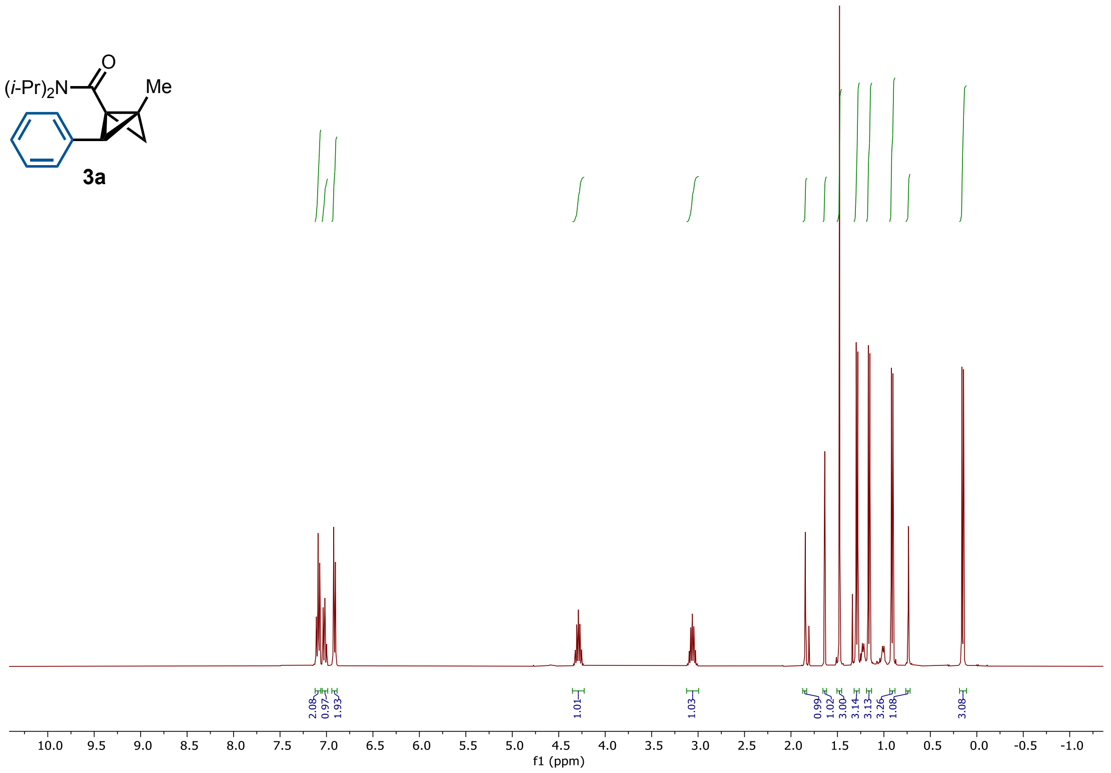

^{13}C NMR (101 MHz, Chloroform-*d*)

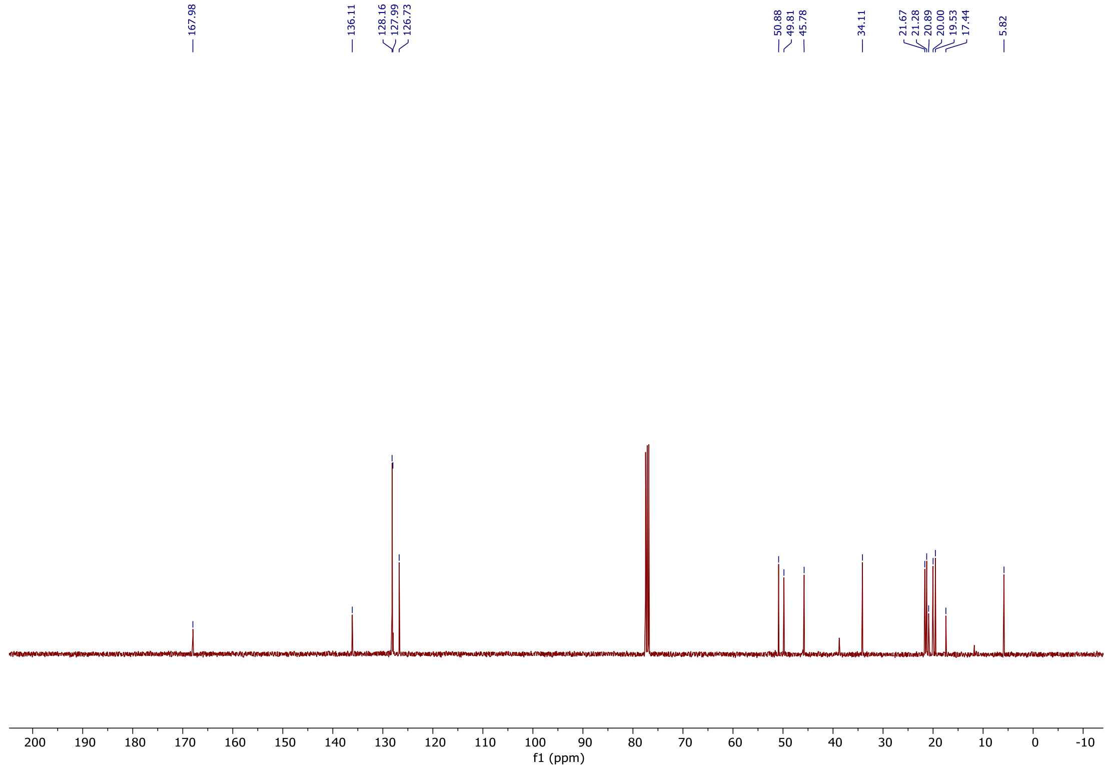

^{1}H COSY (400 MHz, Chloroform-*d*)

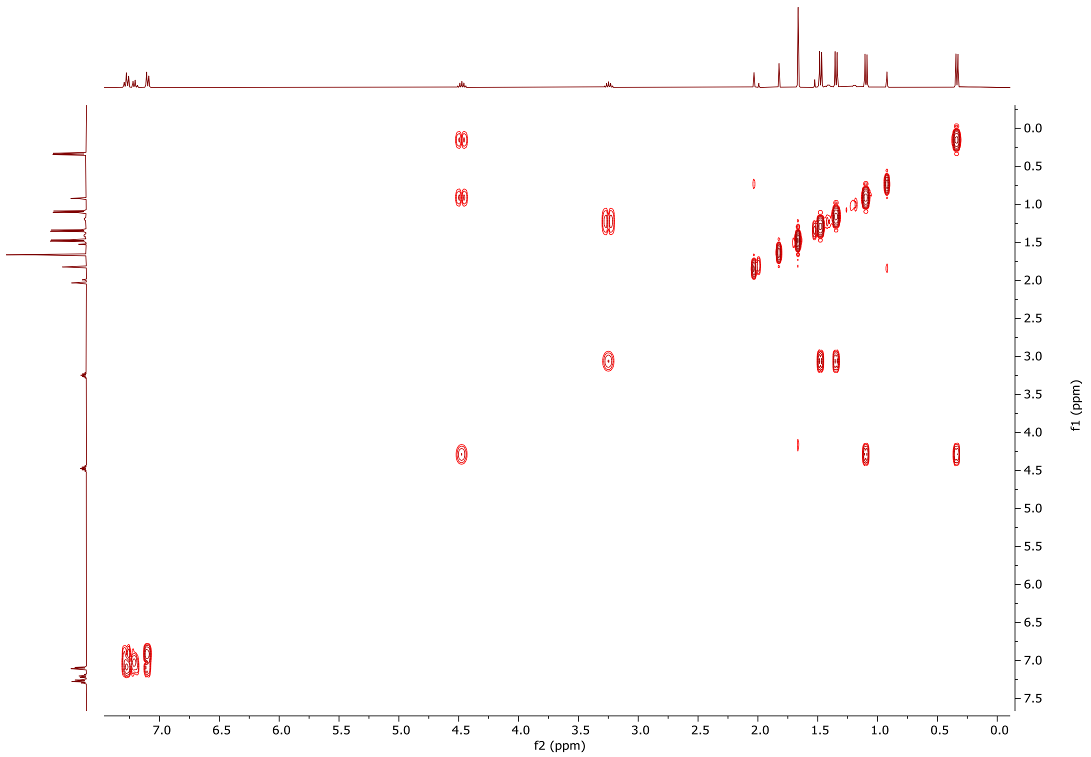

^{1}H/^{13}C HSQC (400/101 MHz, Chloroform-*d*)

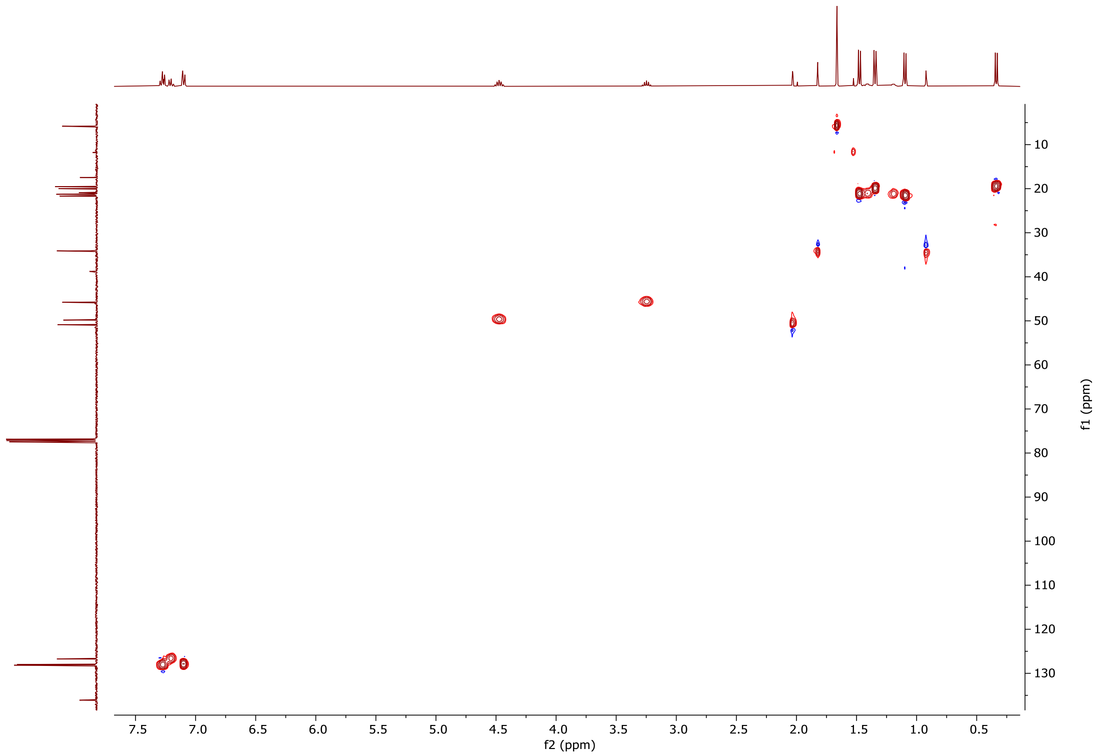

(1*S,3*S**)-*N*,*N*-diisopropyl-3-methyl-2-(4-nitrophenyl)bicyclo[1.1.0]butane-1-carboxamide, 3b**

^{1}H NMR (600 MHz, Chloroform-*d*)

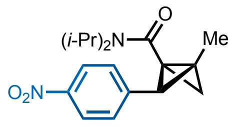

3b

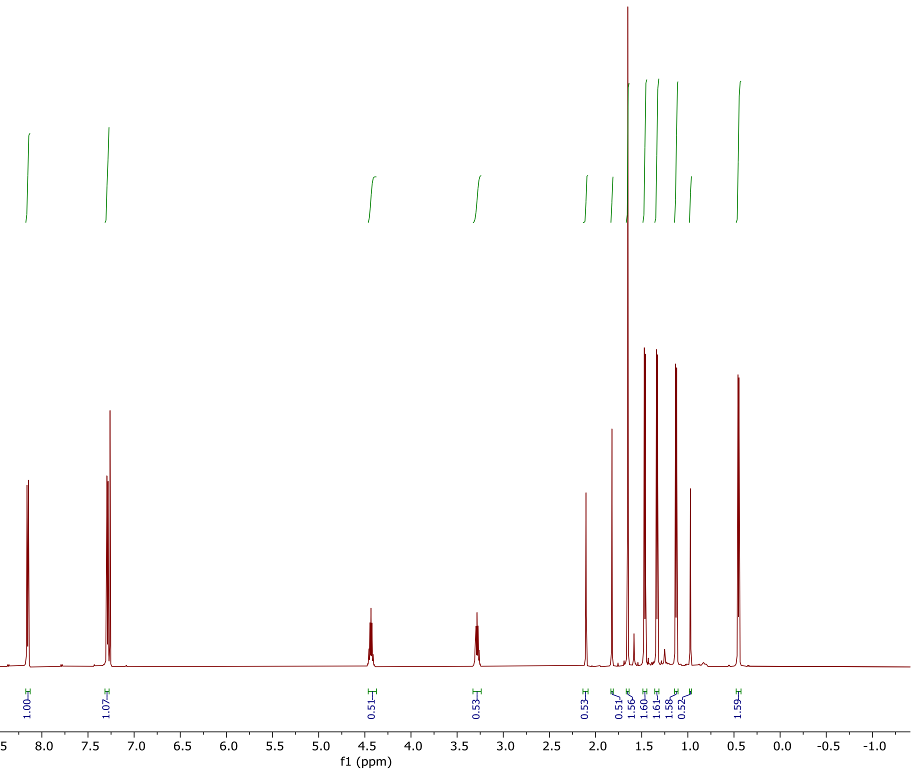

^{13}C NMR (151 MHz, Chloroform-*d*)

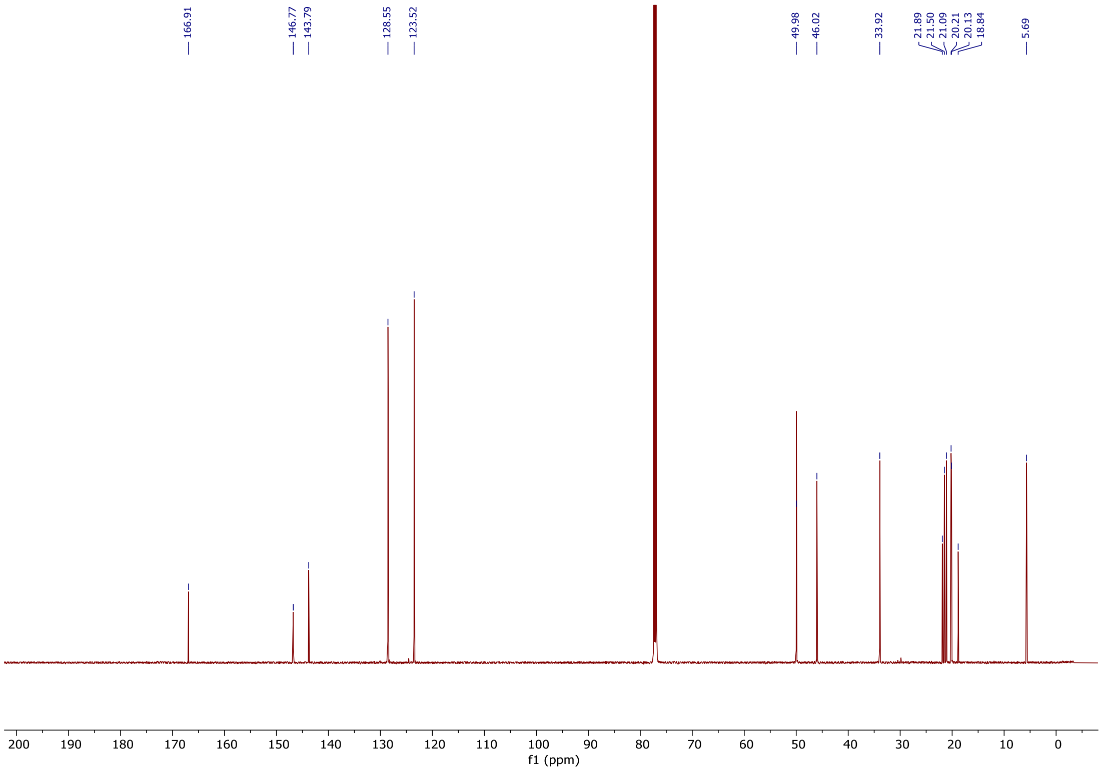

¹H COSY (600 MHz, Chloroform-*d*)

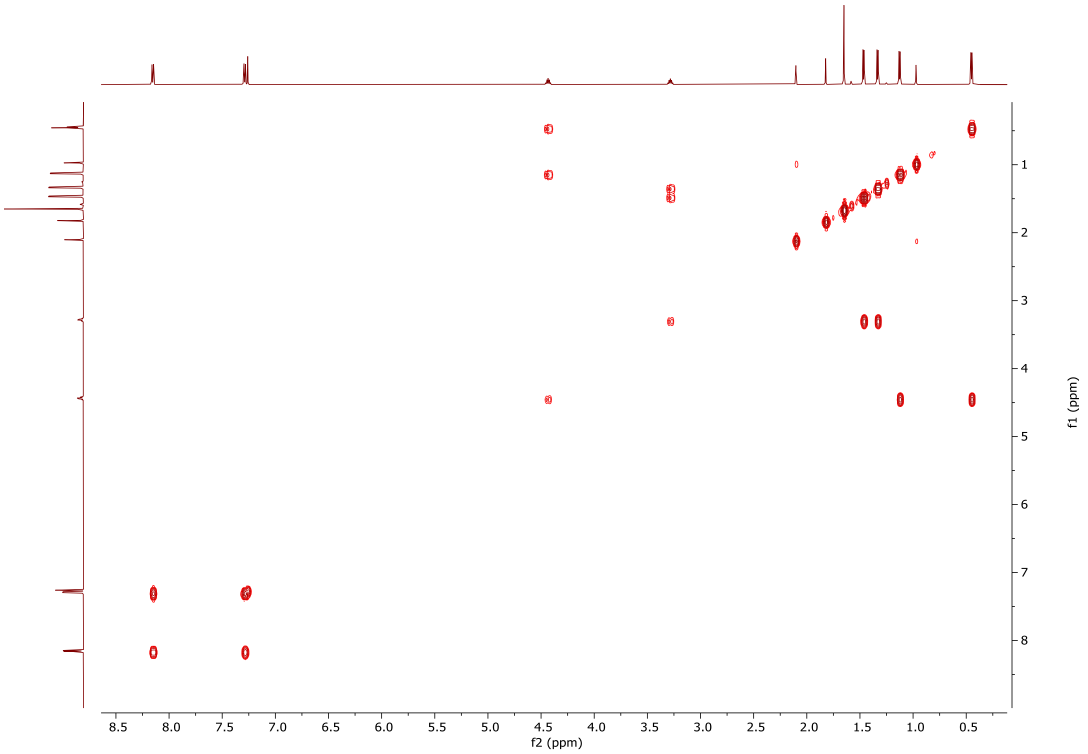

¹H/¹³C HSQC (600/151 MHz, Chloroform-*d*)

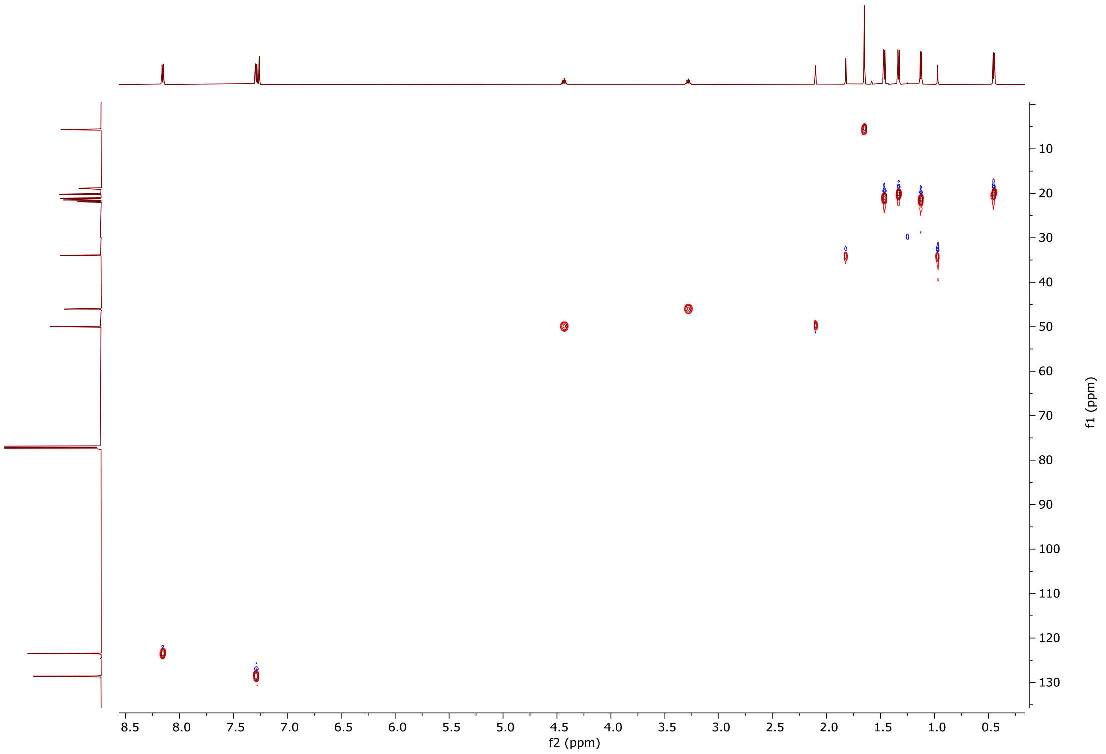

$^{1}H/^{13}C$ HMBC (600/151 MHz, Chloroform-*d*)

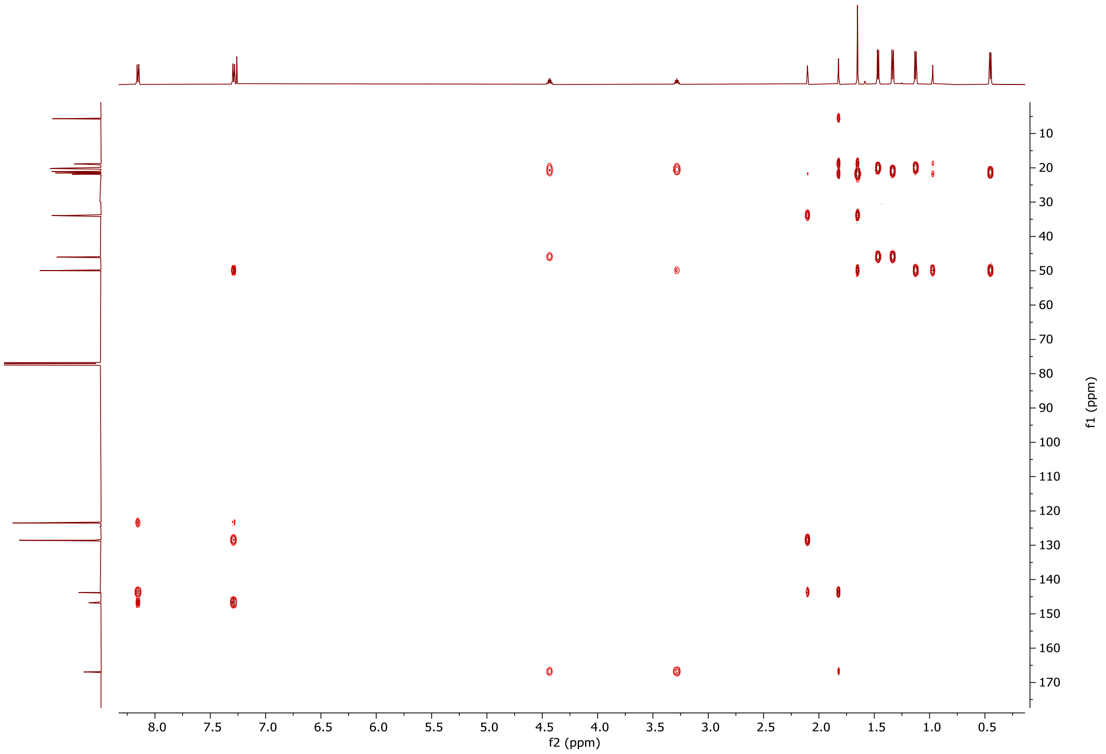

^{1}H NOSEY (600 MHz, Chloroform-*d*)

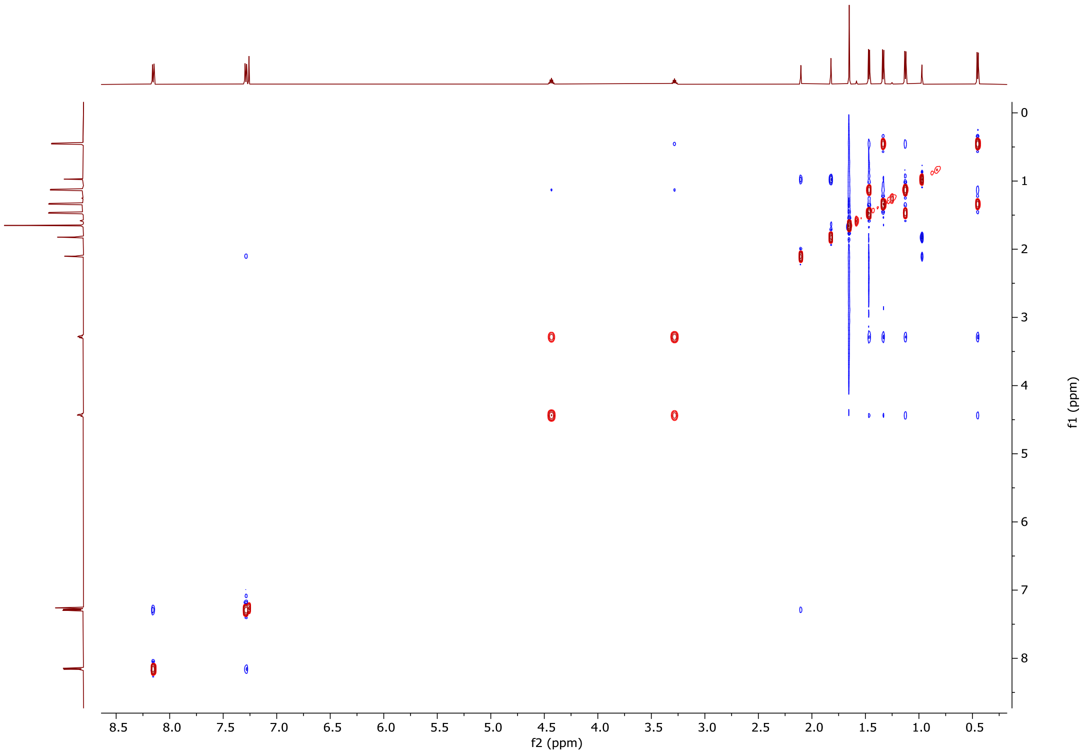

(1*S,3*S**)-*N*,*N*-diisopropyl-3-methyl-2-(4-(trifluoromethyl)phenyl)bicyclo[1.1.0]butane-1-carboxamide, 3c**

^{1}H NMR (600 MHz, Chloroform-*d*)

3c

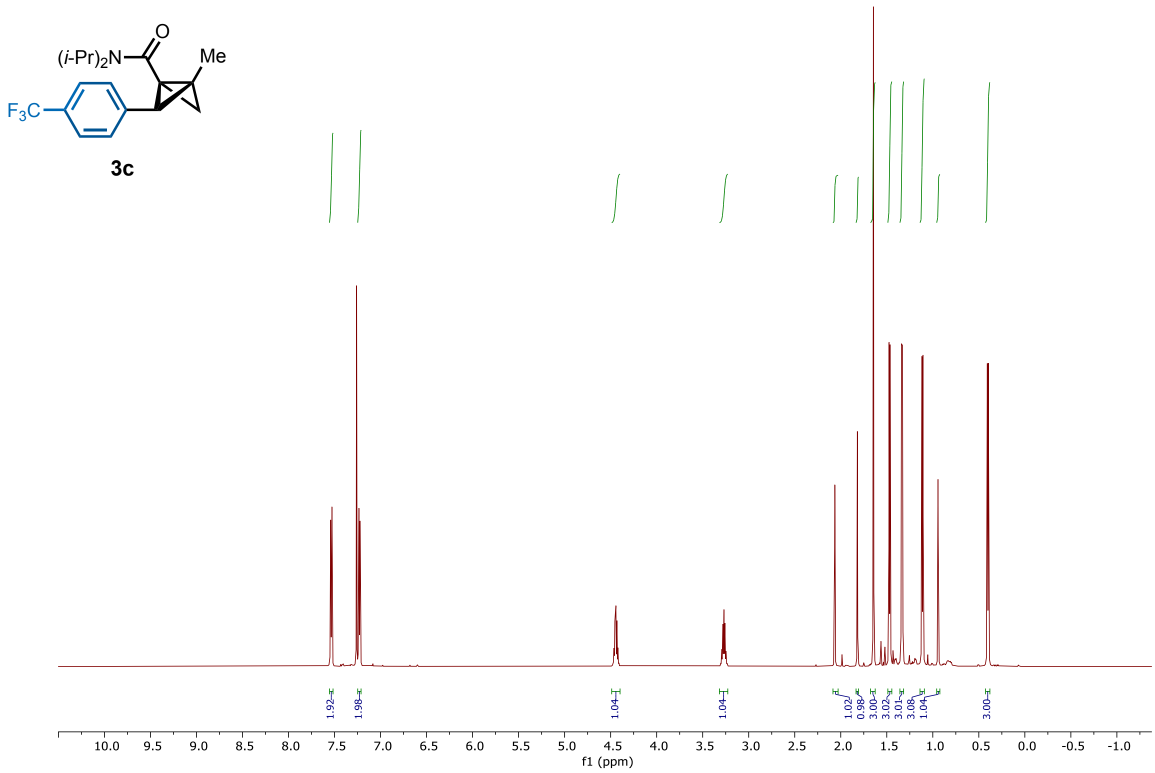

^{13}C NMR (151 MHz, Chloroform-*d*)

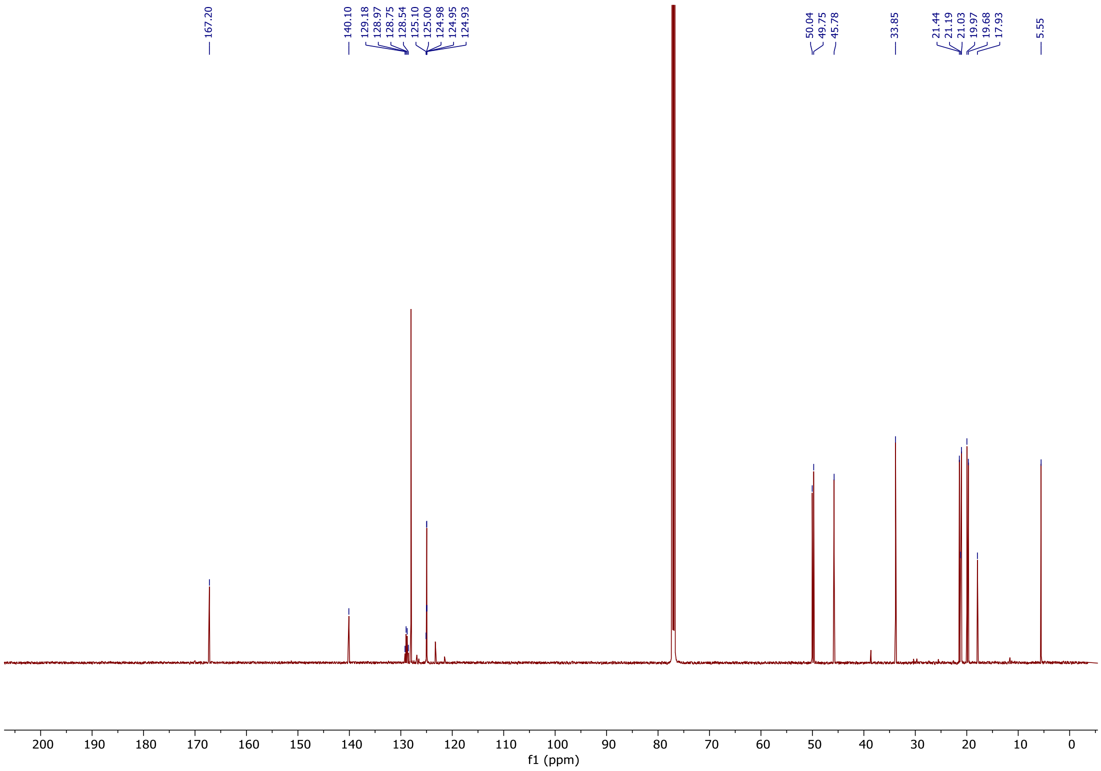

^{1}H COSY (600 MHz, Chloroform-*d*)

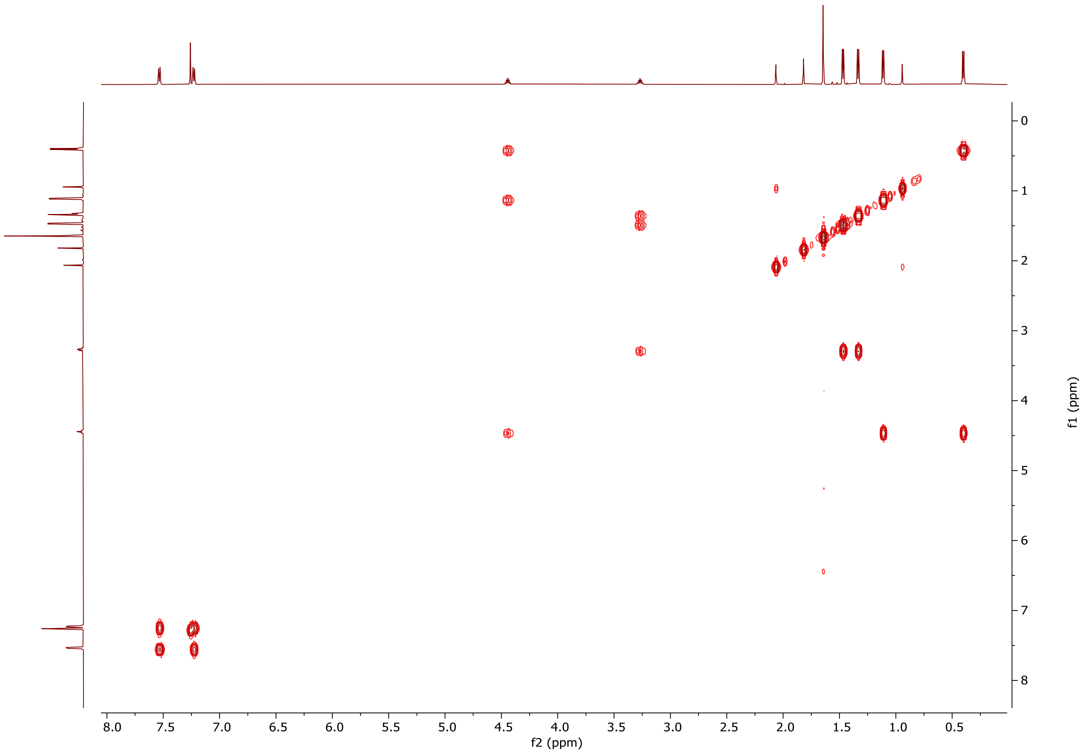

^{1}H/^{13}C HSQC (600/151 MHz, Chloroform-*d*)

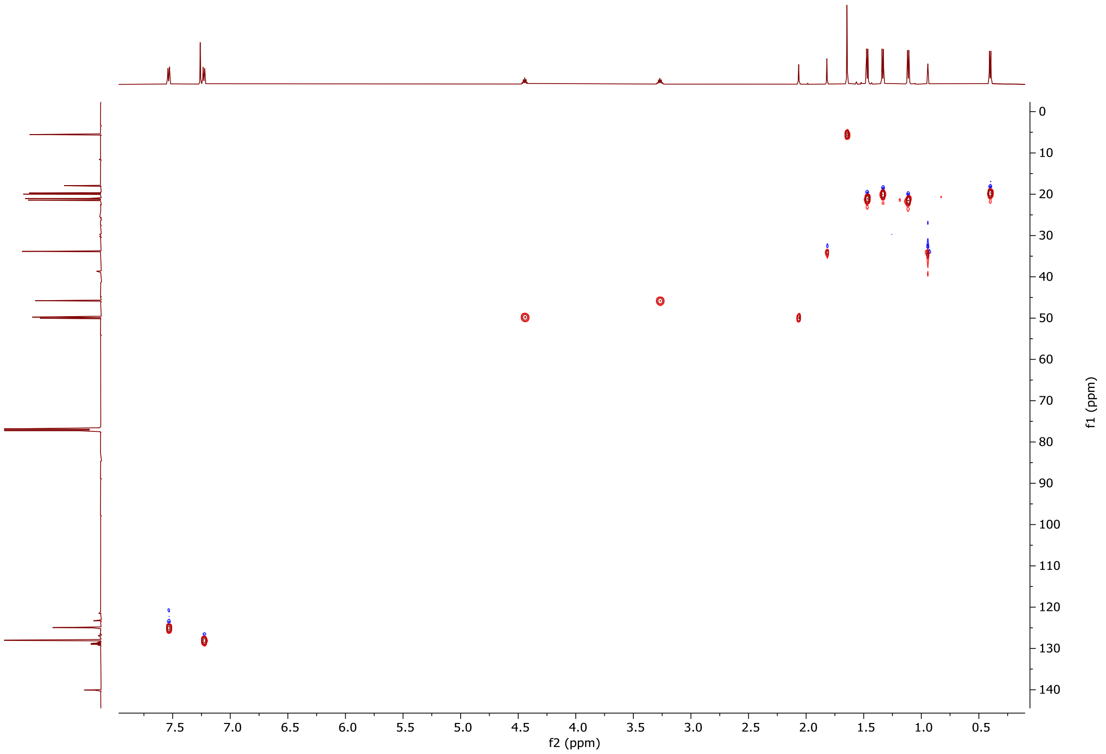

$^{1}H/^{13}C$ **HMBC** (600/151 MHz, Chloroform-*d*)

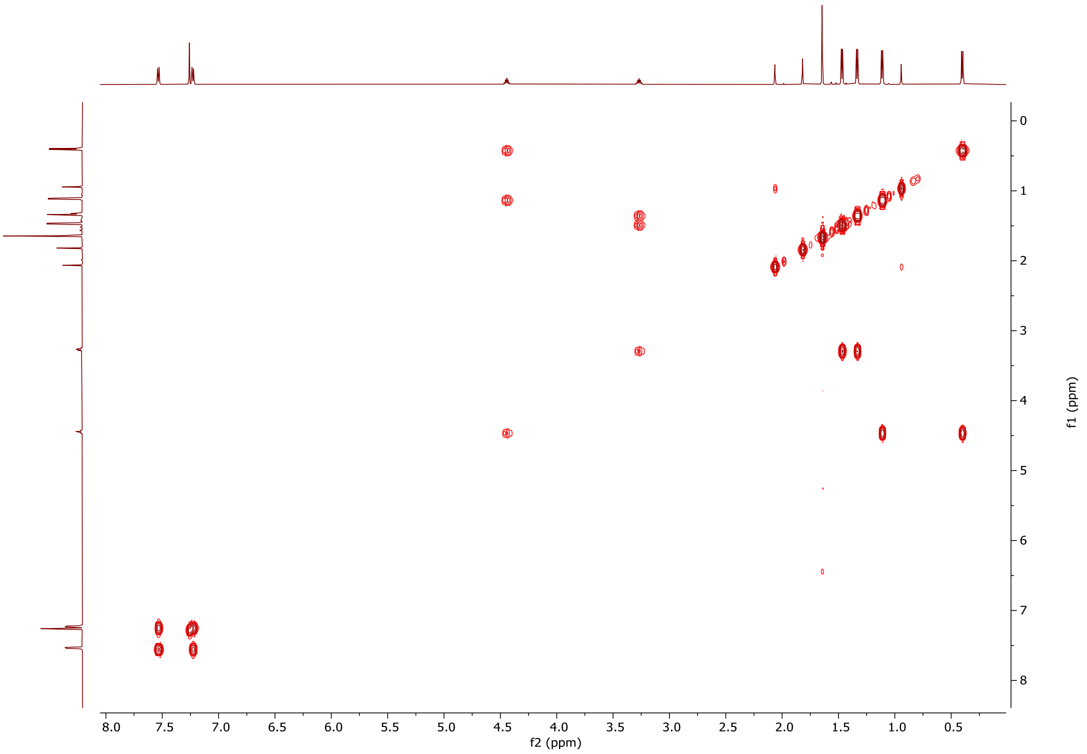

^{19}F **NMR** (471 MHz, Chloroform-*d*)

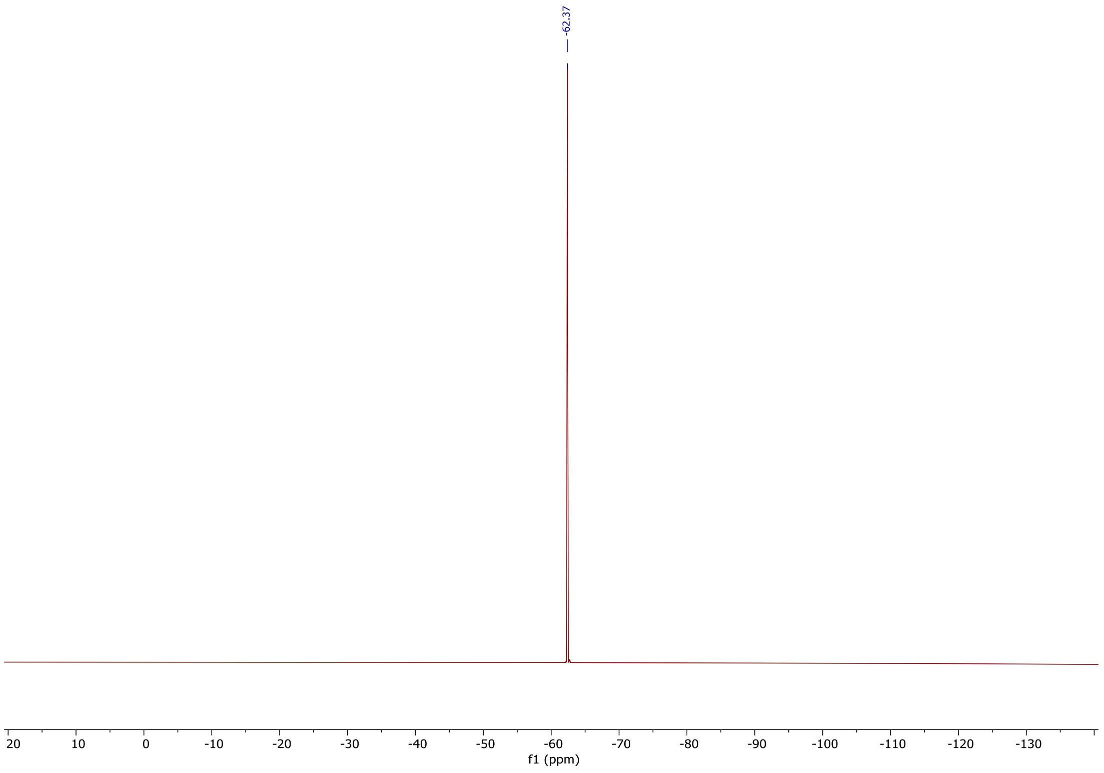

^{1}H NOSEY (500 MHz, Chloroform-*d*)

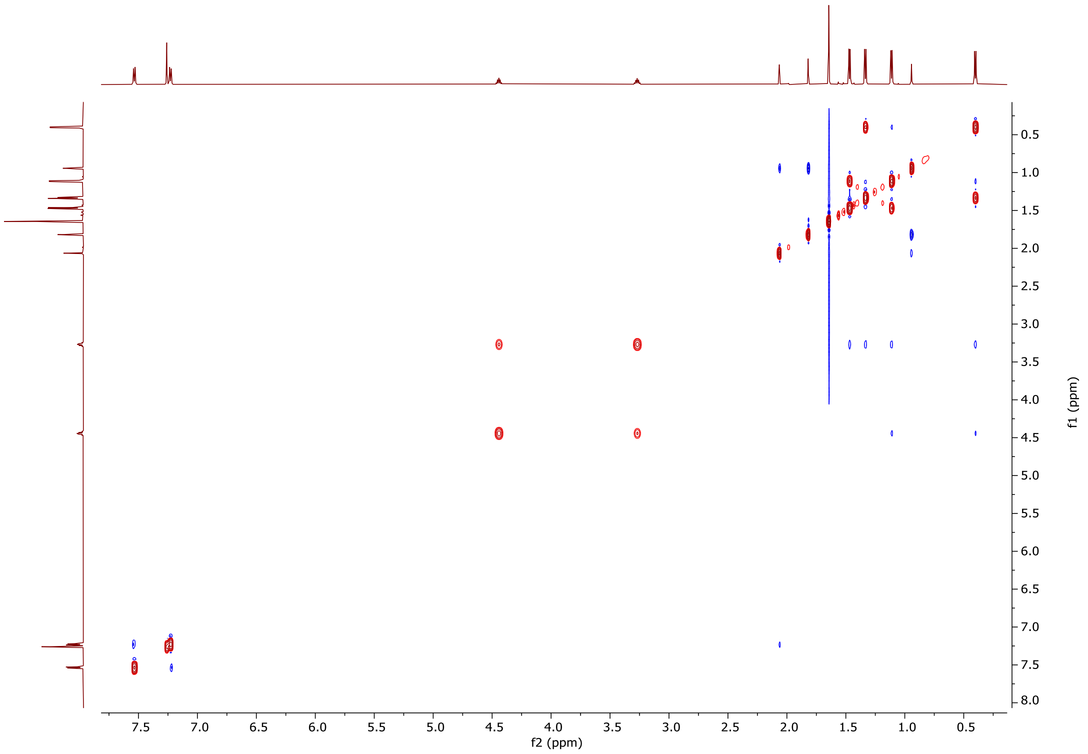

(1*S,3*S**)-2-(4-chlorophenyl)-*N*,*N*-diisopropyl-3-methylbicyclo[1.1.0]butane-1-carboxamide, 3d**

^{1}H NMR (600 MHz, Chloroform-*d*)

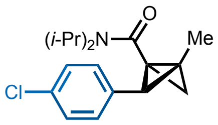

3d

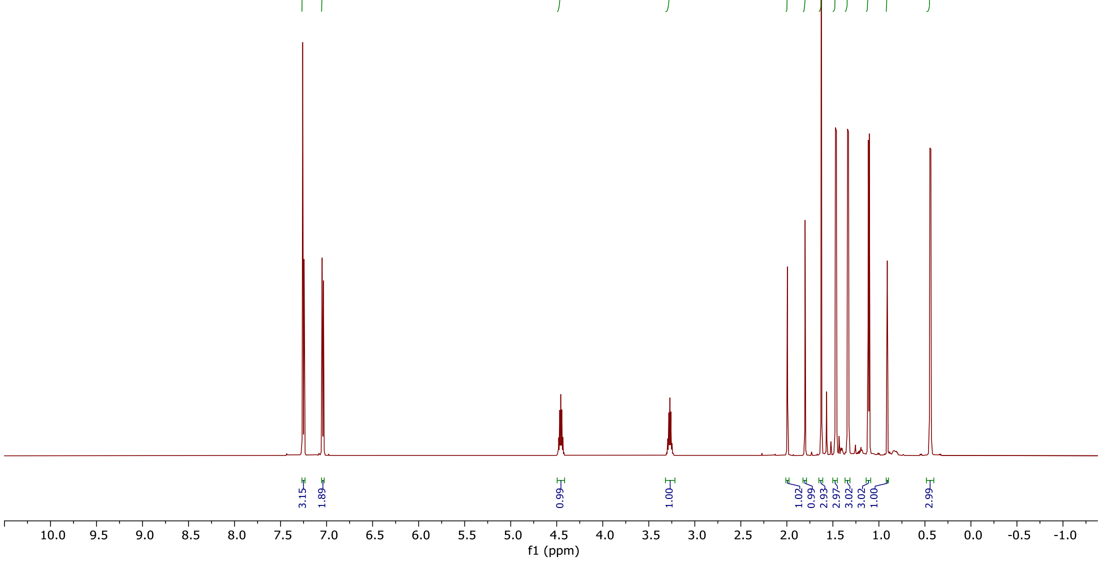

^{13}C NMR (151 MHz, Chloroform-*d*)

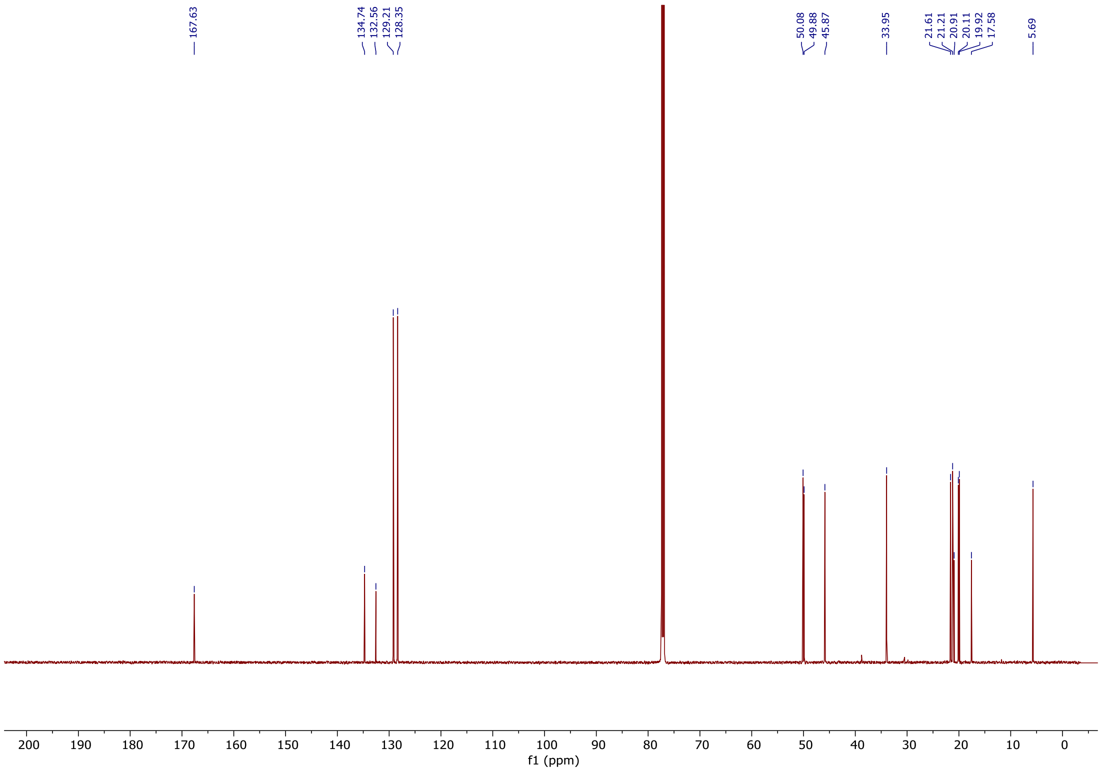

^{1}H COSY (600 MHz, Chloroform-*d*)

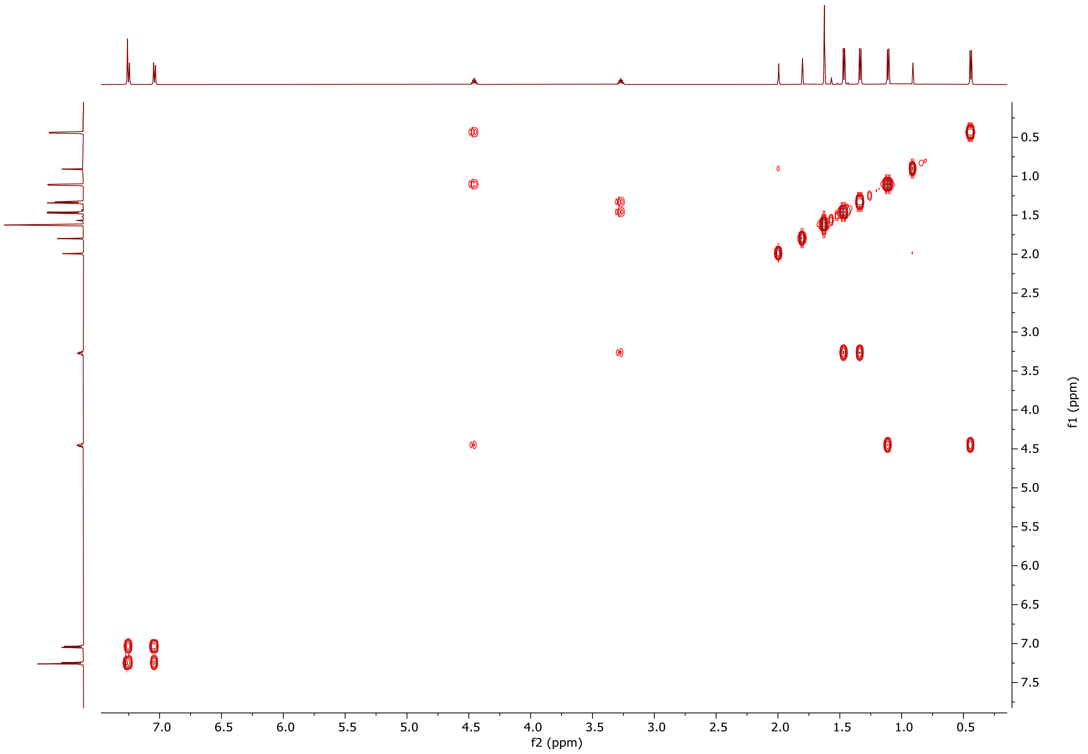

^{1}H/^{13}C HSQC (600/151 MHz, Chloroform-*d*)

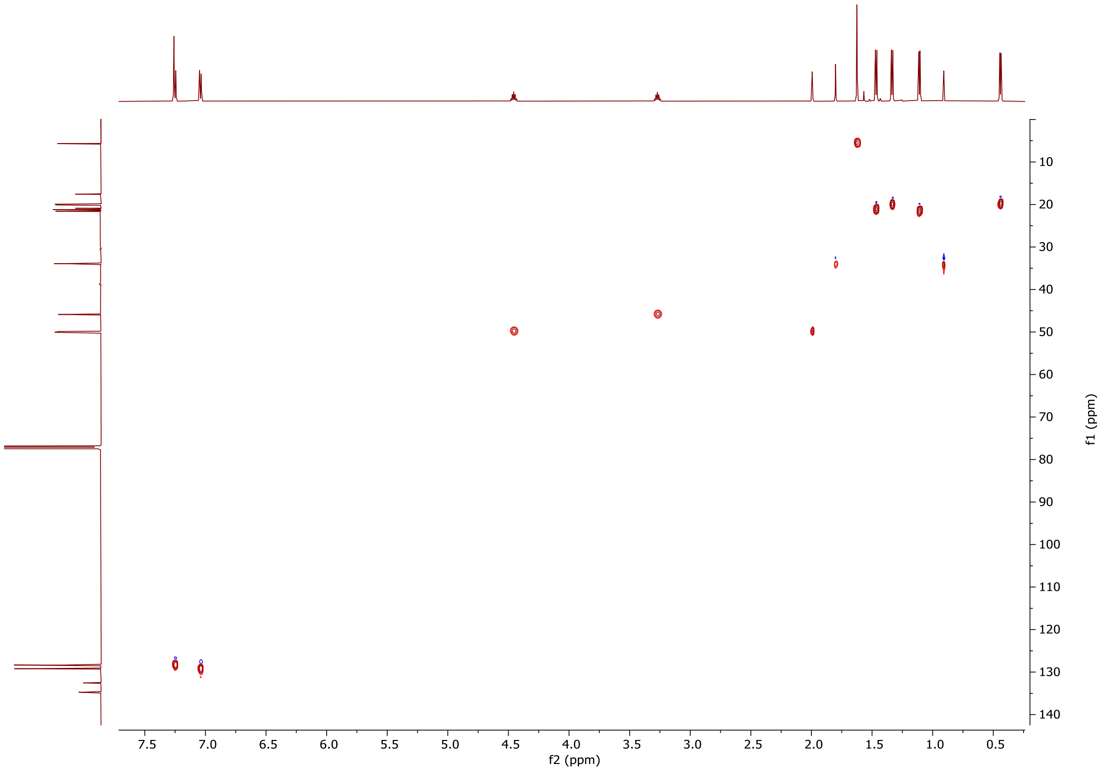

$^{1}H/^{13}C$ HMBC (600/151 MHz, Chloroform-*d*)

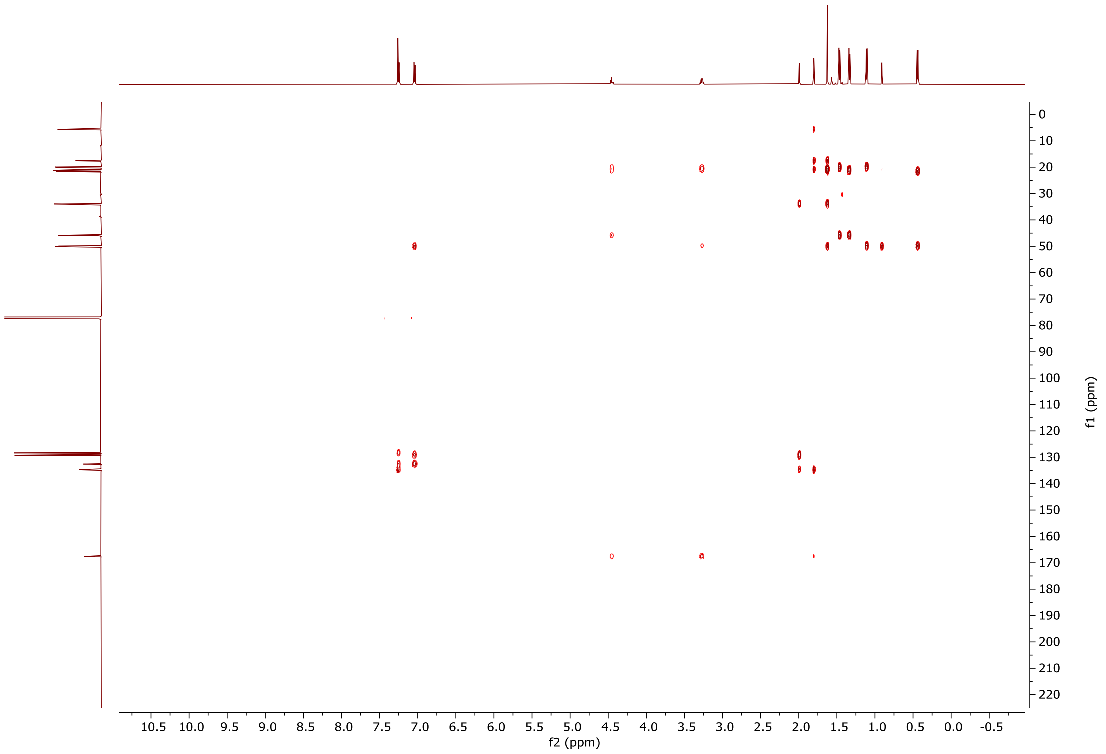

^{1}H NOSEY (600 MHz, Chloroform-*d*)

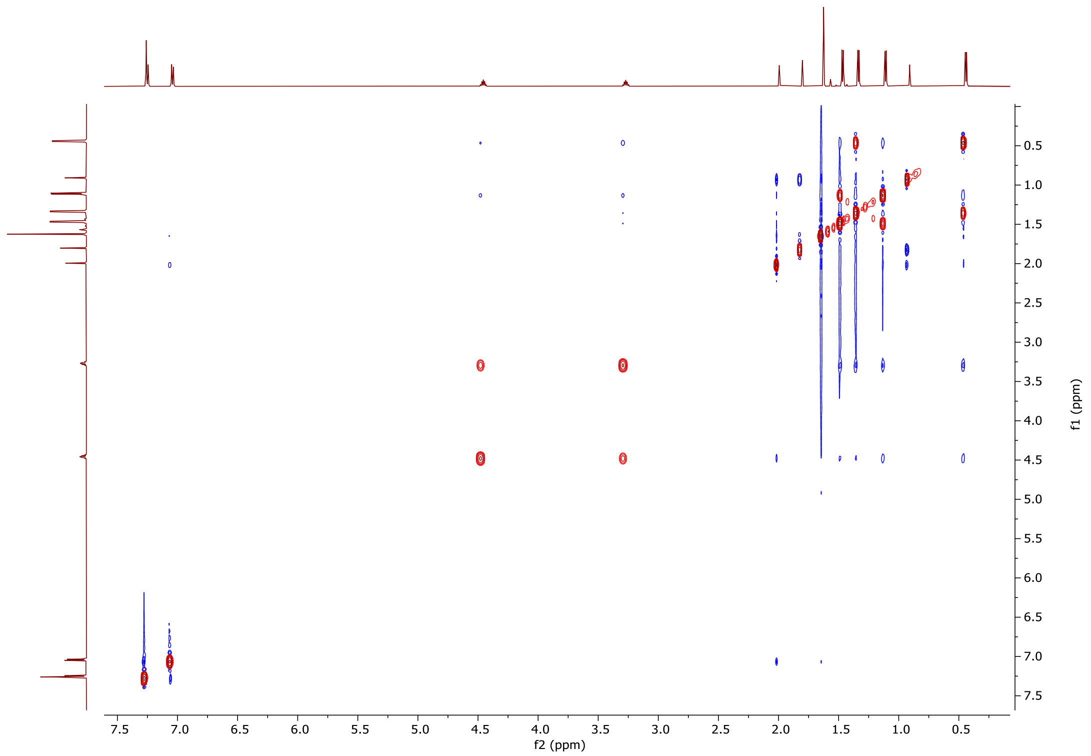

(1*S,3*S**)-*N*,*N*-diisopropyl-2-(4-methoxyphenyl)-3-methylbicyclo[1.1.0]butane-1-carboxamide, 3e**

^{1}H NMR (600 MHz, Chloroform-*d*)

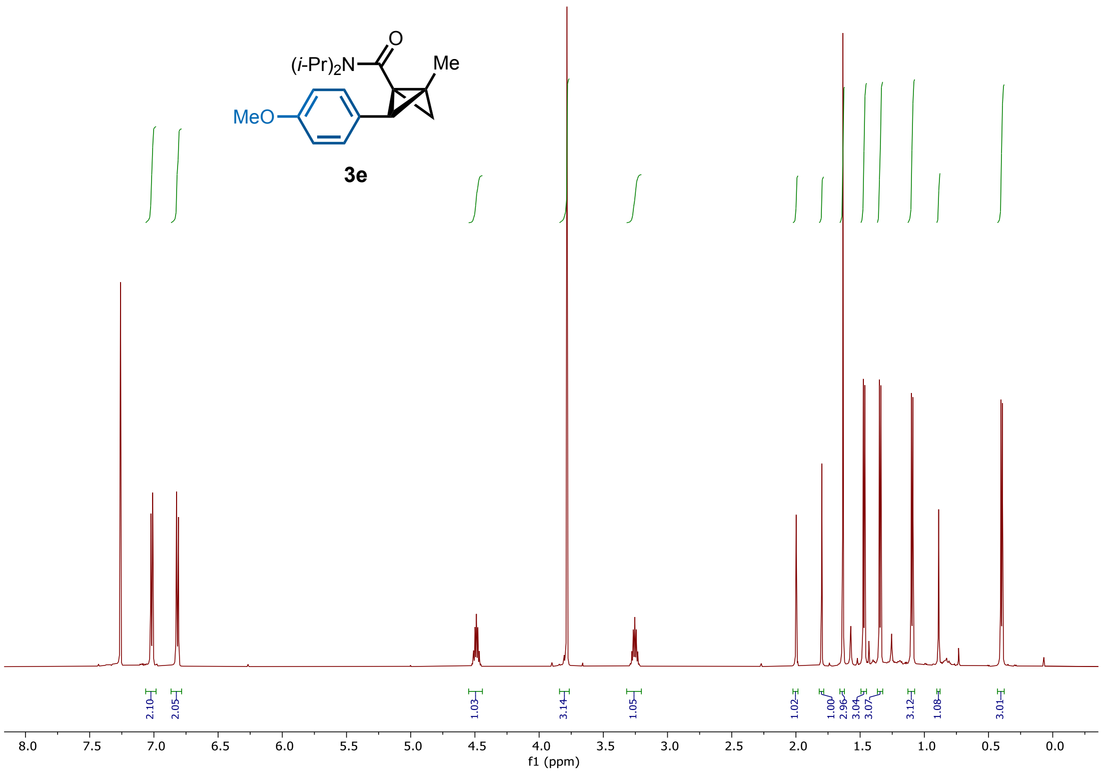

^{13}C NMR (151 MHz, Chloroform-*d*)

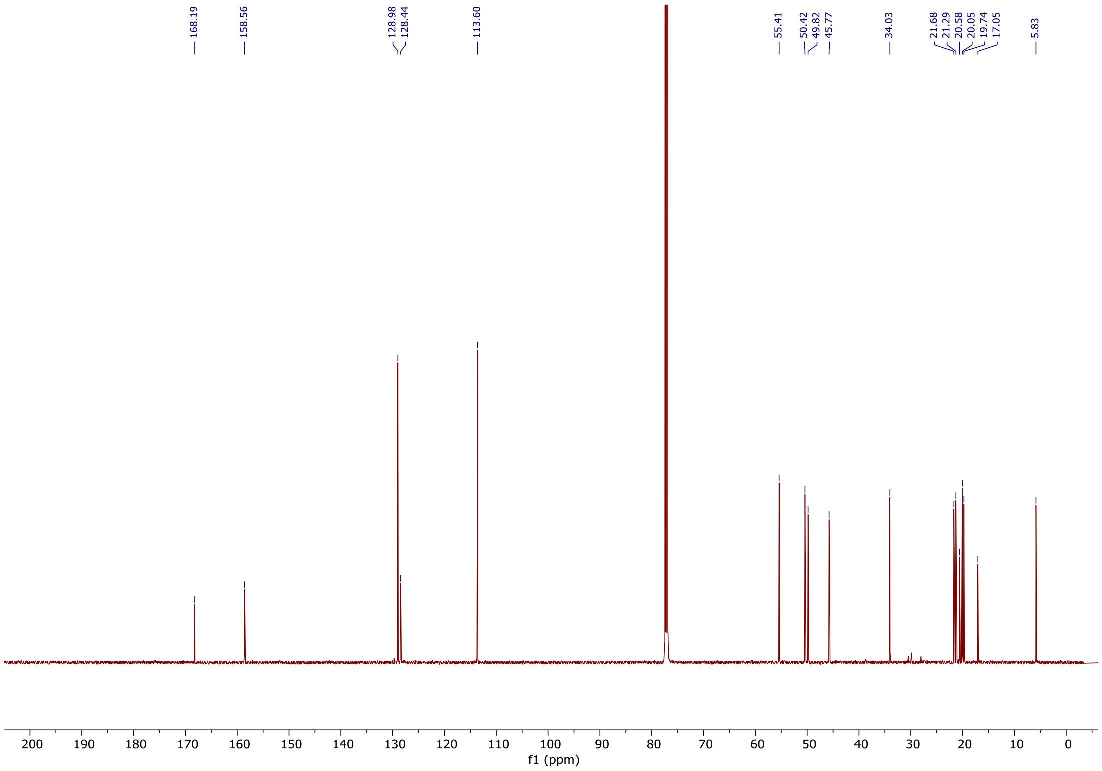

^{1}H COSY (600 MHz, Chloroform-*d*)

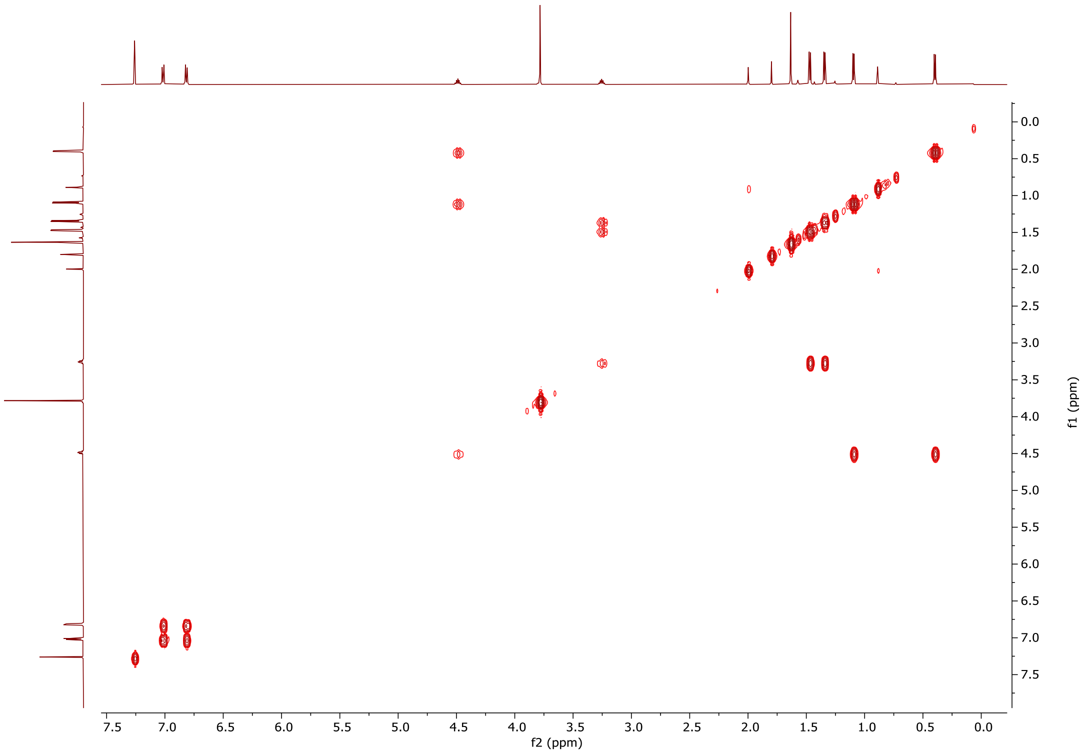

^{1}H/^{13}C HSQC (600/151 MHz, Chloroform-*d*)

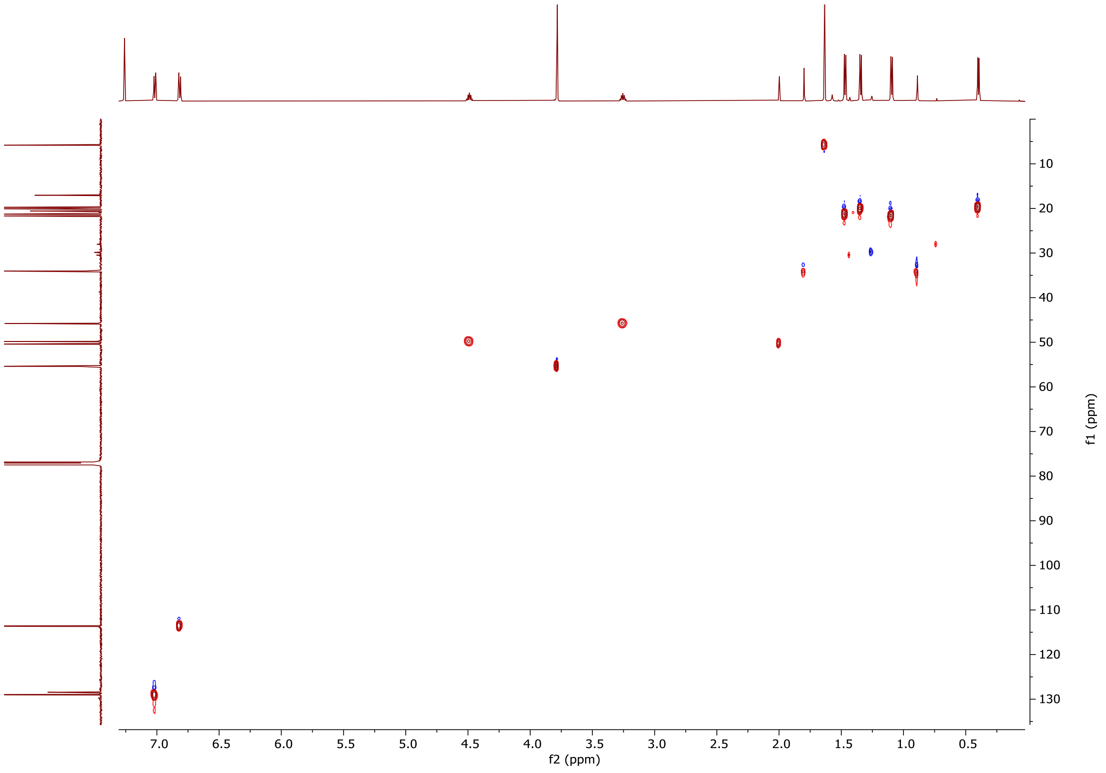

$^1H/^{13}C$ HMBC (600/151 MHz, Chloroform-*d*)

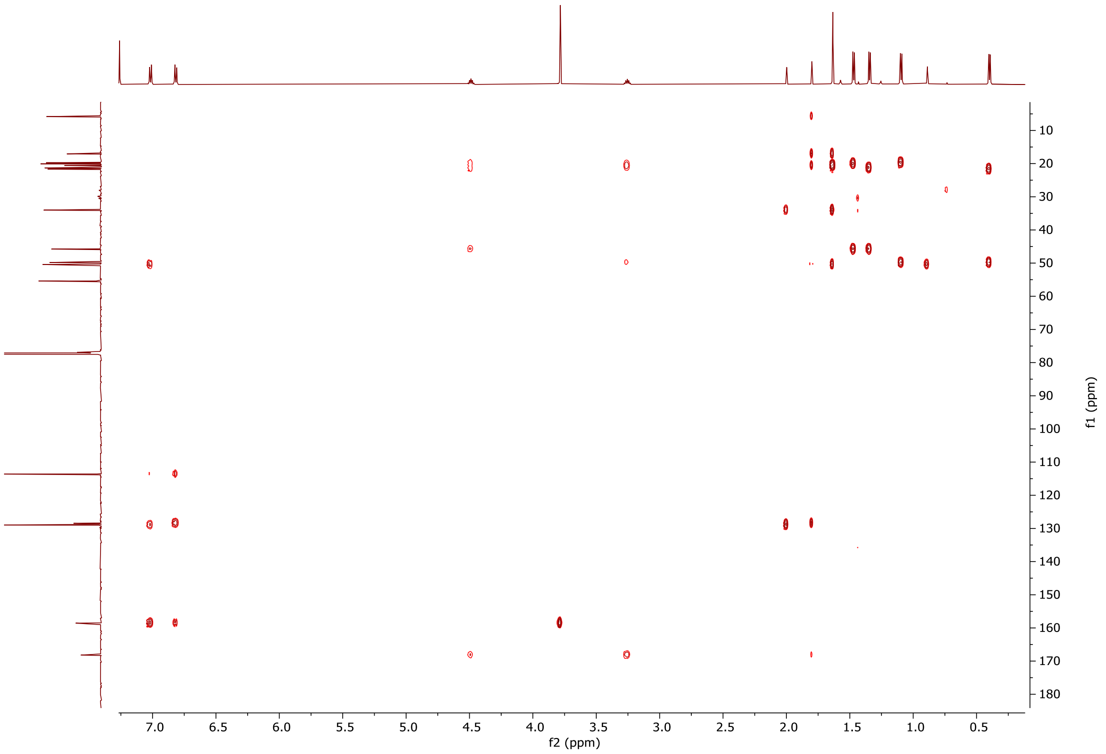

^1H NOSEY (600 MHz, Chloroform-*d*)

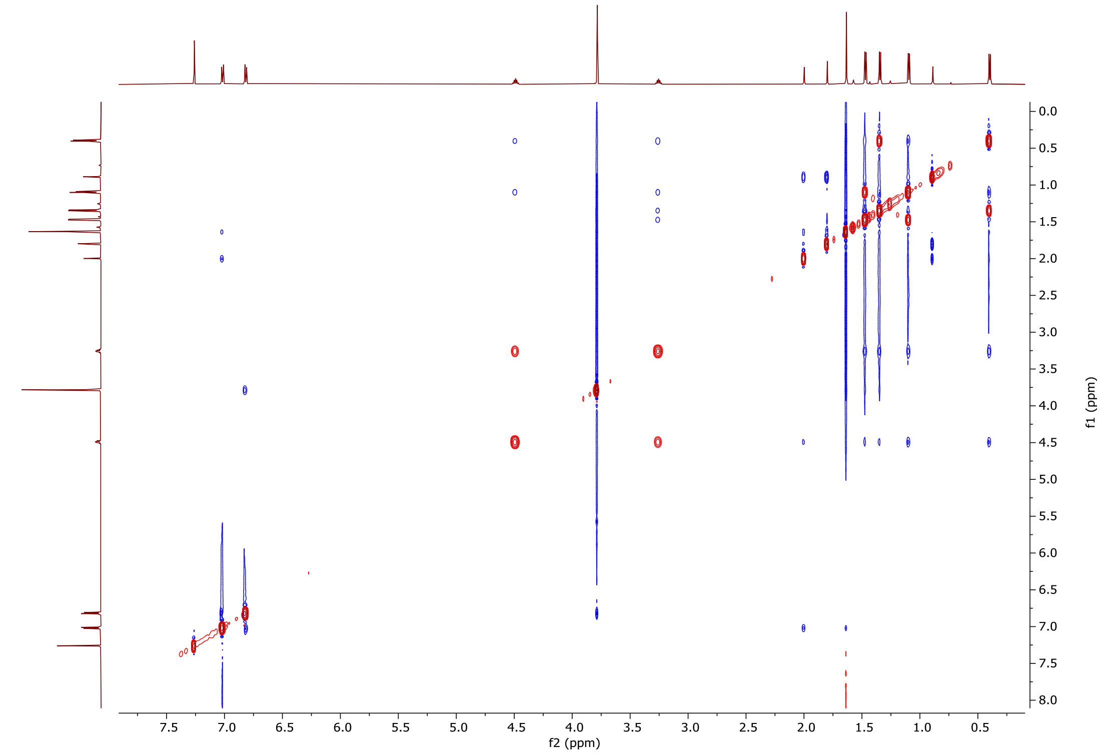

(1*S,3*S**)-2-(2-cyanophenyl)-*N*,*N*-diisopropyl-3-methylbicyclo[1.1.0]butane-1-carboxamide, 3f**

^{1}H NMR (600 MHz, Chloroform-*d*)

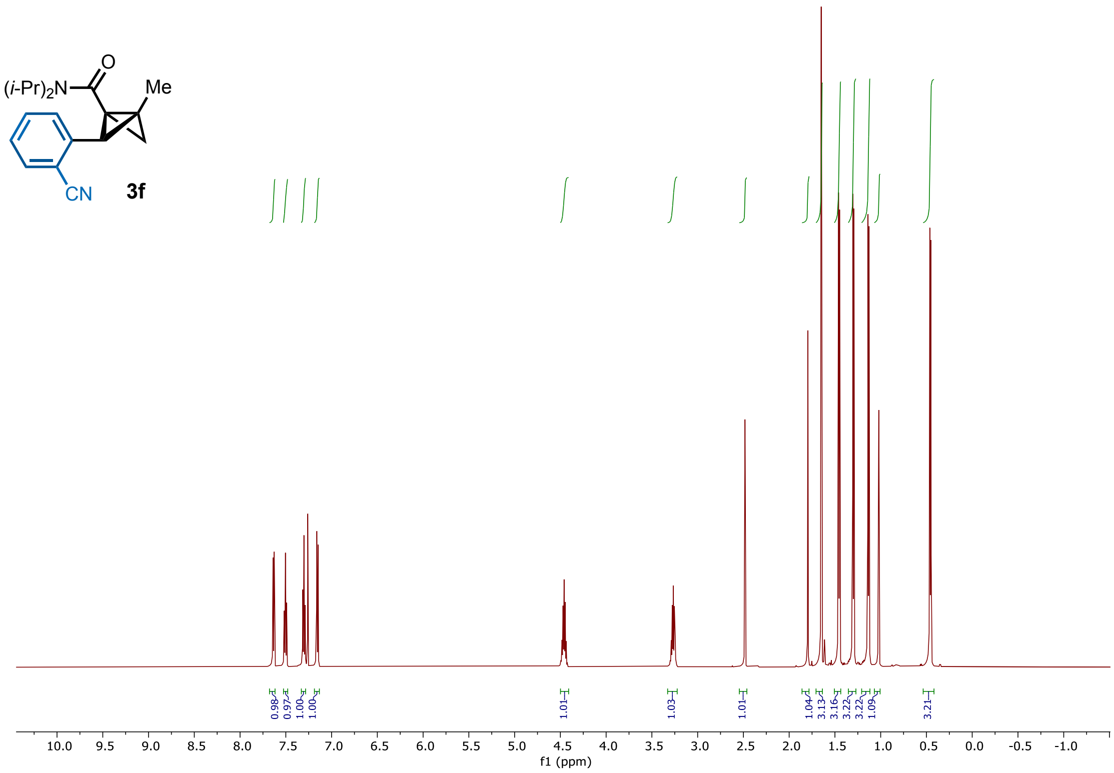

^{13}C NMR (151 MHz, Chloroform-*d*)

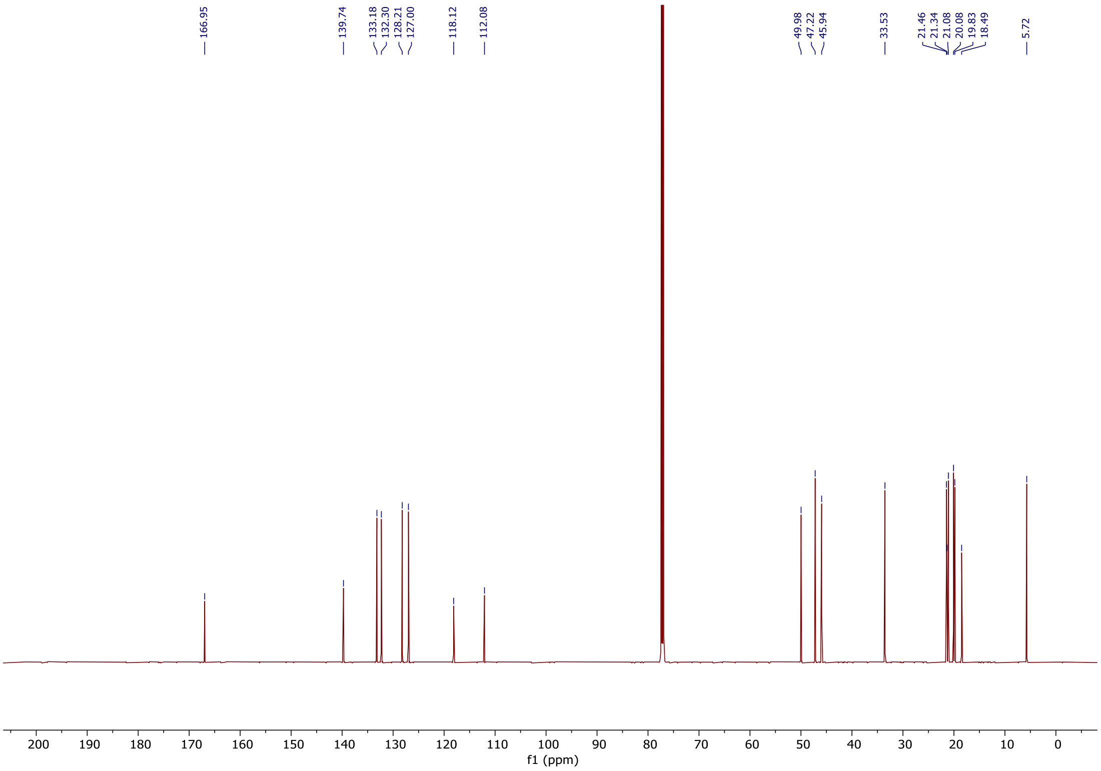

^{1}H COSY (600 MHz, Chloroform-*d*)

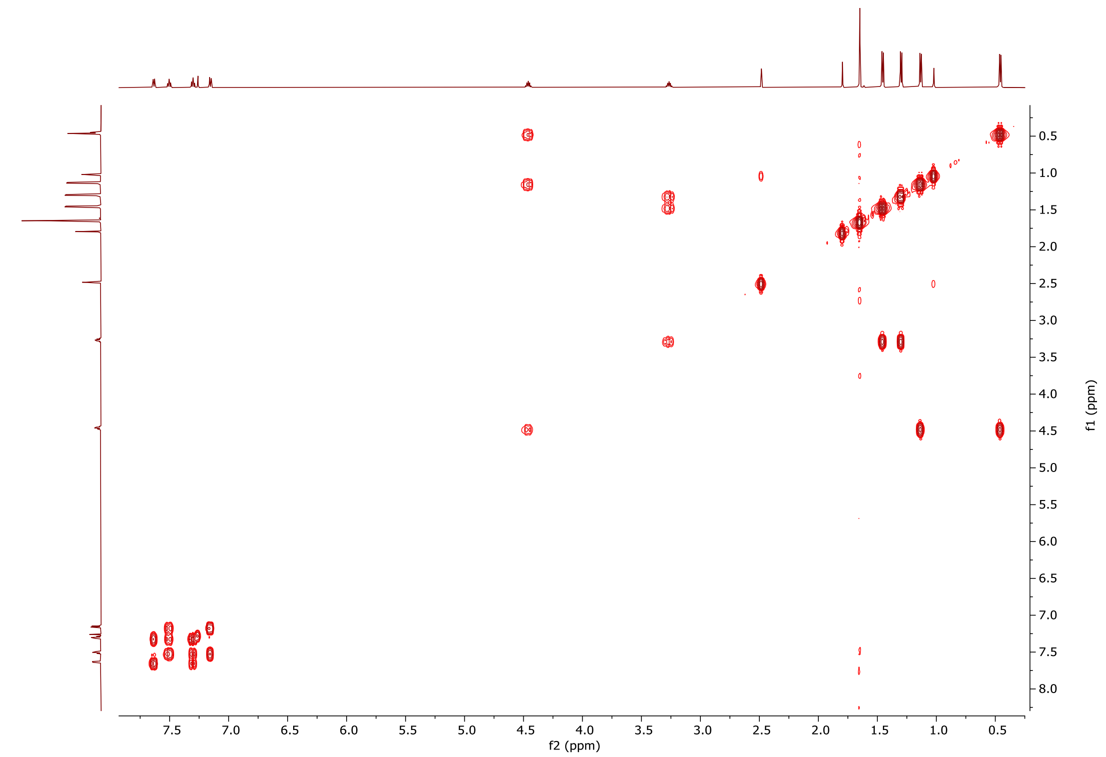

^{1}H/^{13}C HSQC (600/151 MHz, Chloroform-*d*)

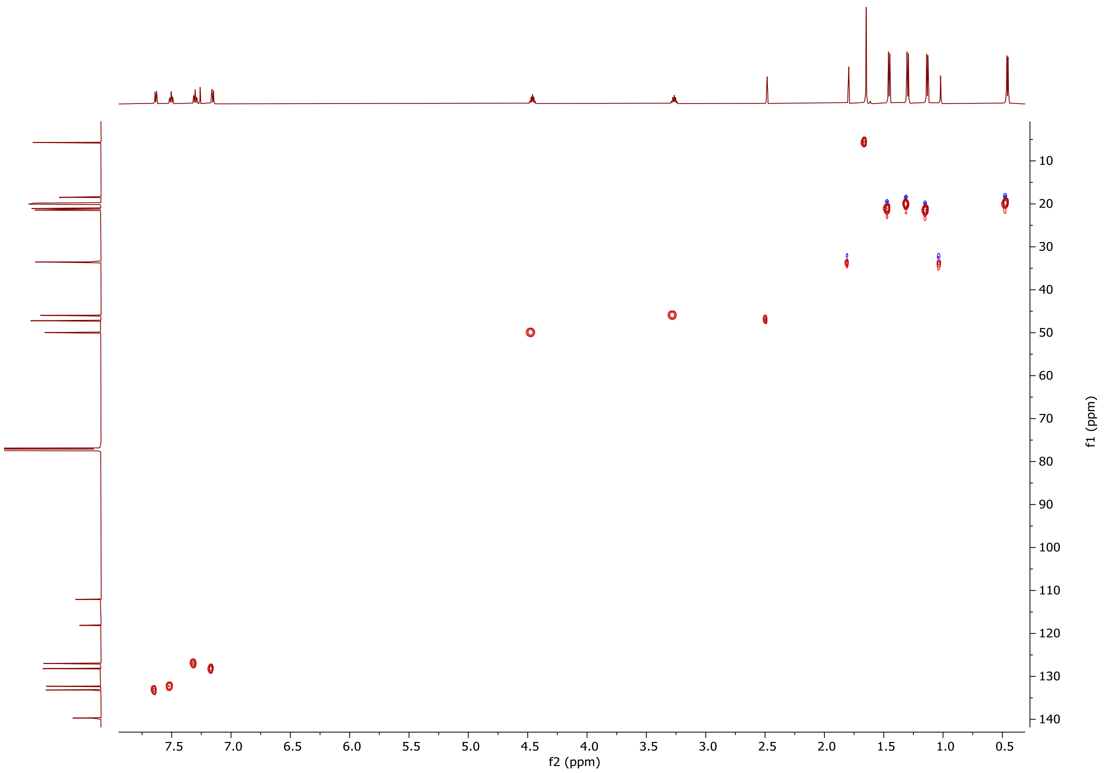

$^1H/^{13}C$ HMBC (600/151 MHz, Chloroform-*d*)

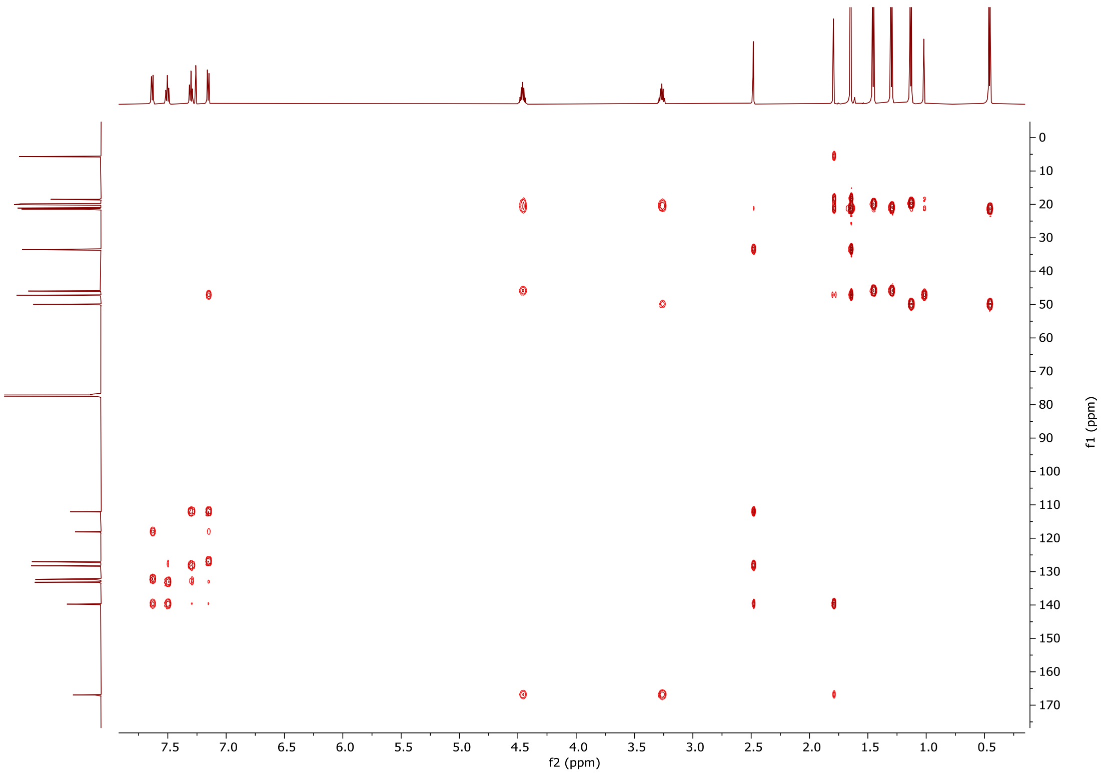

^1H NOSEY (600 MHz, Chloroform-*d*)

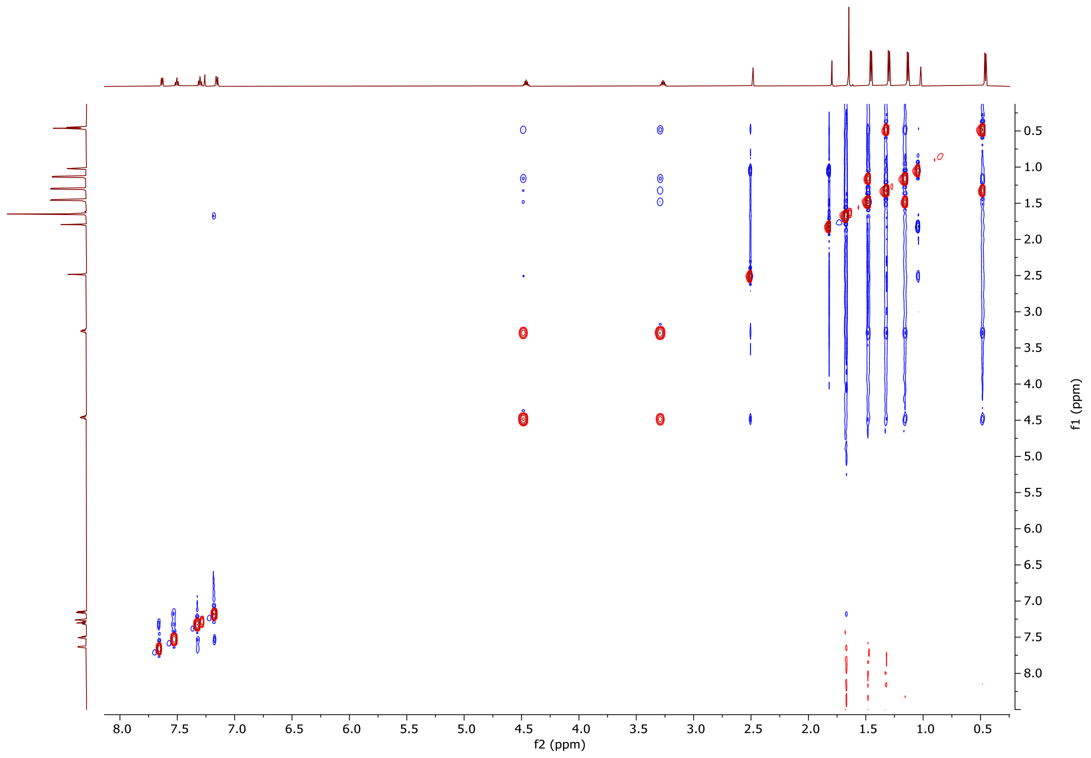

(1*S,3*S**)-*N*,*N*-diisopropyl-3-methyl-2-(o-tolyl)bicyclo[1.1.0]butane-1-carboxamide, 3g**

^{1}H NMR (600 MHz, Chloroform-*d*)

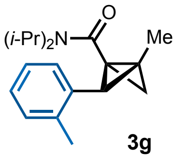

3g

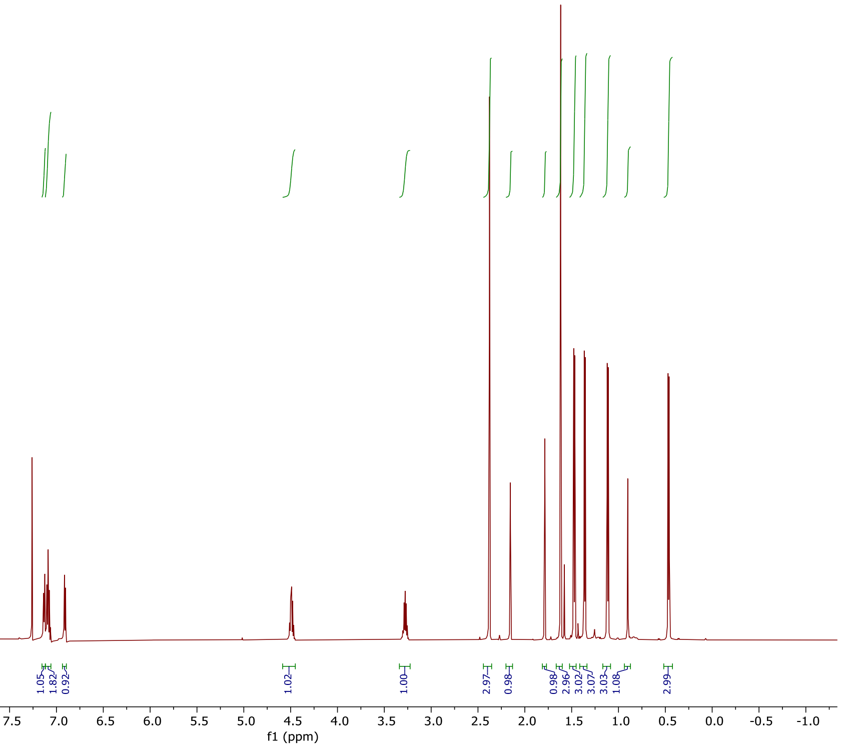

^{13}C NMR (151 MHz, Chloroform-*d*)

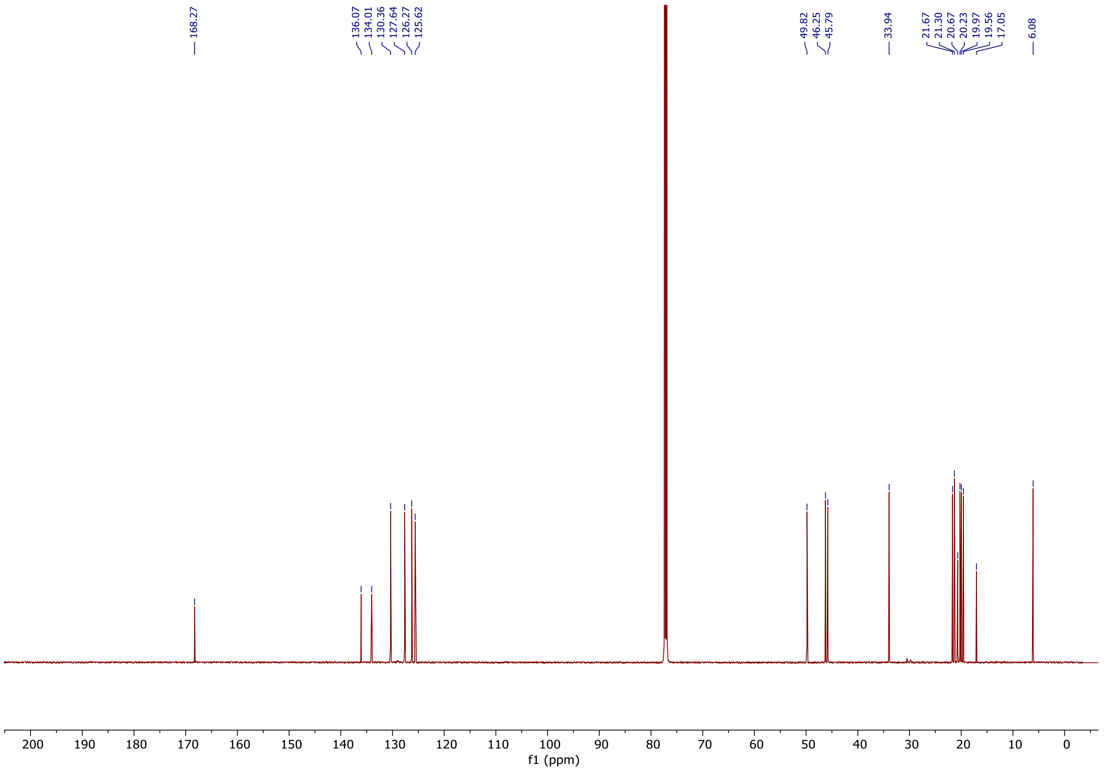

^{1}H COSY (600 MHz, Chloroform-*d*)

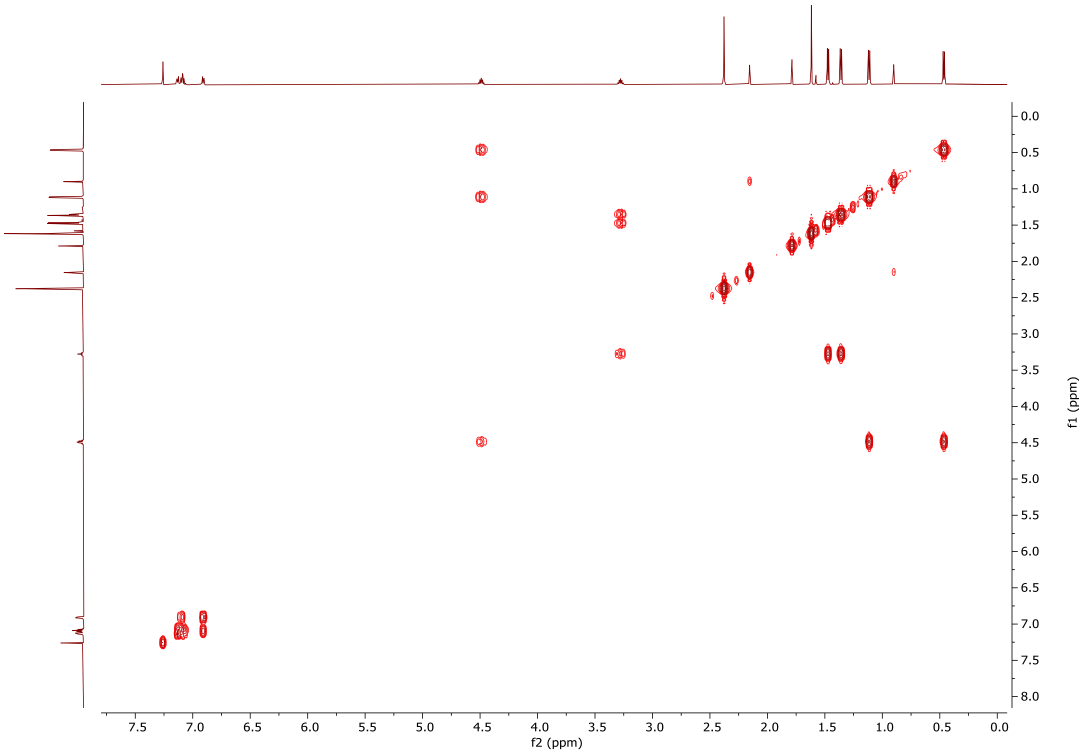

^{1}H/^{13}C HSQC (600/151 MHz, Chloroform-*d*)

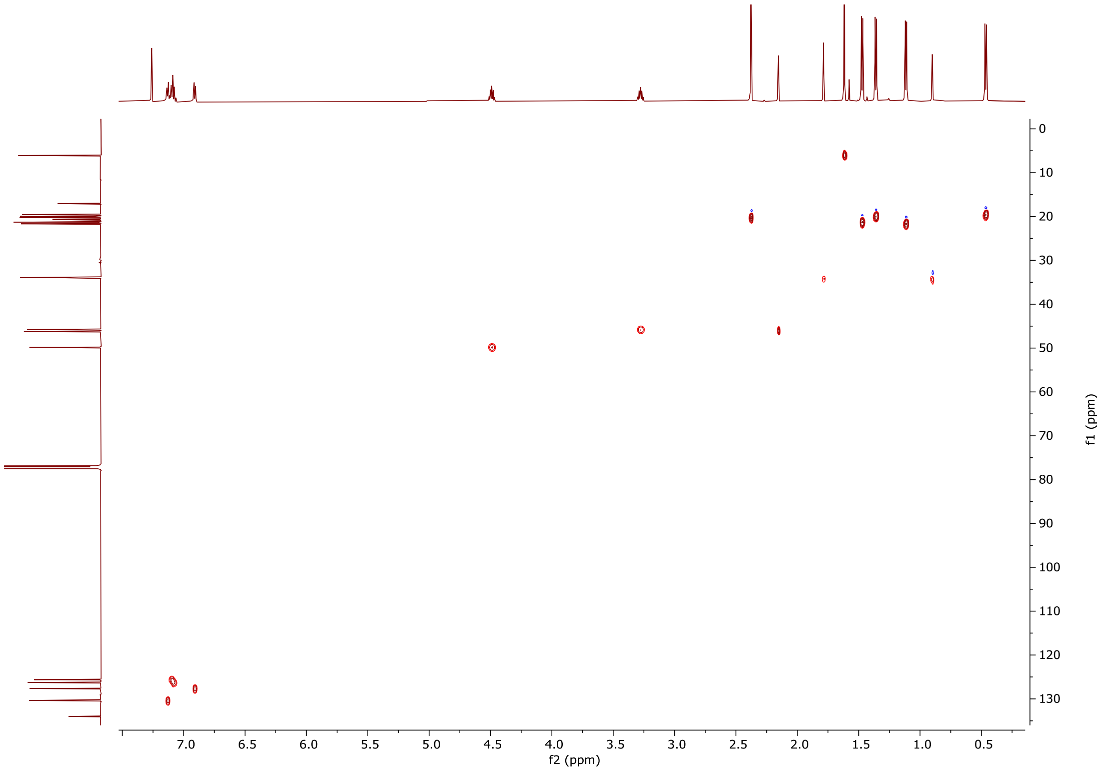

$^{1}H/^{13}C$ HMBC (600/151 MHz, Chloroform-*d*)

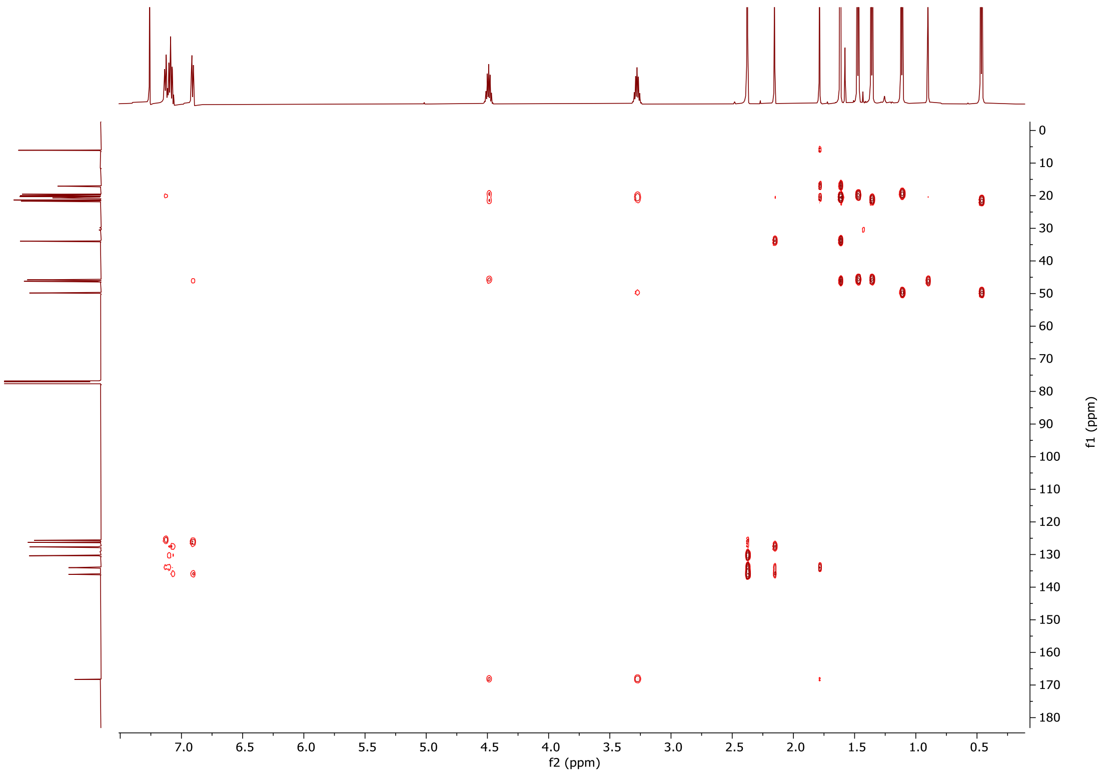

^{1}H NOSEY (600 MHz, Chloroform-*d*)

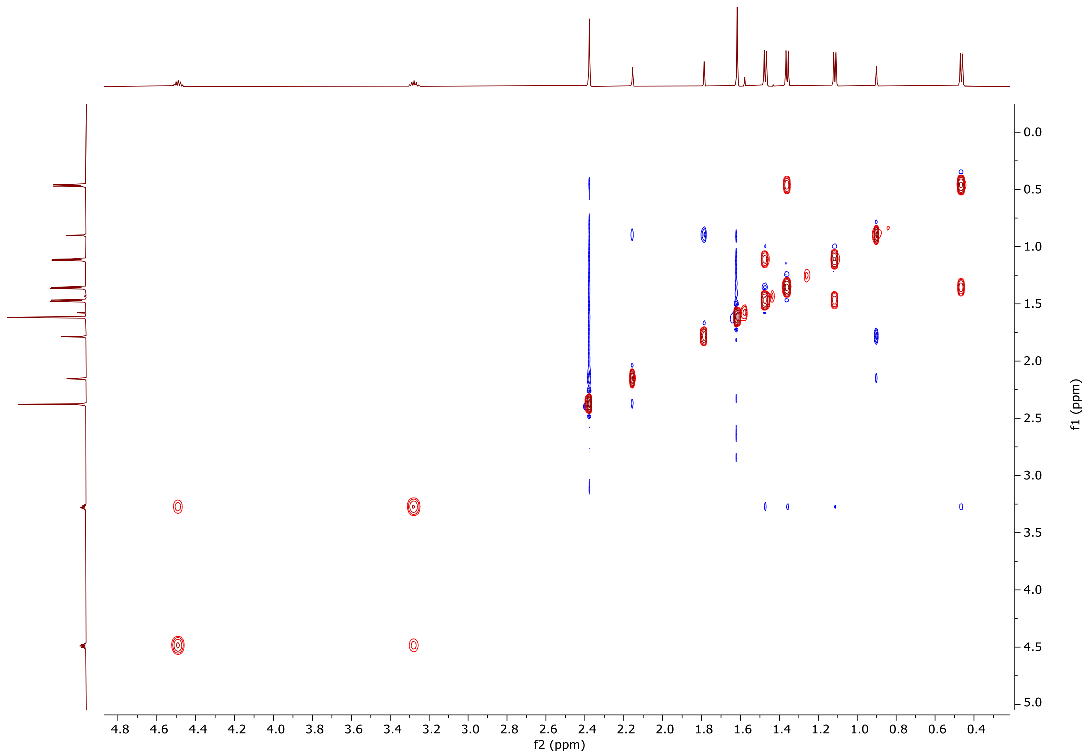

((3a*R*,5*R*,5a*S*,8a*S*,8b*R*)-2,2,7,7-tetramethyltetrahydro-5*H*-bis([1,3]dioxolo)[4,5-b:4',5'-d]pyran-5-yl)methyl 4-((1*S/R,2*S/R**,3*S/R**)-1-(diisopropylcarbamoyl)-3-methylbicyclo[1.1.0]butan-2-yl)benzoate, 3h**

^{1}H NMR (600 MHz, Chloroform-*d*)

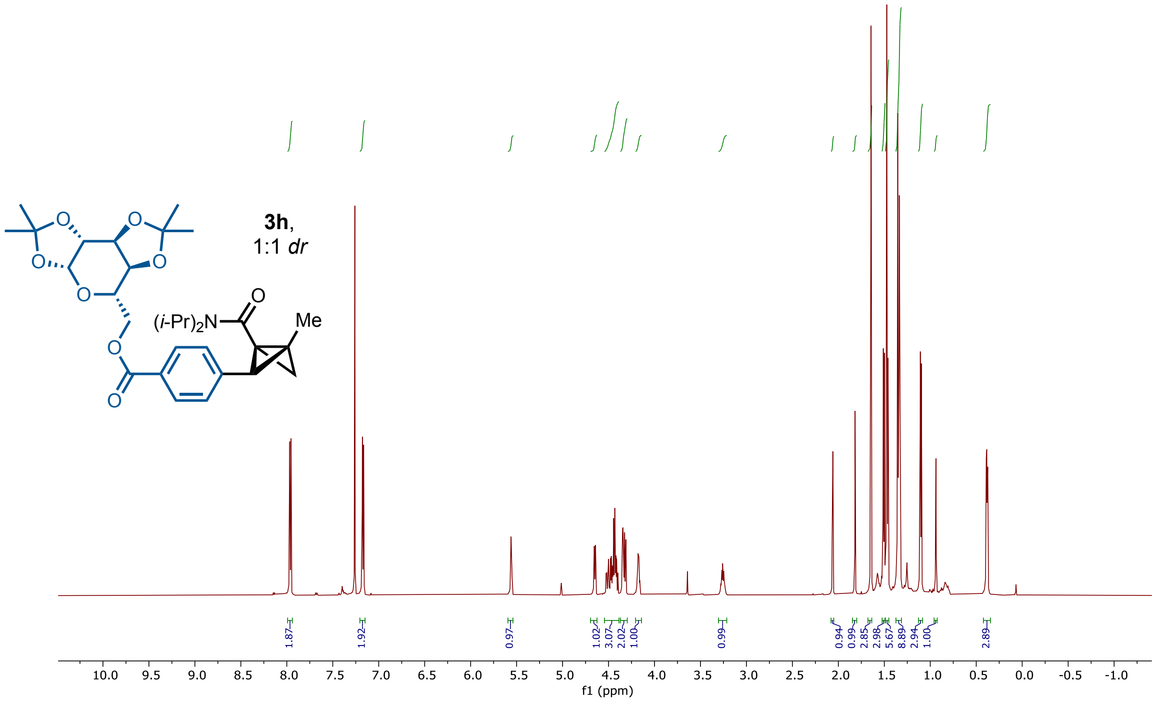

^{13}C NMR (151 MHz, Chloroform-*d*)

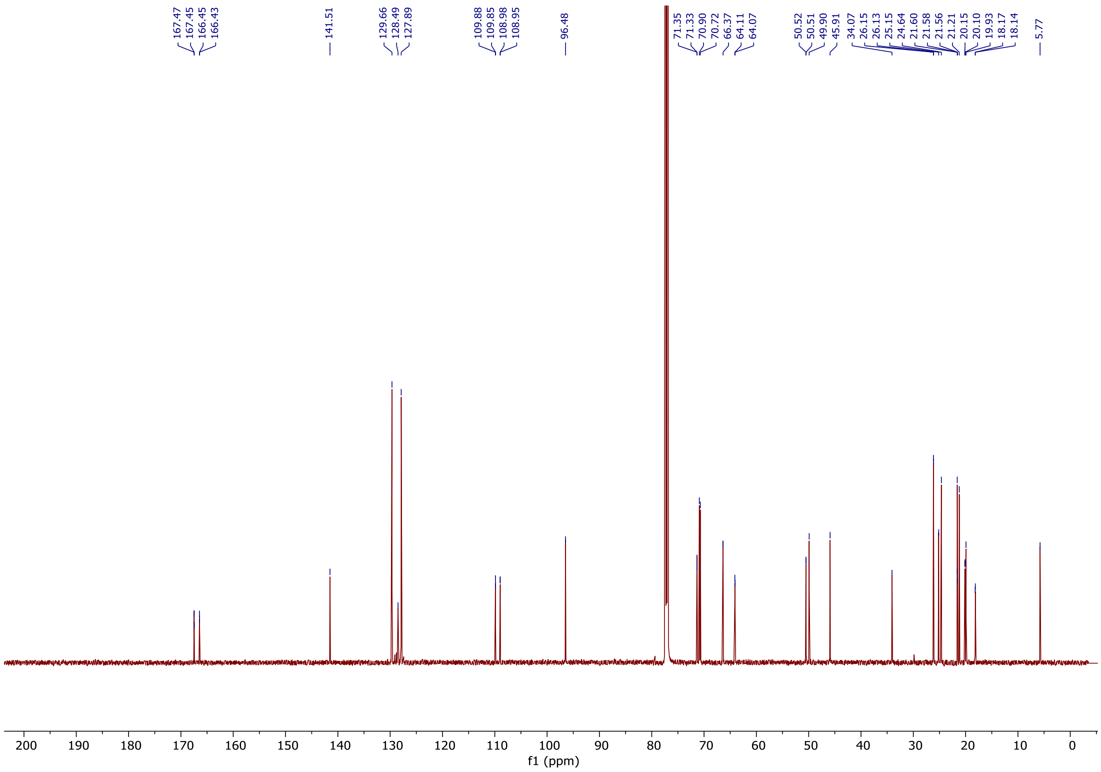

^{1}H COSY (600 MHz, Chloroform-*d*)

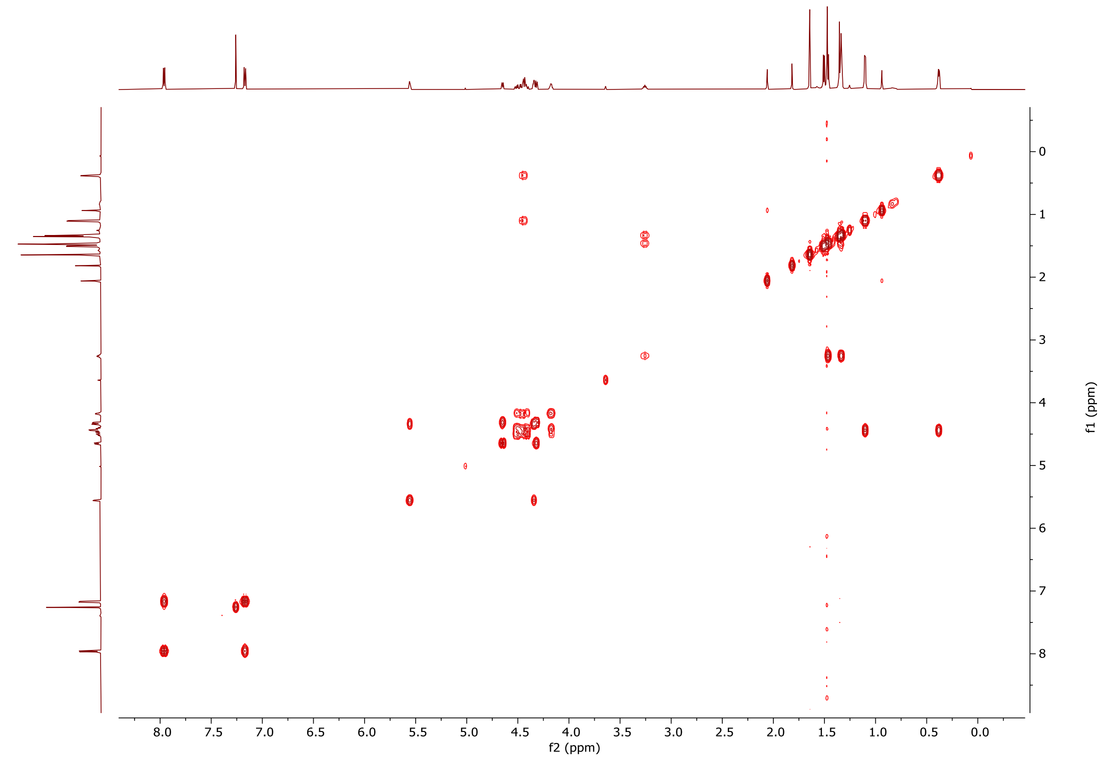

^{1}H/^{13}C HSQC (600/151 MHz, Chloroform-*d*)

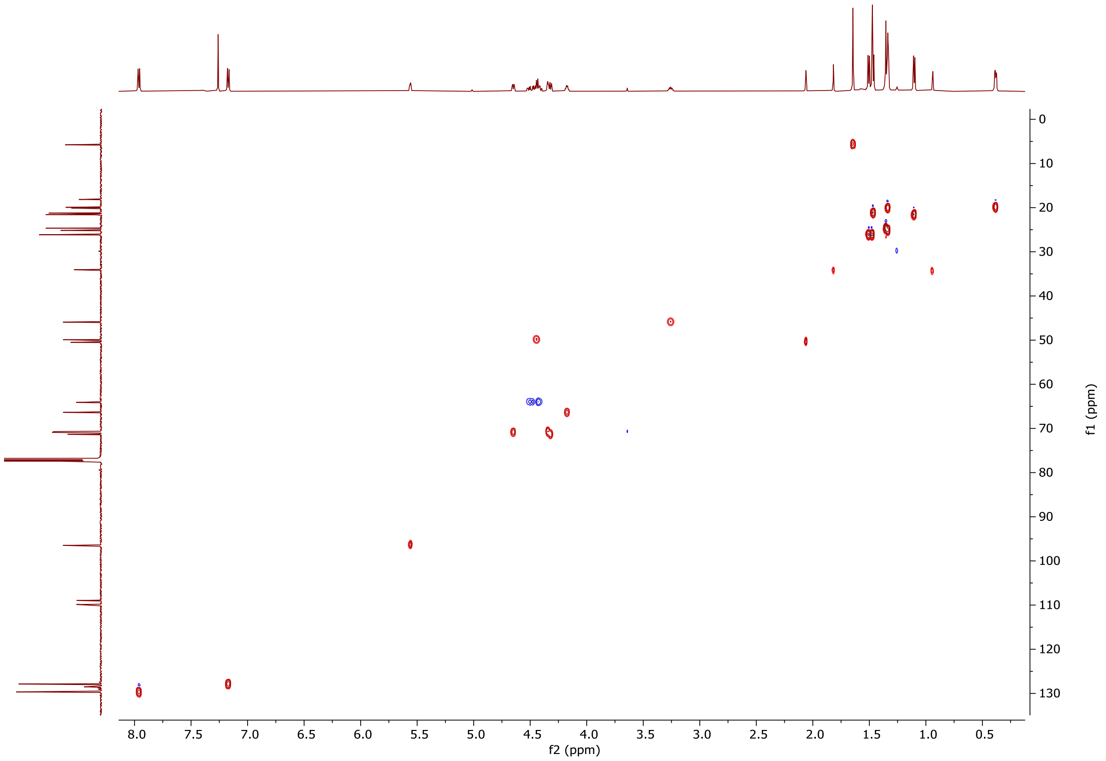

$^{1}H/^{13}C$ **HMBC** (600/151 MHz, Chloroform-*d*)

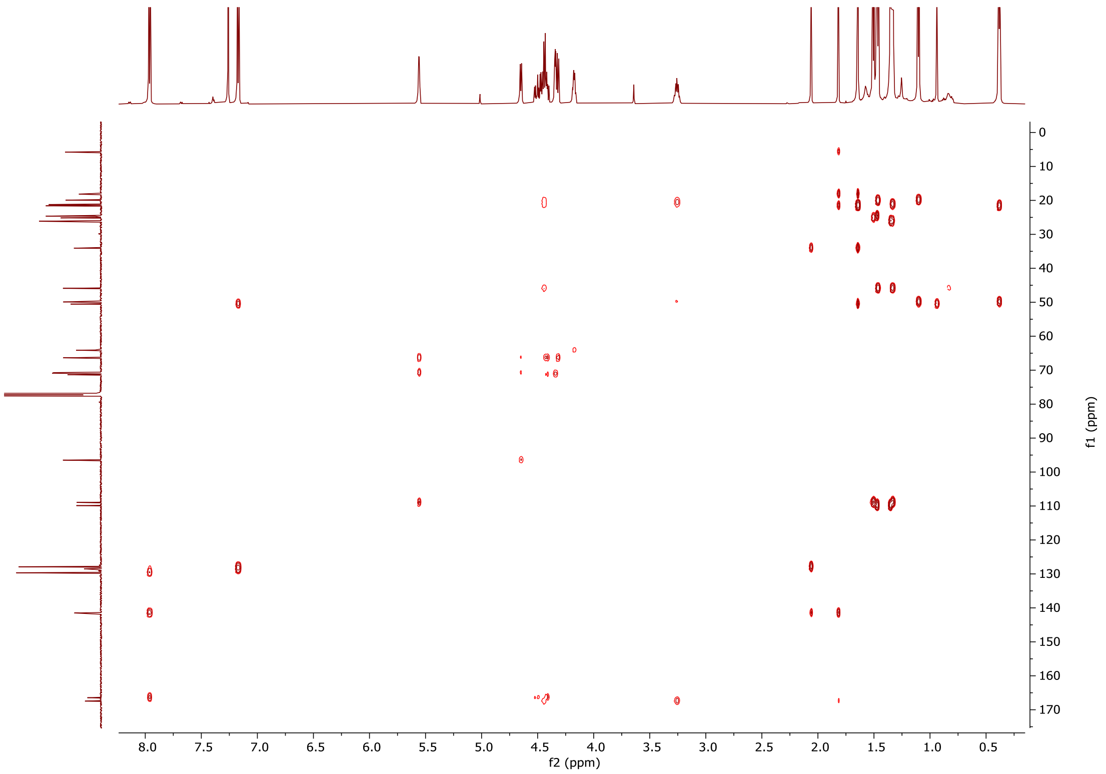

^{1}H **NOSEY** (600 MHz, Chloroform-*d*)

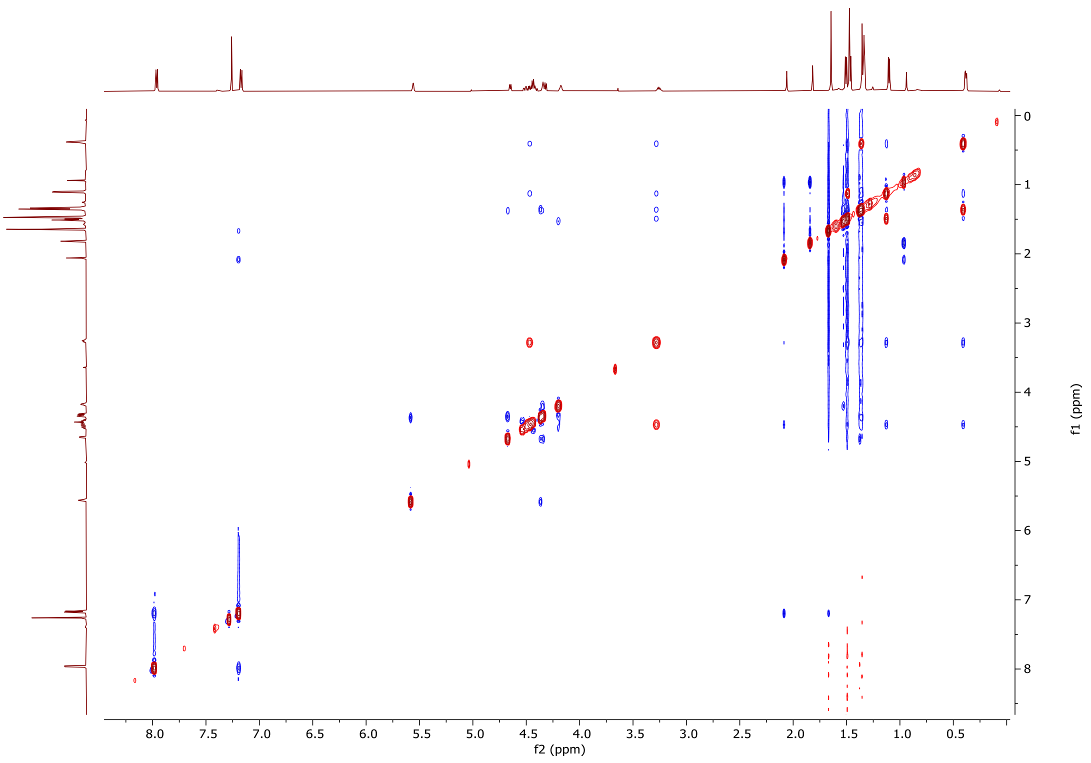

Ethyl (*Z*)-3-((1*S,3*S**)-1-(diisopropylcarbamoyl)-3-methylbicyclo[1.1.0]butan-2-yl)acrylate, 3i**

^{1}H NMR (600 MHz, Chloroform-*d*)

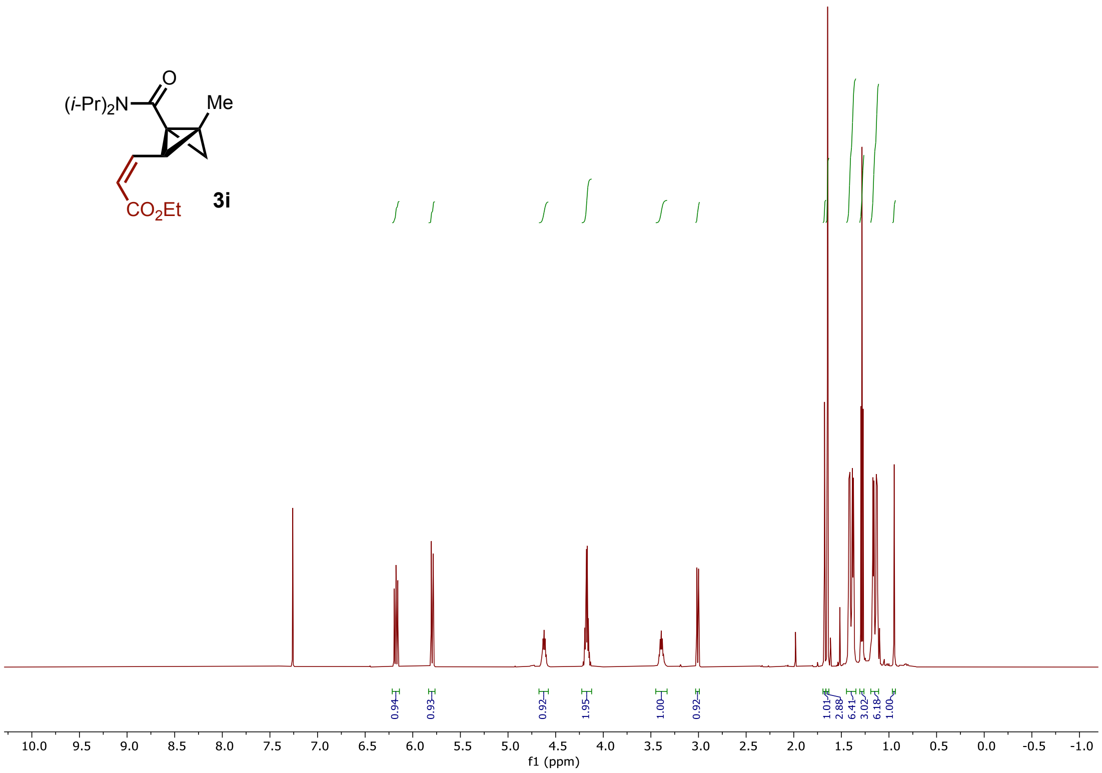

^{13}C NMR (151 MHz, Chloroform-*d*)

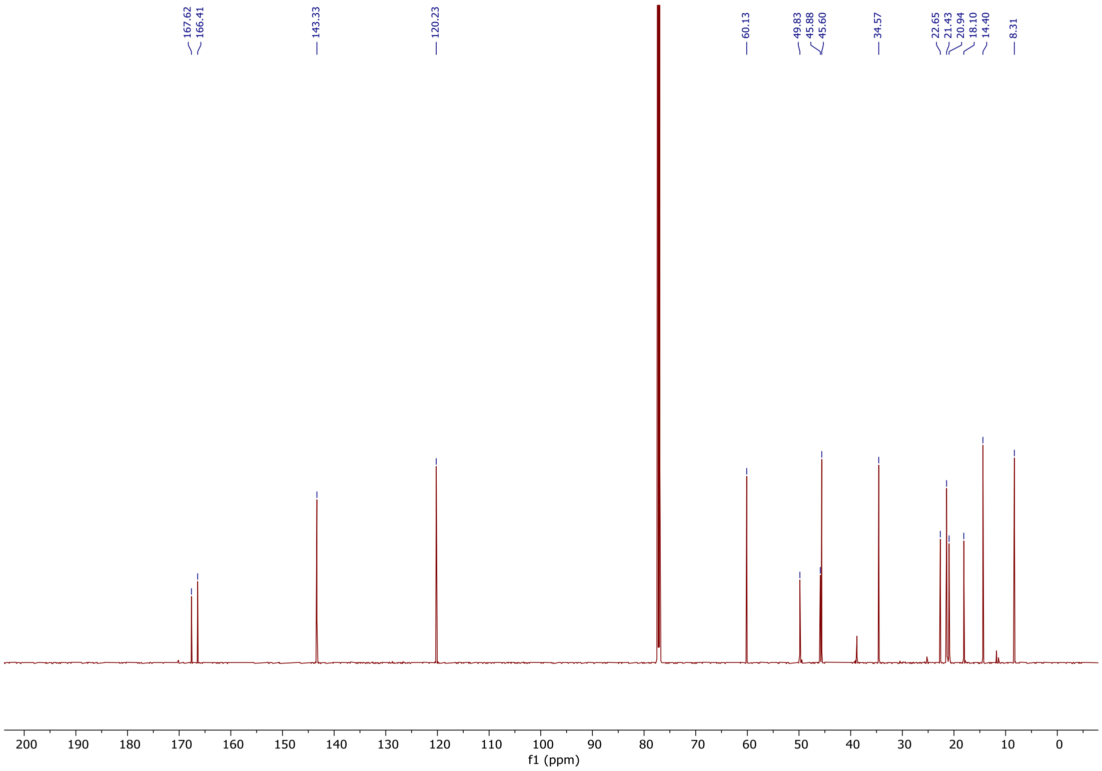

¹H COSY (600 MHz, Chloroform-*d*)

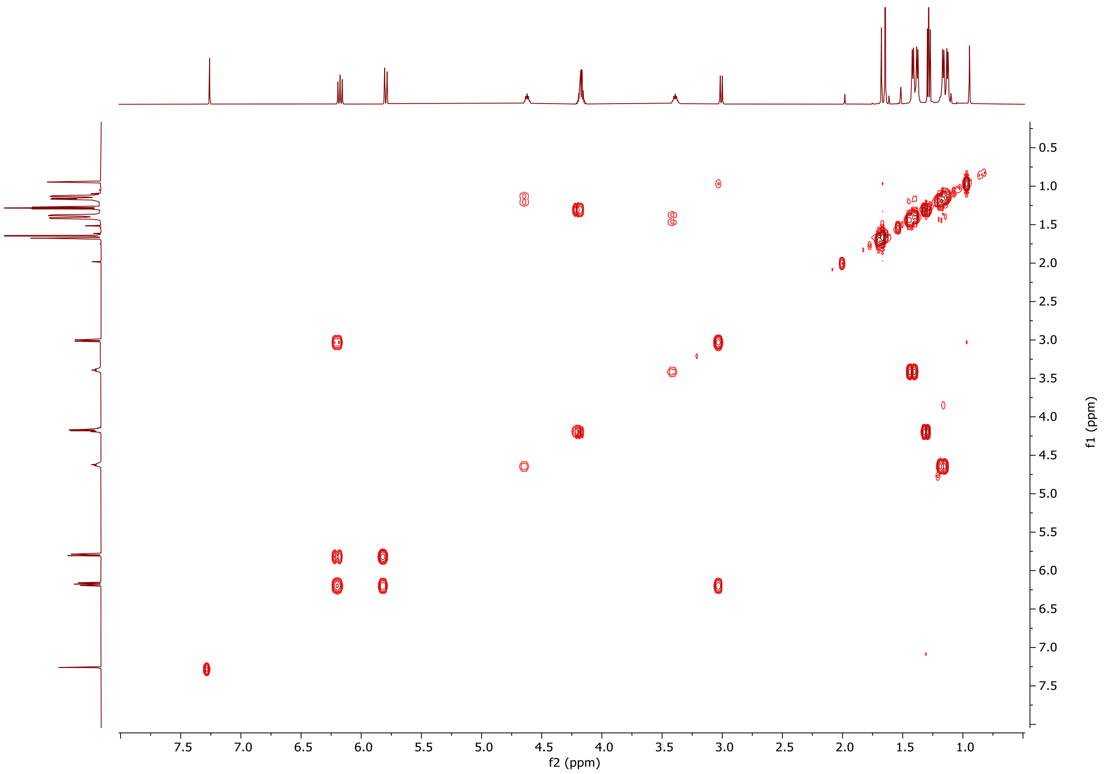

¹H/¹³C HSQC (600/151 MHz, Chloroform-*d*)

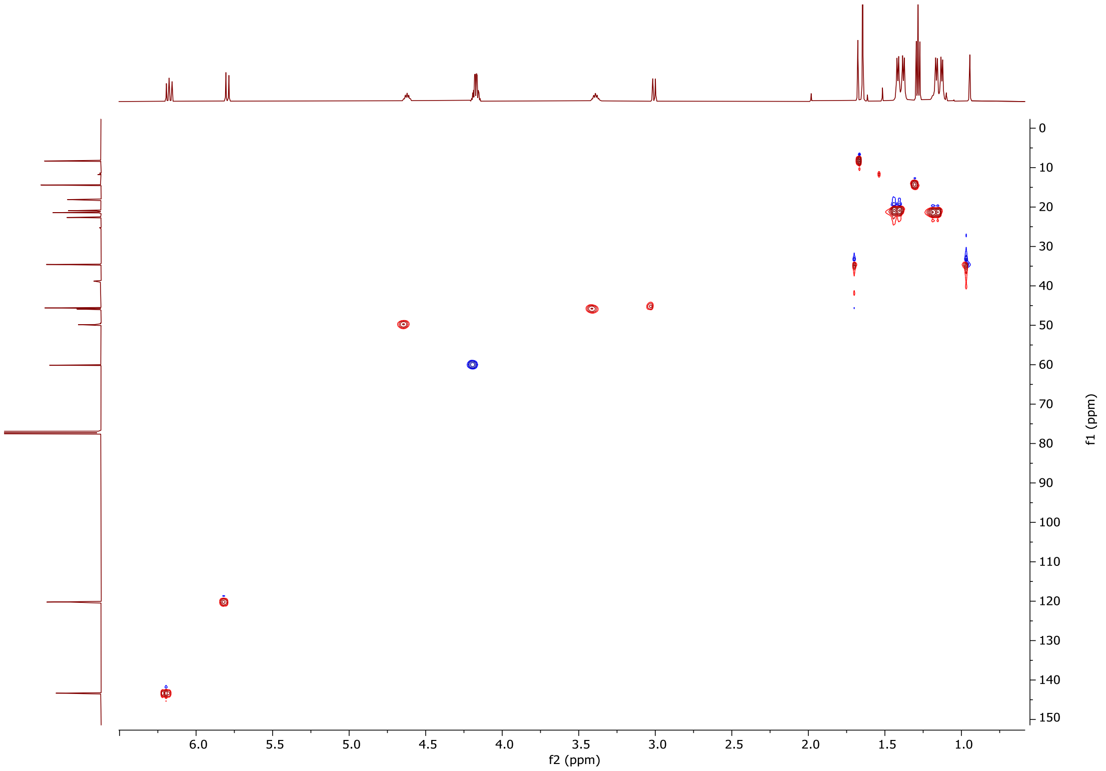

$^{1}H/^{13}C$ HMBC (600/151 MHz, Chloroform-*d*)

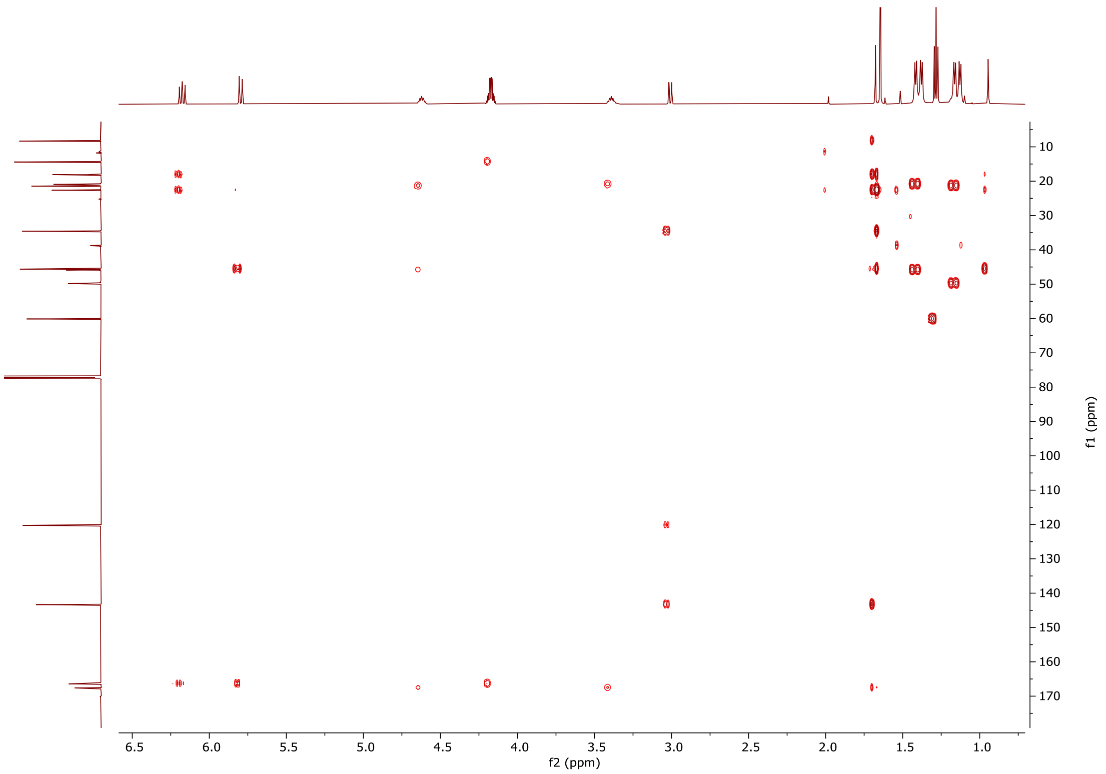

^{1}H NOSEY (600 MHz, Chloroform-*d*)

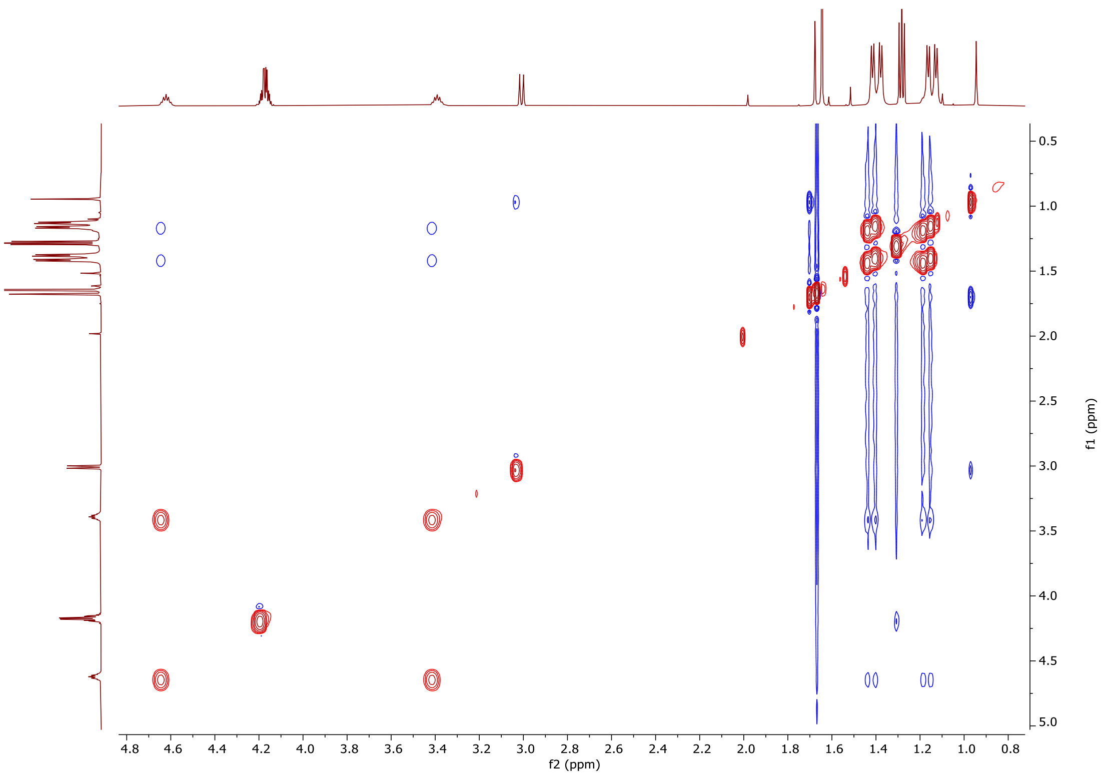

(1*S,3*S**)-*N*,*N*-diisopropyl-3-methyl-2-(pyridin-2-yl)bicyclo[1.1.0]butane-1-carboxamide, 3j**

^{1}H NMR (600 MHz, Chloroform-*d*)

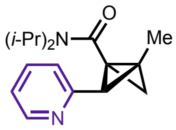

3j

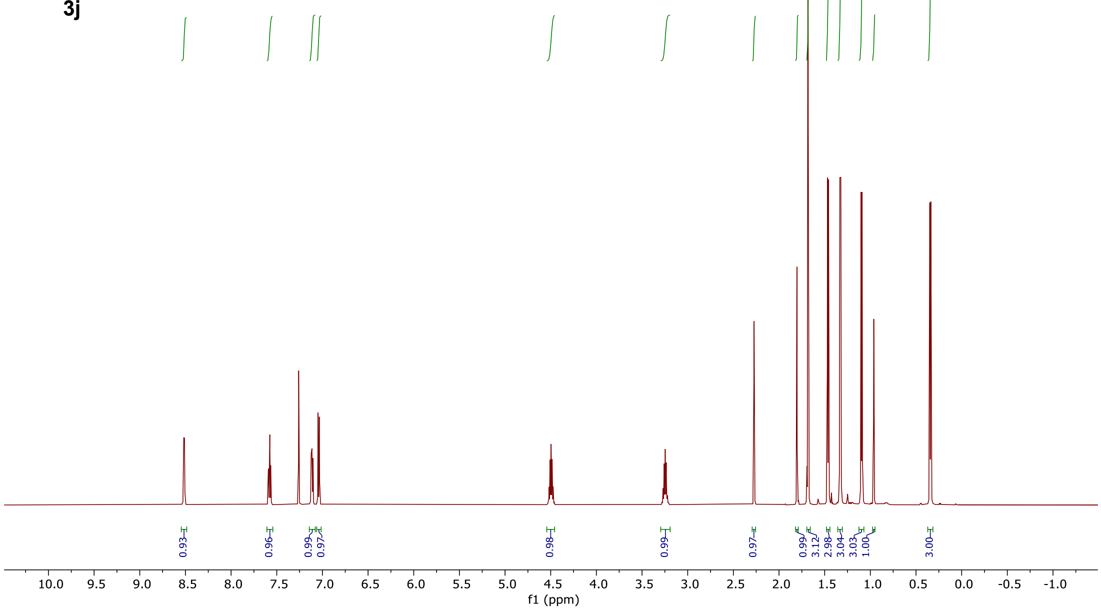

^{13}C NMR (151 MHz, Chloroform-*d*)

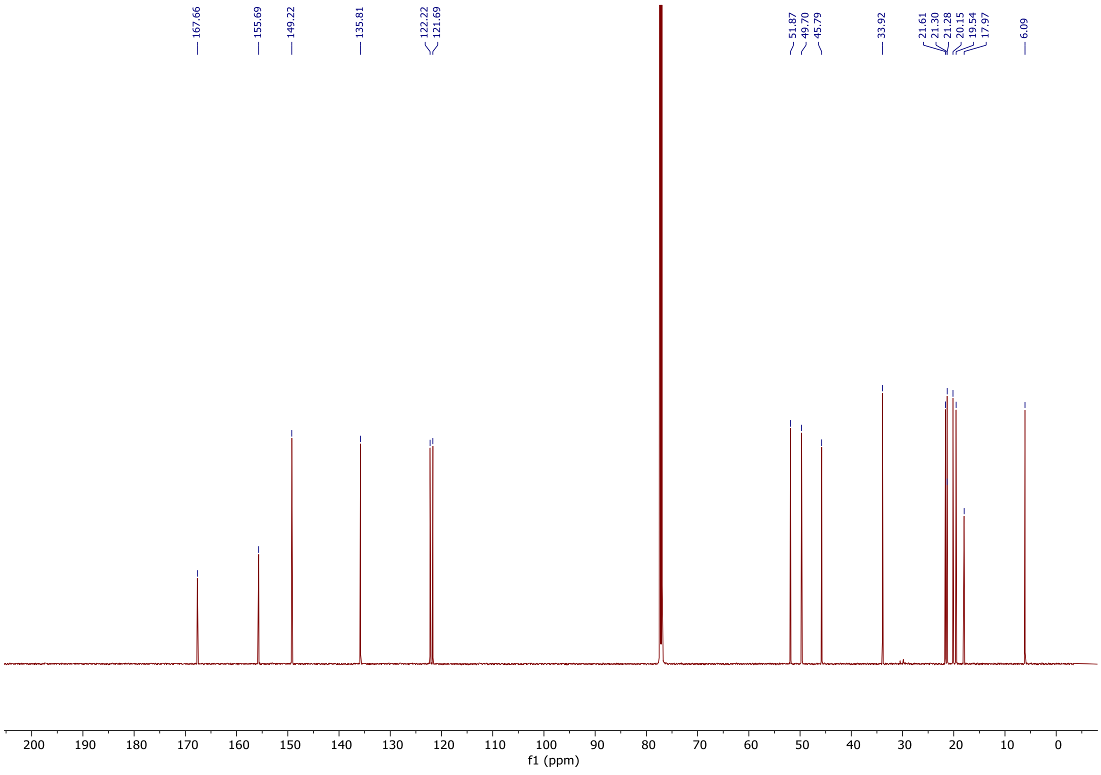

^{1}**H COSY** (600 MHz, Chloroform-*d*)

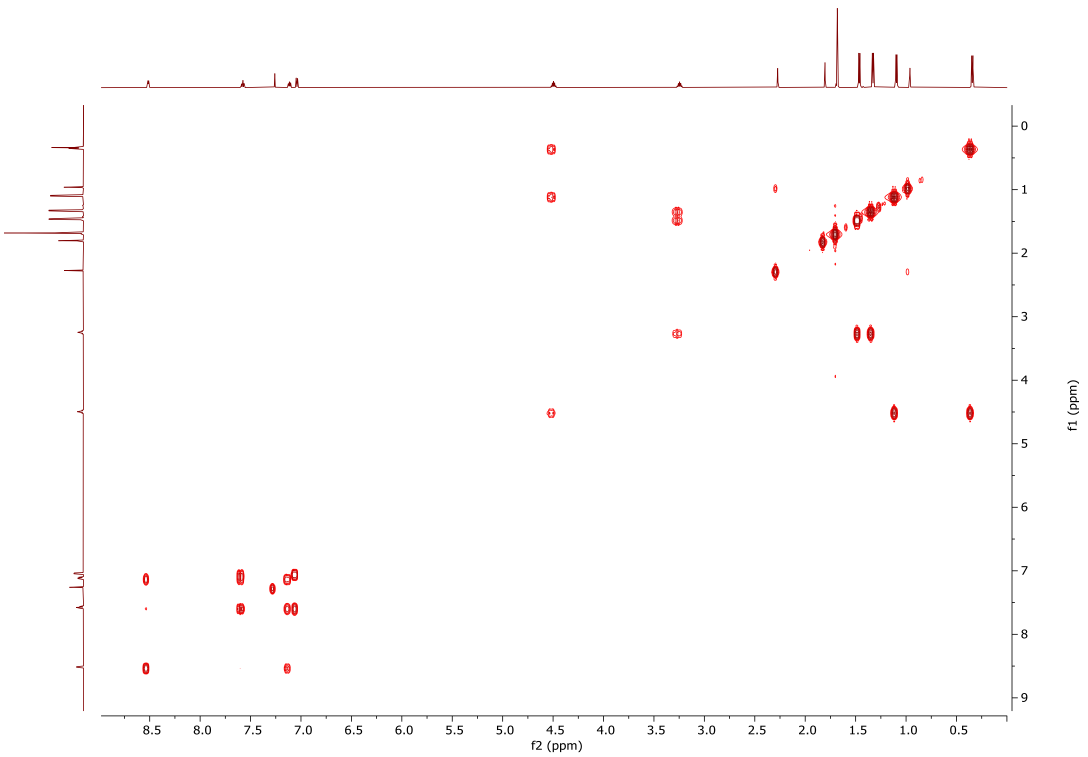

^{1}**H/**^{13}**C HSQC** (600/151 MHz, Chloroform-*d*)

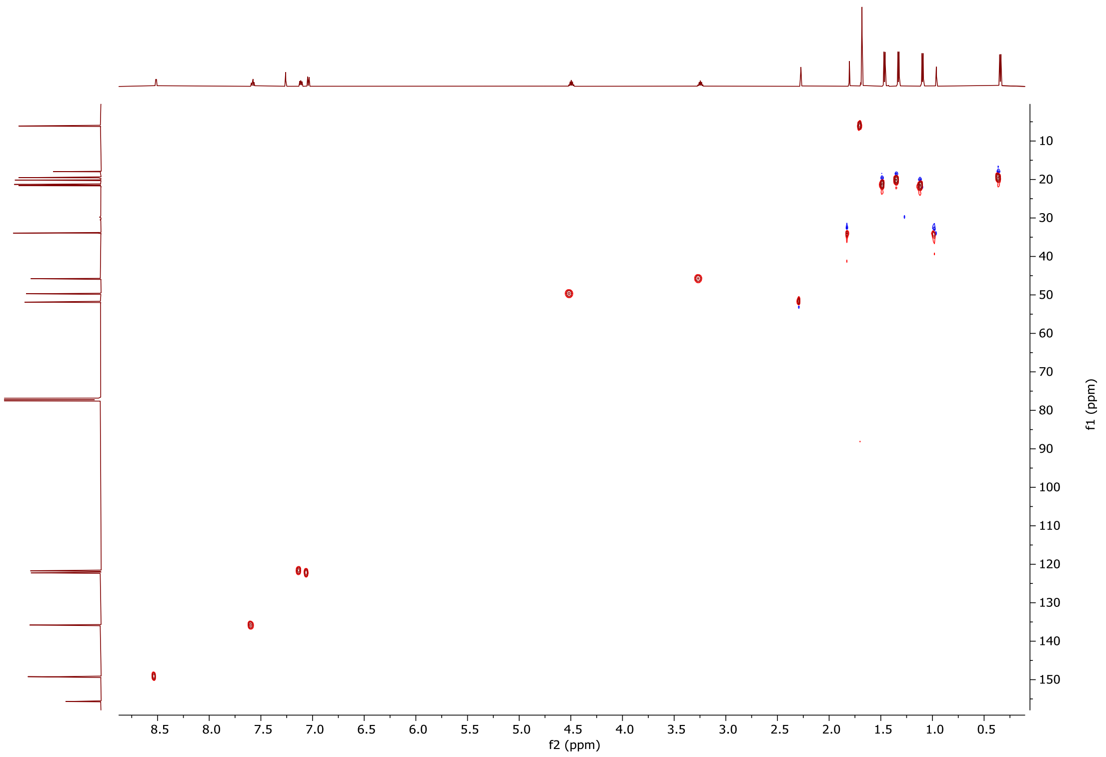

$^1H/^{13}C$ HMBC (600/151 MHz, Chloroform-*d*)

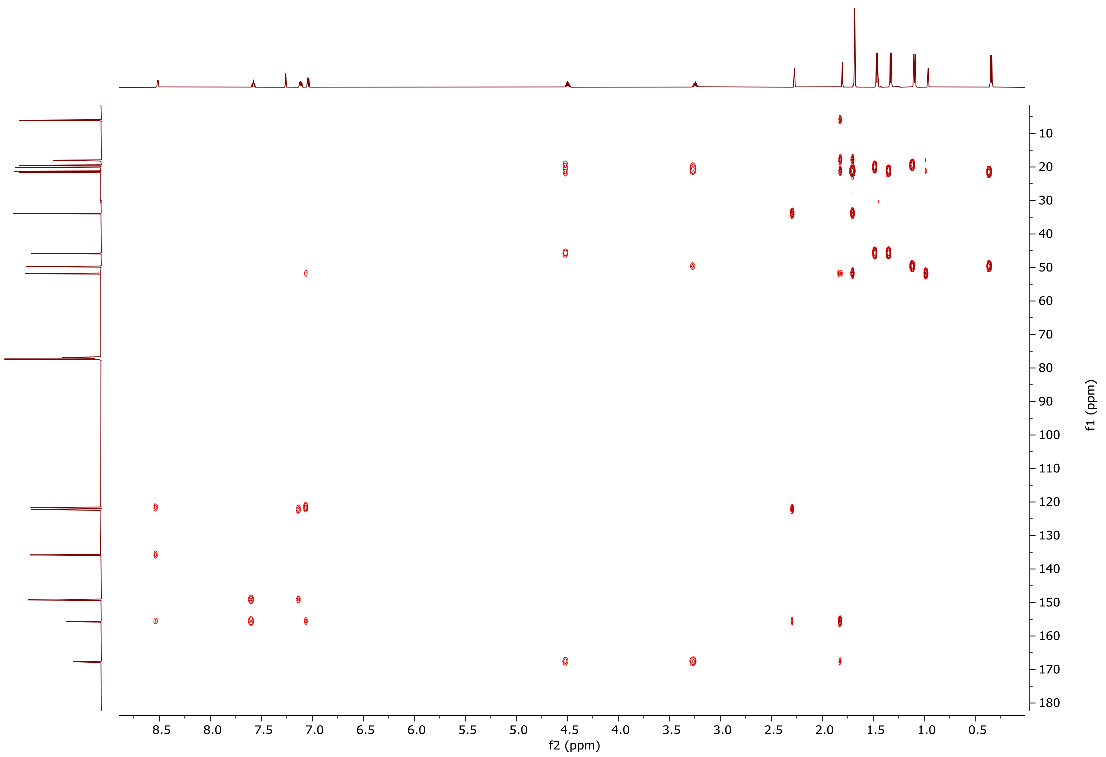

^1H NOSEY (600 MHz, Chloroform-*d*)

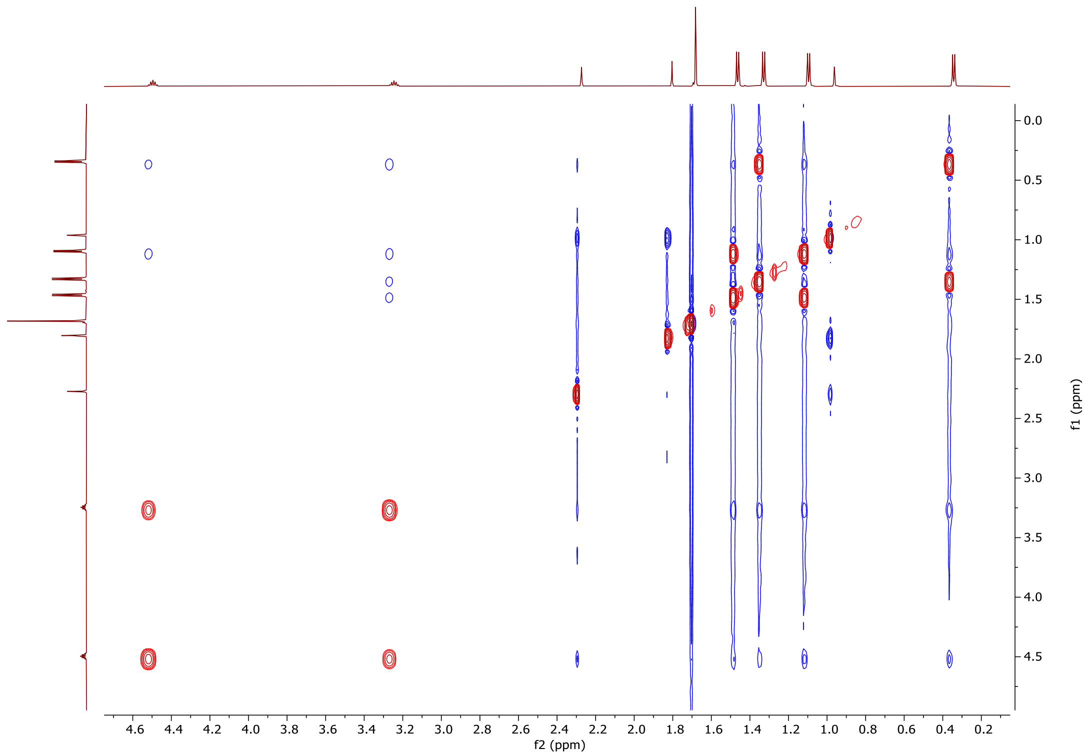

***Tert-butyl* 5-((1S*,3S*)-1-(diisopropylcarbamoyl)-3-methylbicyclo[1.1.0]butan-2-yl)-1*H*-indole-1-carboxylate, 3k**

^{1}H NMR (400 MHz, Chloroform-*d*)

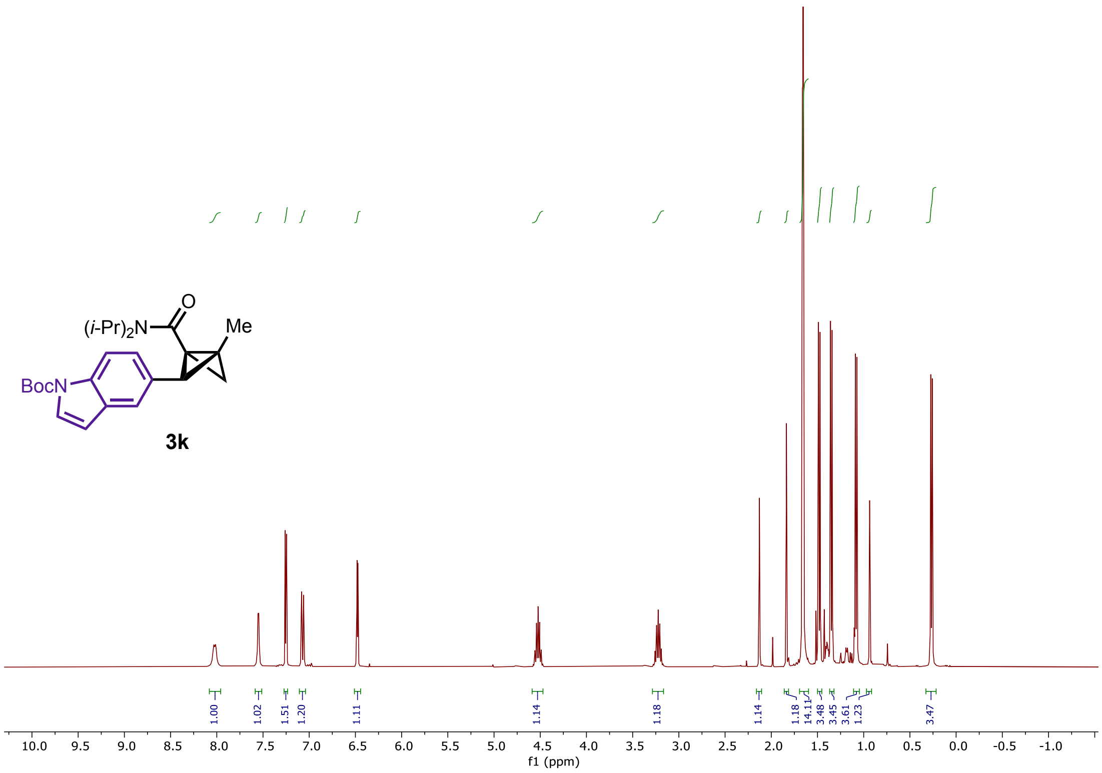

^{13}C NMR (101 MHz, Chloroform-*d*)

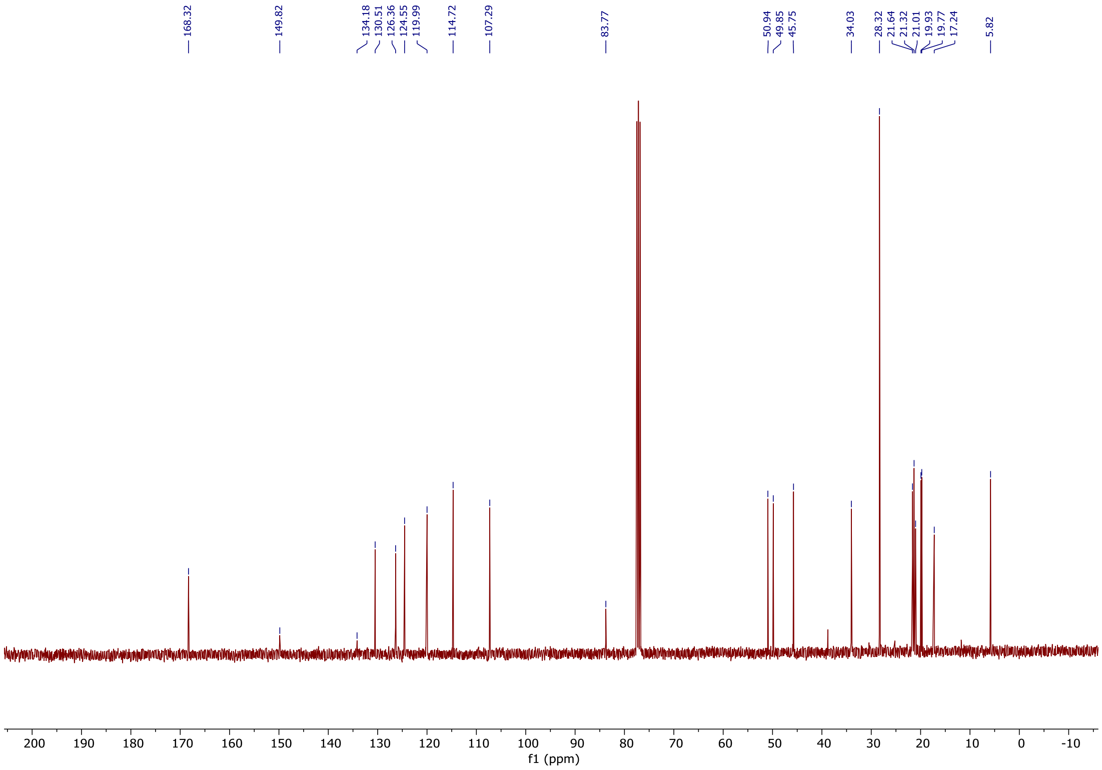

^{1}H COSY (400 MHz, Chloroform-*d*)

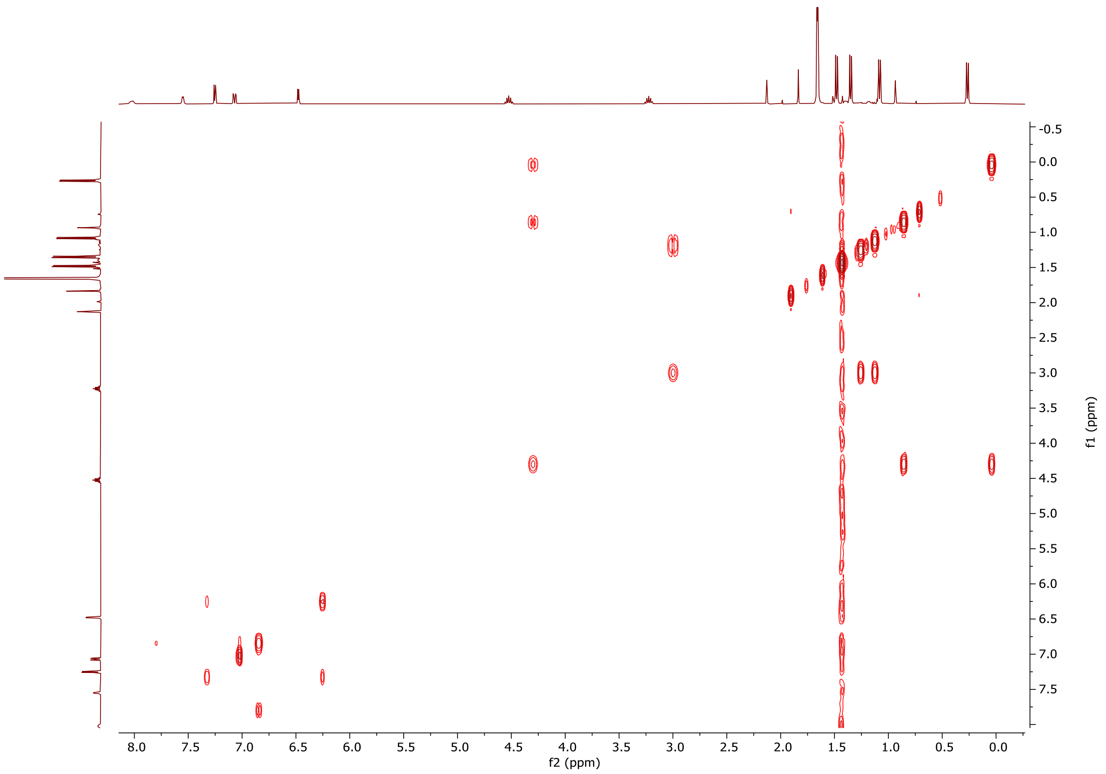

^{1}H/^{13}C HSQC (400/101 MHz, Chloroform-*d*)

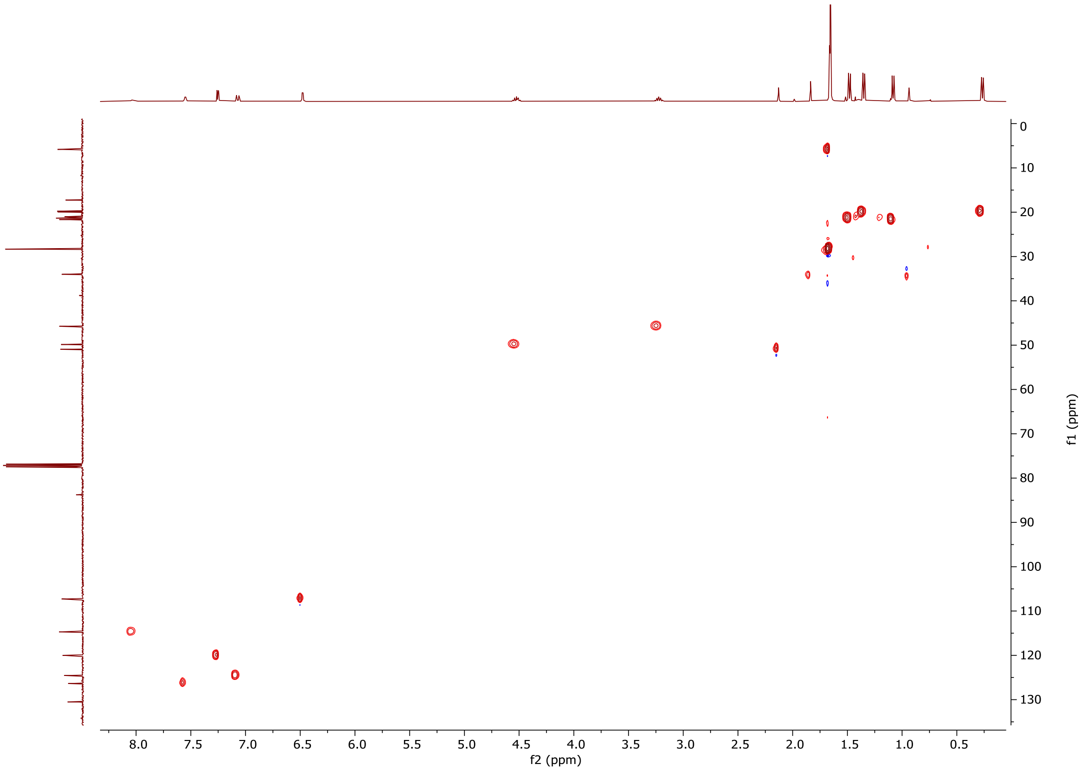

(1*S,3*S**)-2-(7-chloroquinolin-4-yl)-*N*,*N*-diisopropyl-3-methylbicyclo[1.1.0]butane-1-carboxamide, 3l**

^{1}H NMR (600 MHz, Chloroform-*d*)

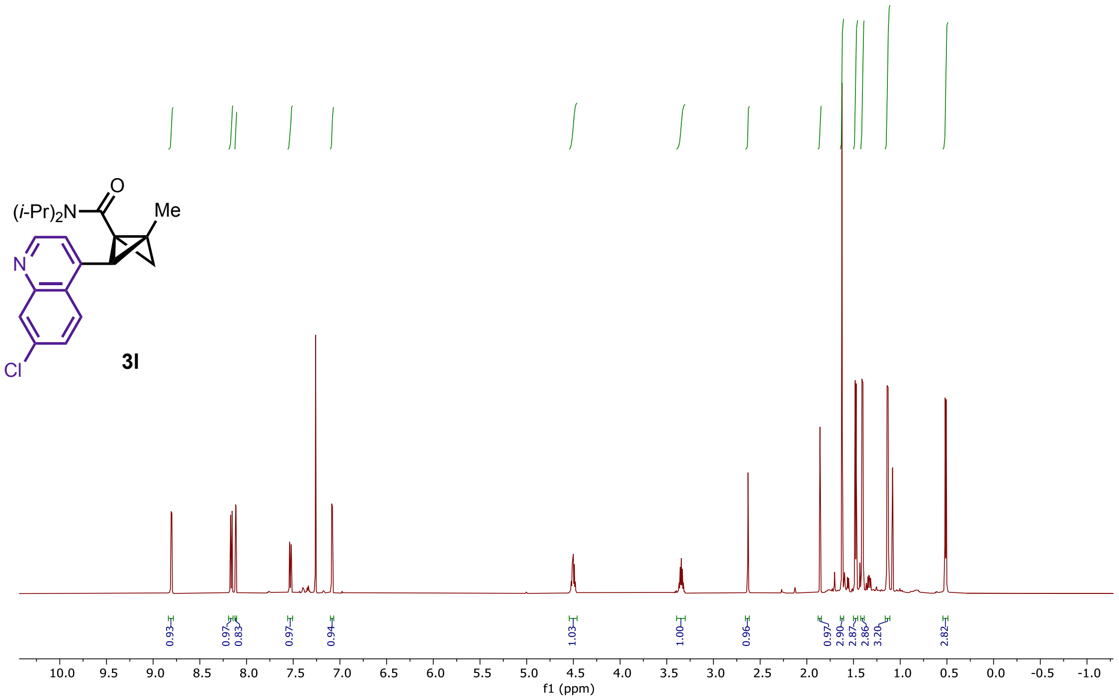

^{13}C NMR (151 MHz, Chloroform-*d*)

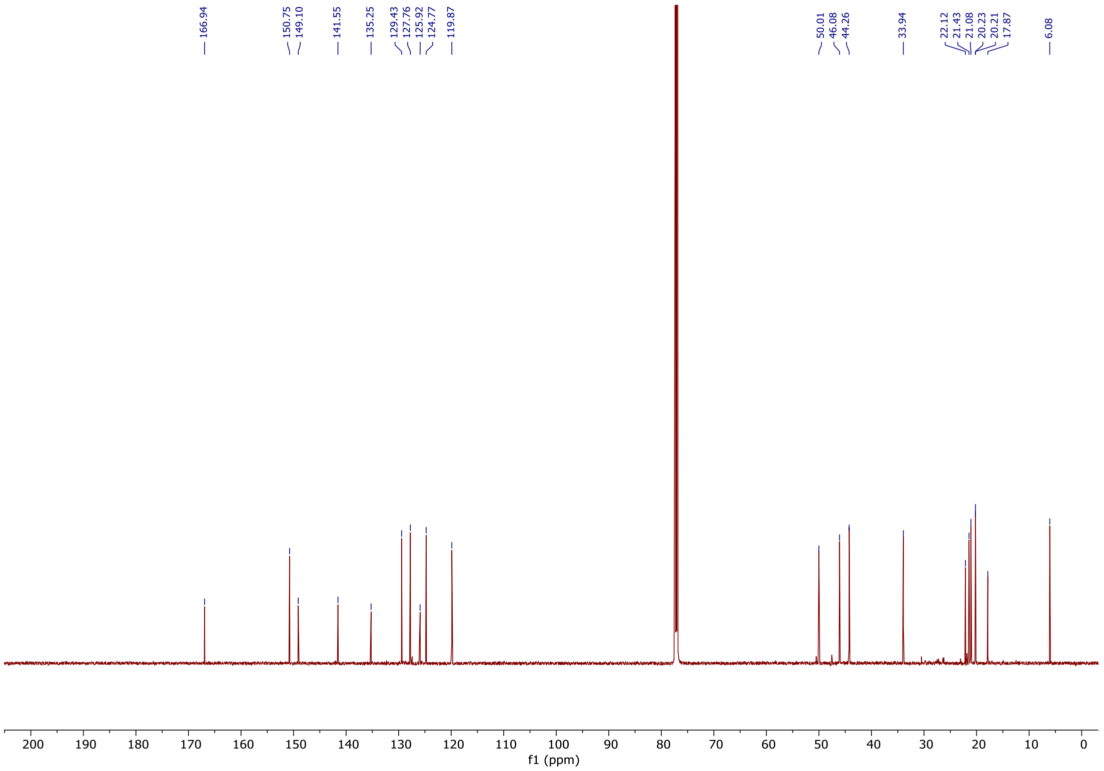

^{1}**H COSY** (600 MHz, Chloroform-*d*)

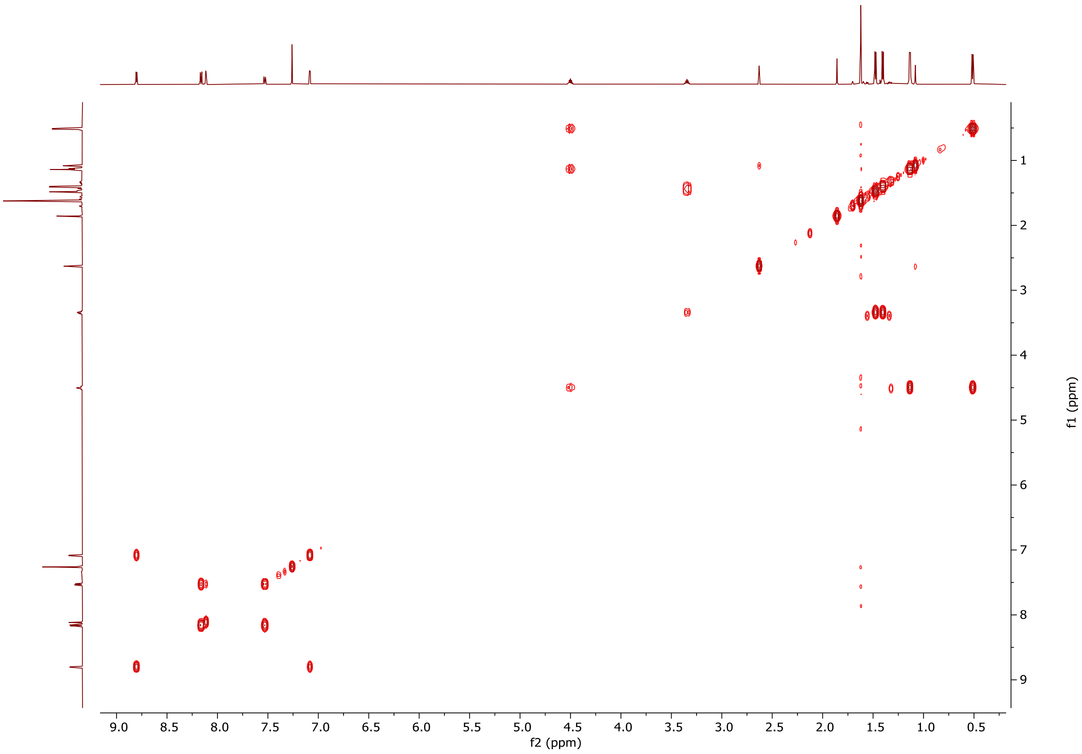

^{1}**H/**^{13}**C HSQC** (600/151 MHz, Chloroform-*d*)

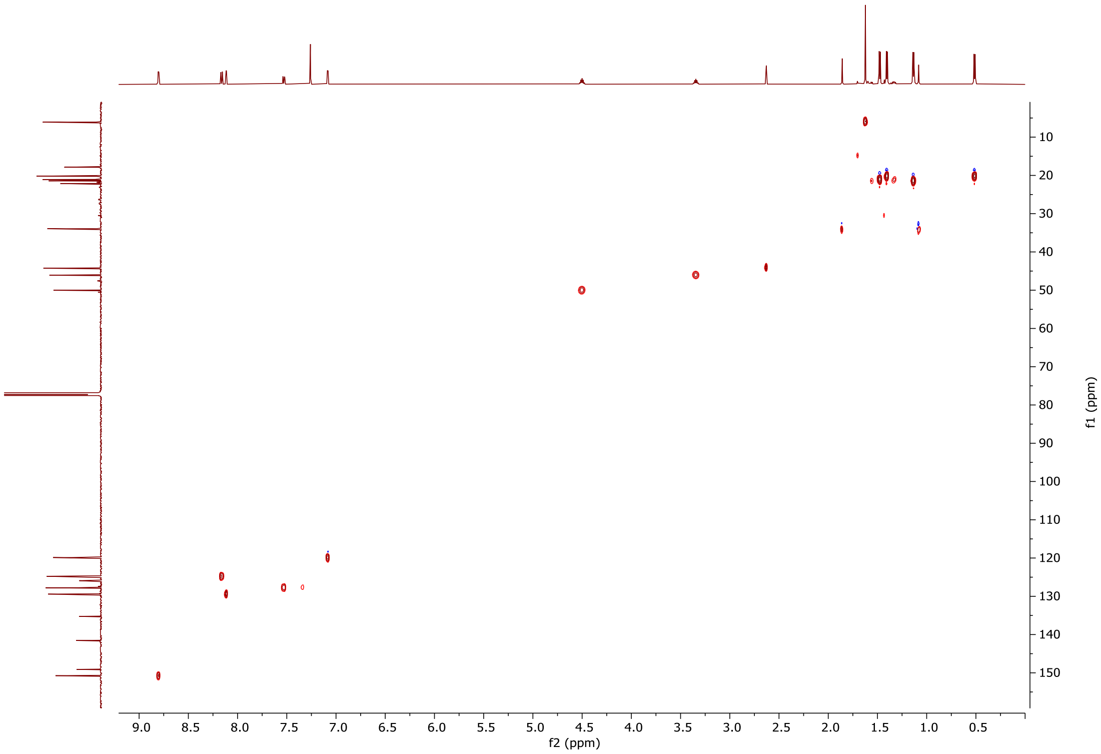

$^{1}H/^{13}C$ HMBC (600/151 MHz, Chloroform-*d*)

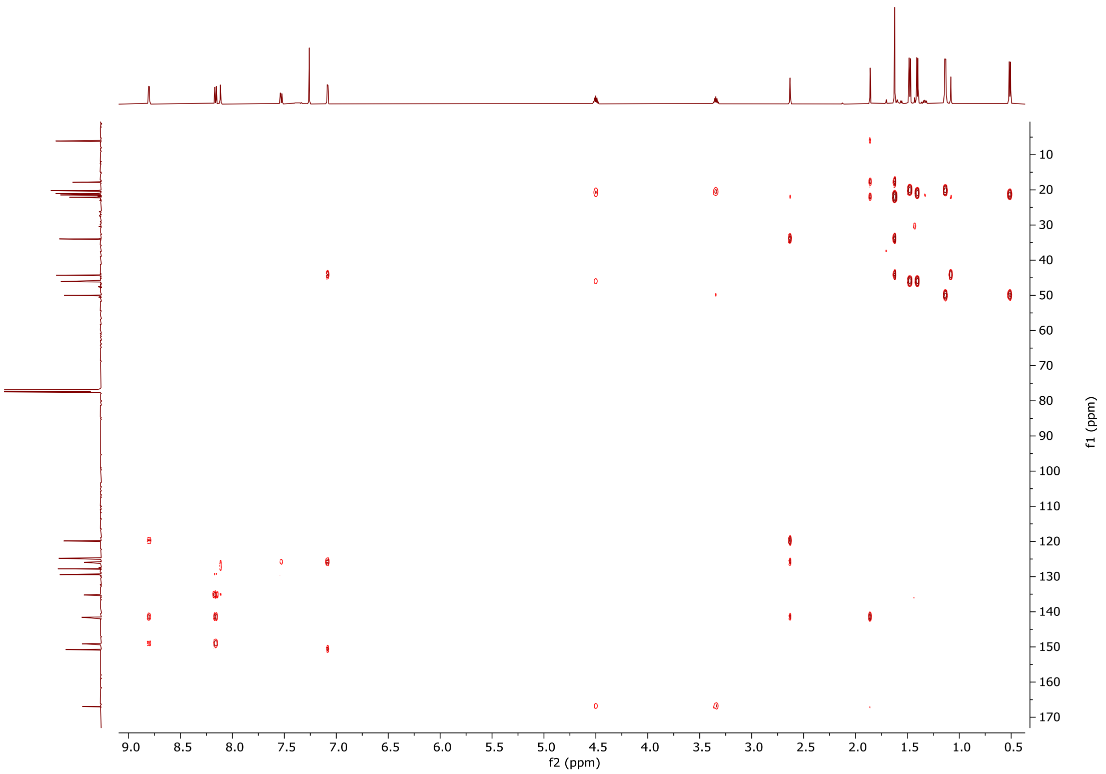

^{1}H NOSEY (600 MHz, Chloroform-*d*)

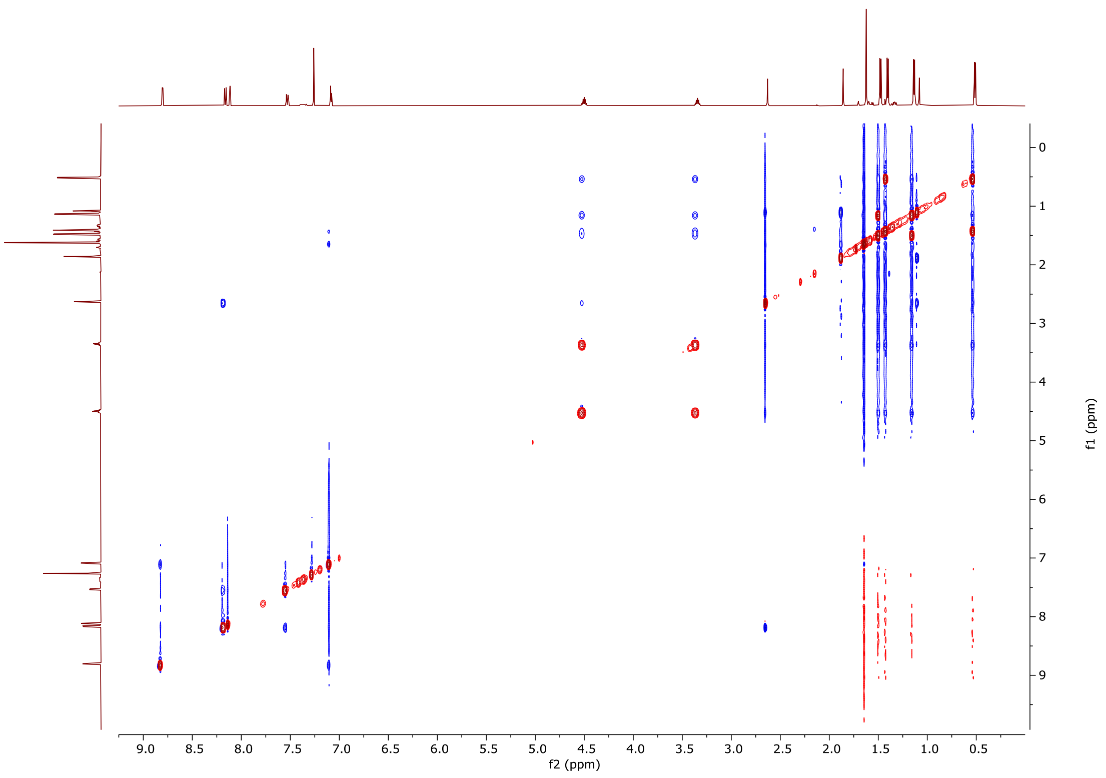

Methyl 4-((1*S,3*S**)-1-(diisopropylcarbamoyl)-3-phenylbicyclo[1.1.0]butan-2-yl)benzoate, 3m**

^{1}H NMR (600 MHz, Chloroform-*d*)

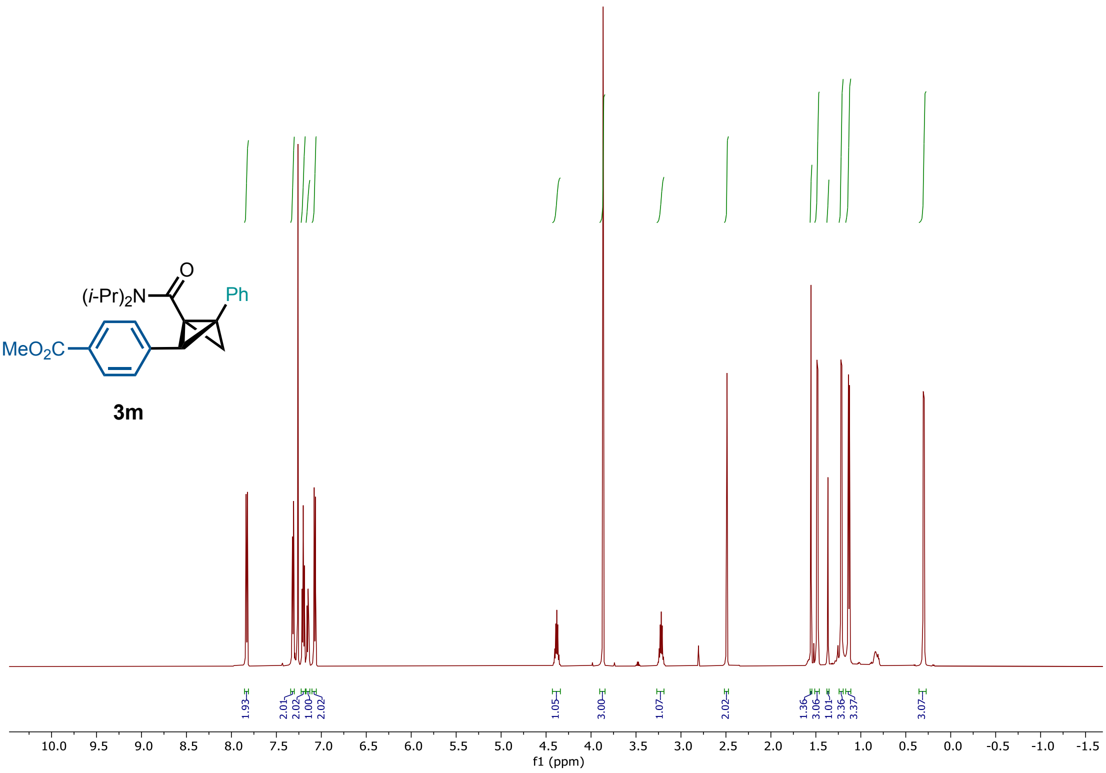

^{13}C NMR (151 MHz, Chloroform-*d*)

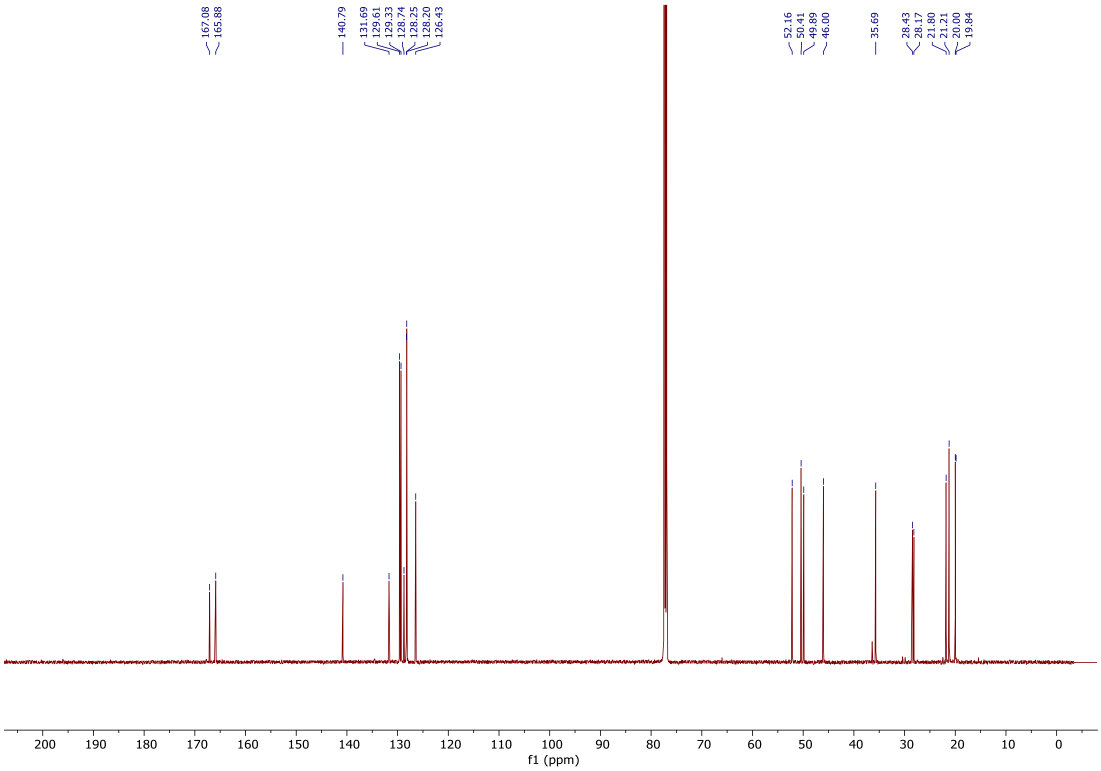

^{1}H COSY (600 MHz, Chloroform-*d*)

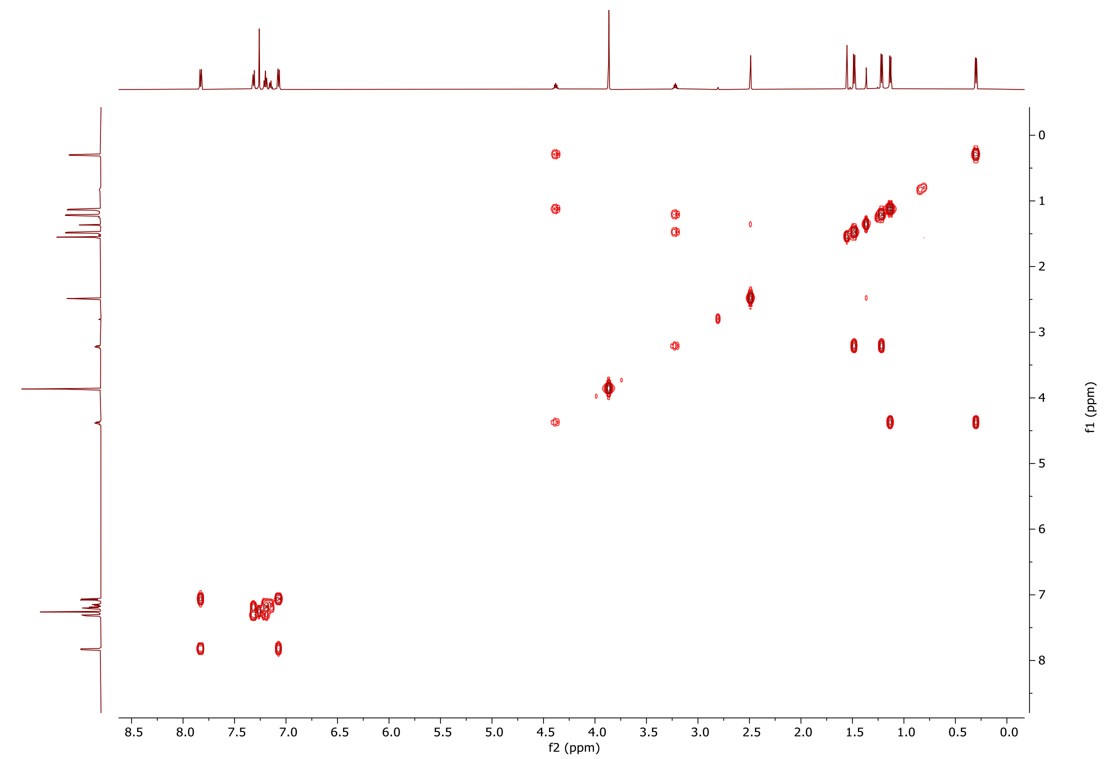

^{1}H/^{13}C HSQC (600/151 MHz, Chloroform-*d*)

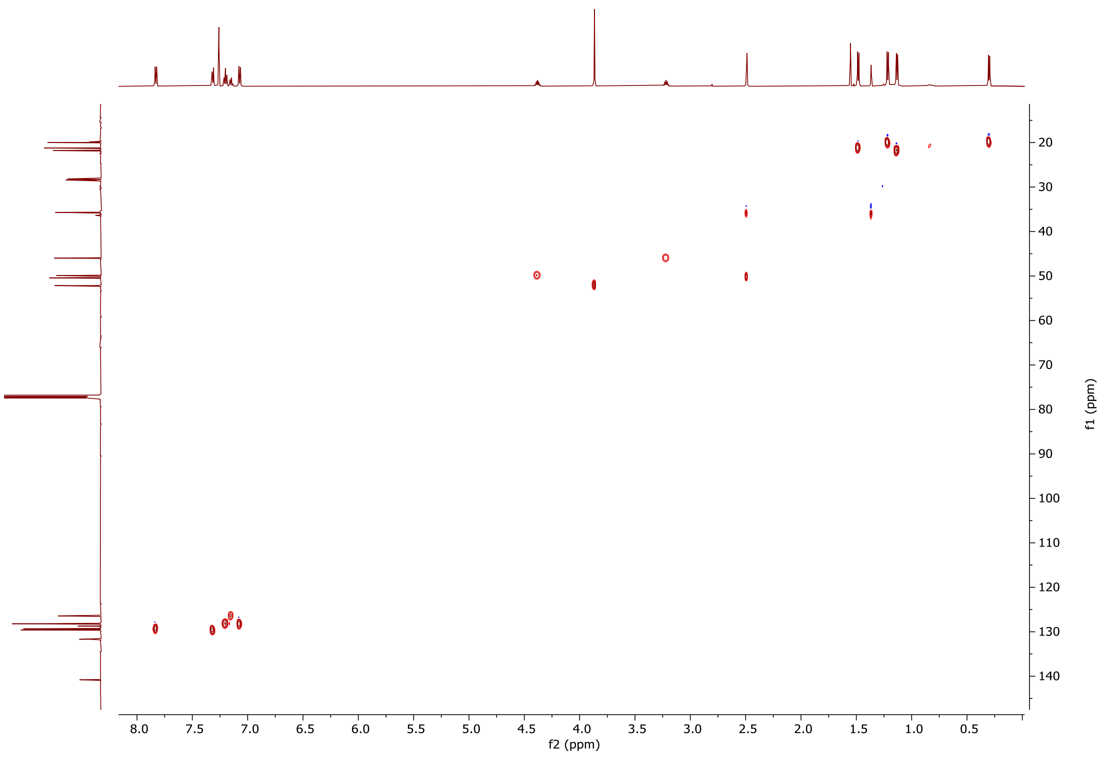

$^{1}H/^{13}C$ **HMBC** (600/151 MHz, Chloroform-*d*)

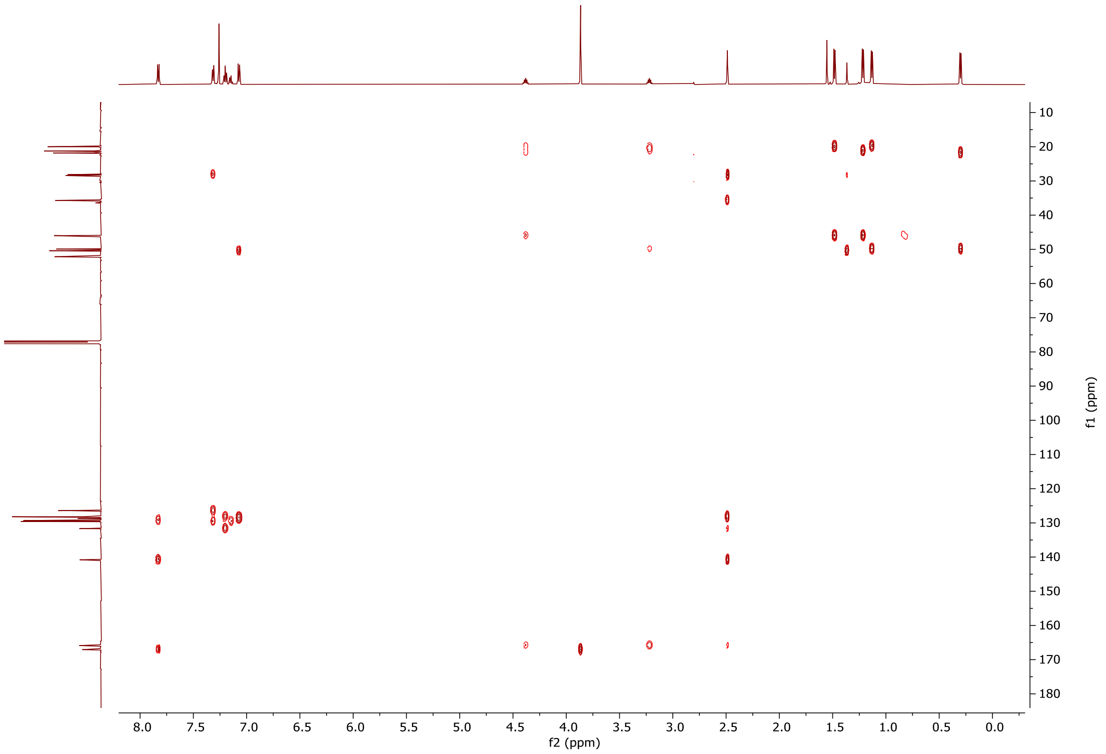

^{1}H **NOSEY** (600 MHz, Chloroform-*d*)

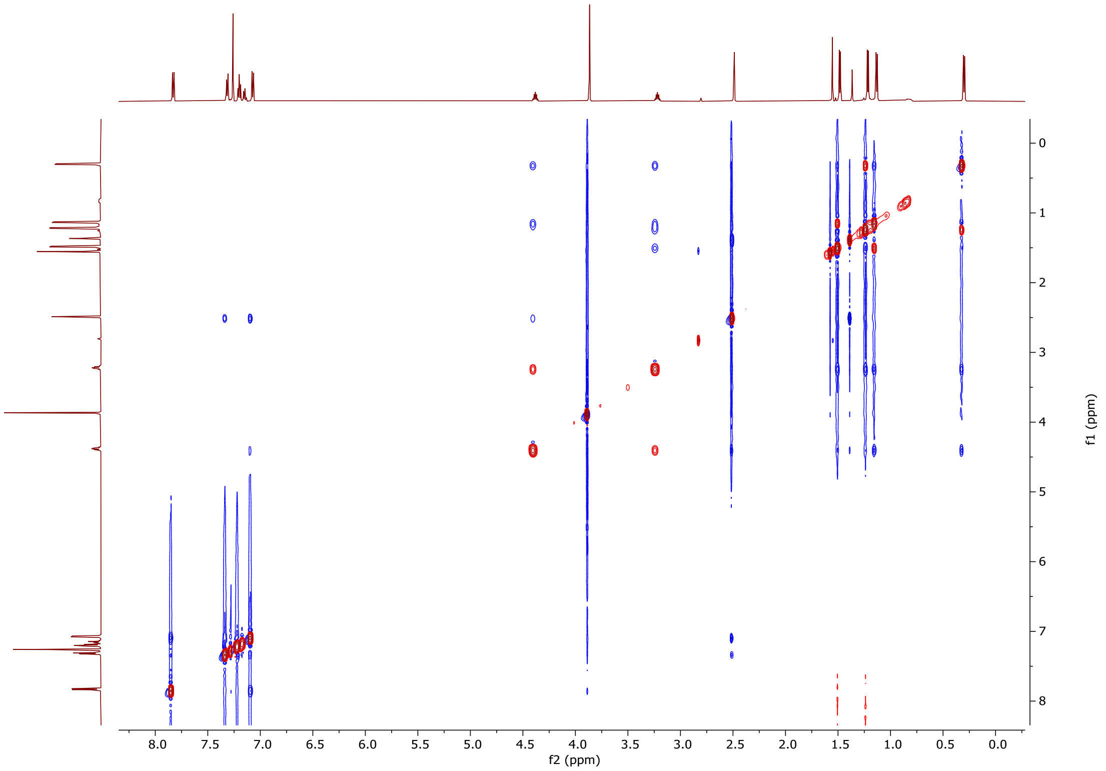

(1*S,3*S**)-*N*,*N*-diisopropyl-2,3-diphenylbicyclo[1.1.0]butane-1-carboxamide, 3n**

^{1}H NMR (500 MHz, Chloroform-*d*)

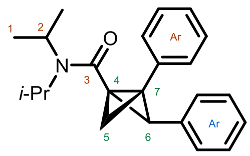

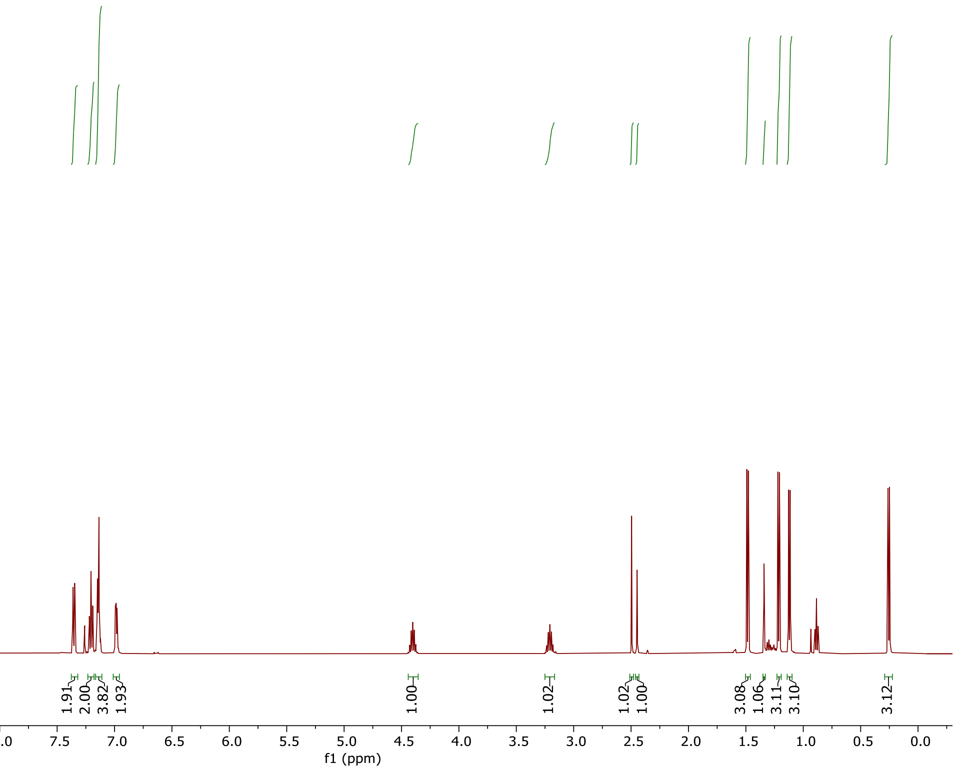

^{13}C NMR (101 MHz, Chloroform-*d*)

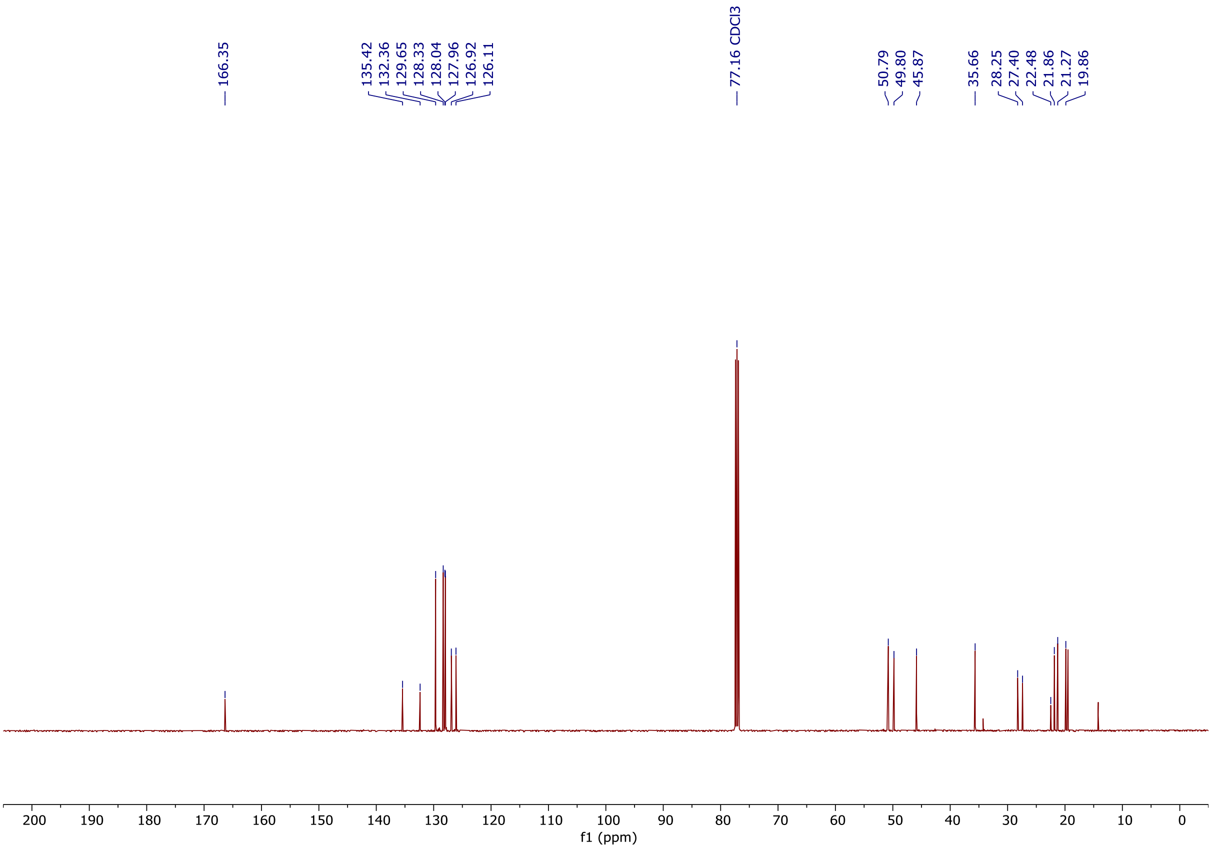

^{1}H COSY (500 MHz, Chloroform-*d*)

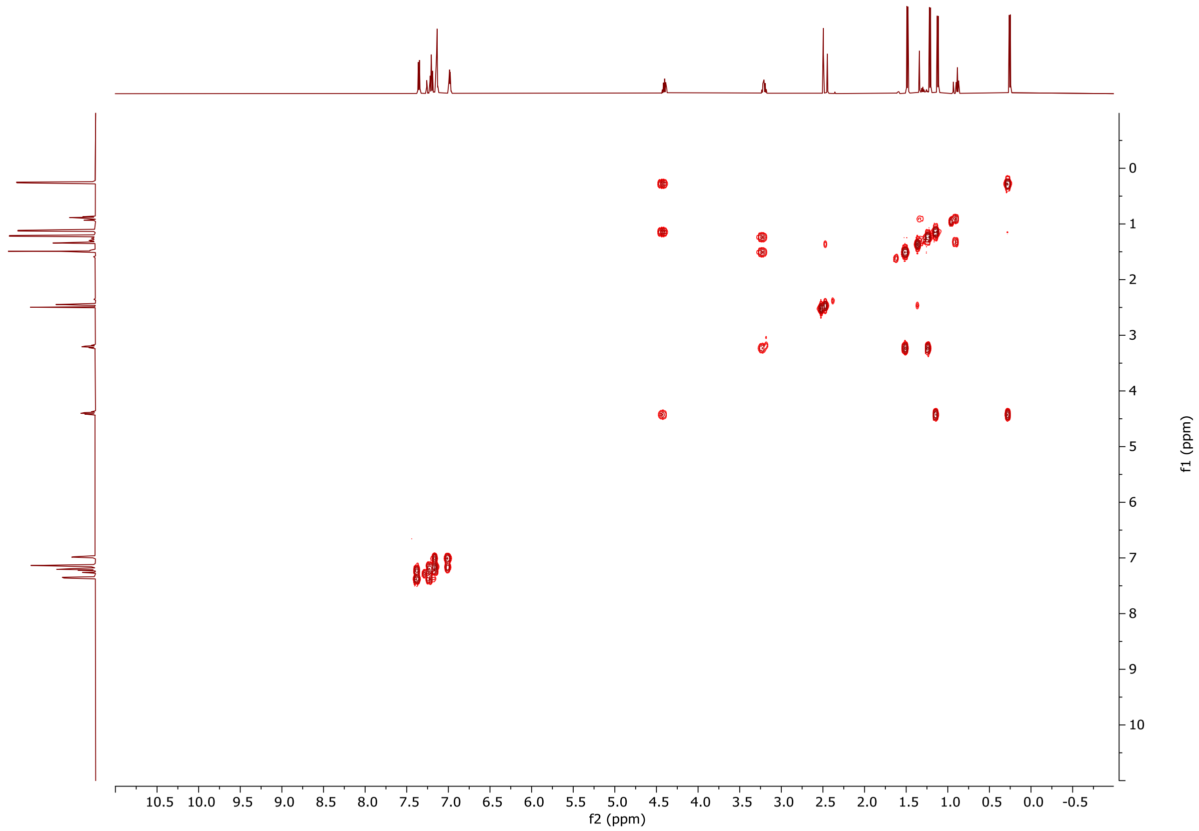

^{1}H/^{13}C HSQC (500/101 MHz, Chloroform-*d*)

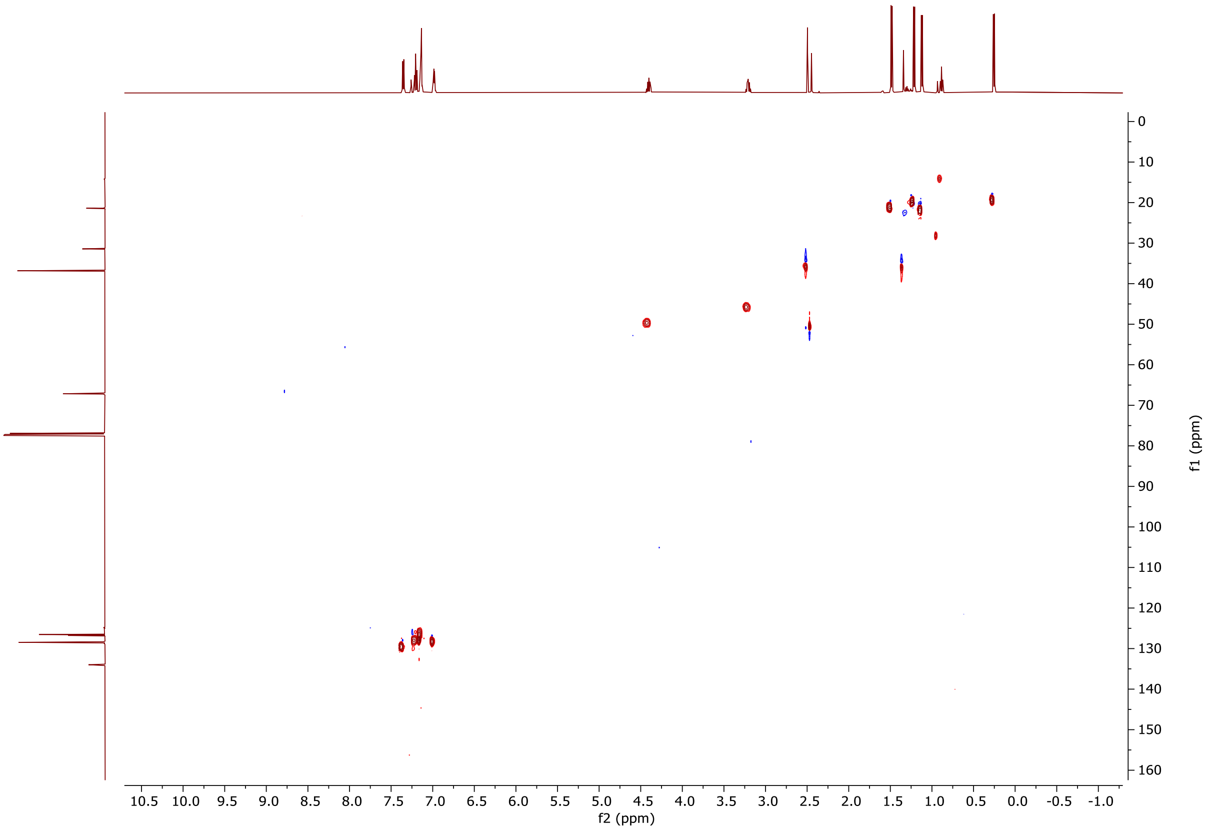

Methyl 4-((1*R,3*R**)-1-(diisopropylcarbamoyl)-3-(trimethylsilyl)bicyclo[1.1.0]butan-2-yl)benzoate, 3o**

^{1}H NMR (600 MHz, Chloroform-*d*)

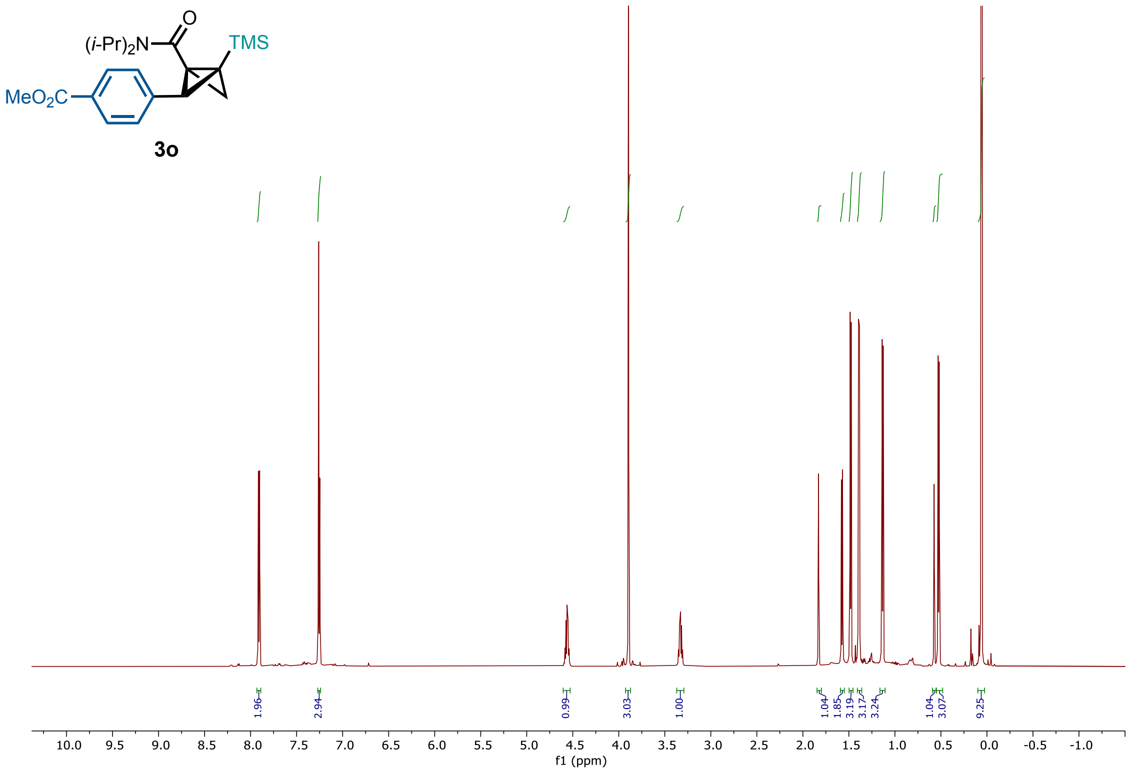

^{13}C NMR (151 MHz, Chloroform-*d*)

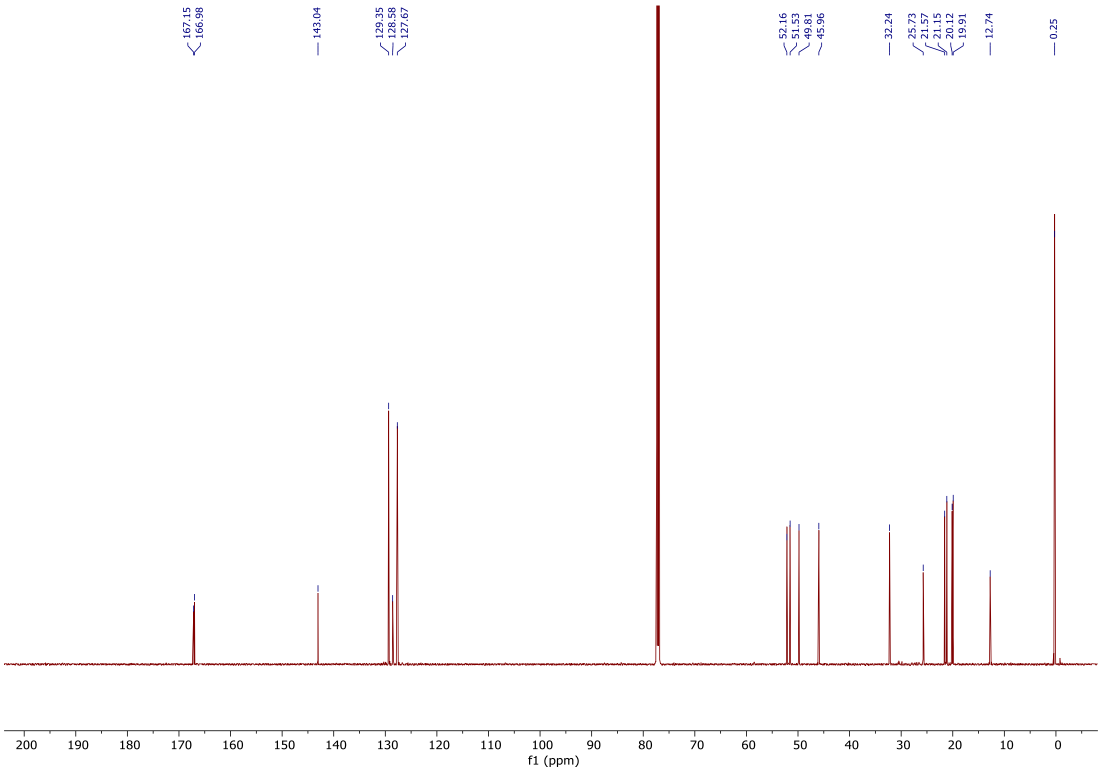
167.15
166.98
143.04
129.35
128.58
127.67
52.16
51.53
49.81
45.96
32.24
25.73
21.57
21.15
20.12
19.91
12.74
0.25
200 190 180 170 160 150 140 130 120 110 100 90 80 70 60 50 40 30 20 10 0
f1 (ppm)

^{1}**H COSY** (600 MHz, Chloroform-*d*)

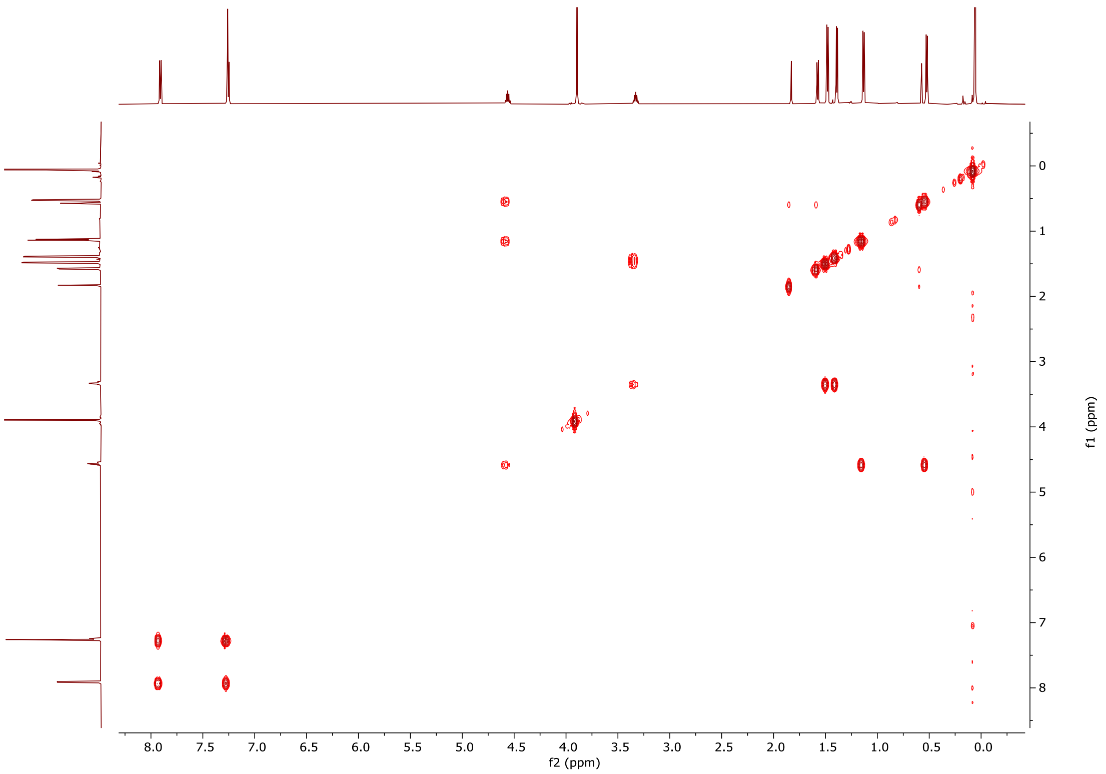

^{1}**H/**^{13}**C HSQC** (600/151 MHz, Chloroform-*d*)

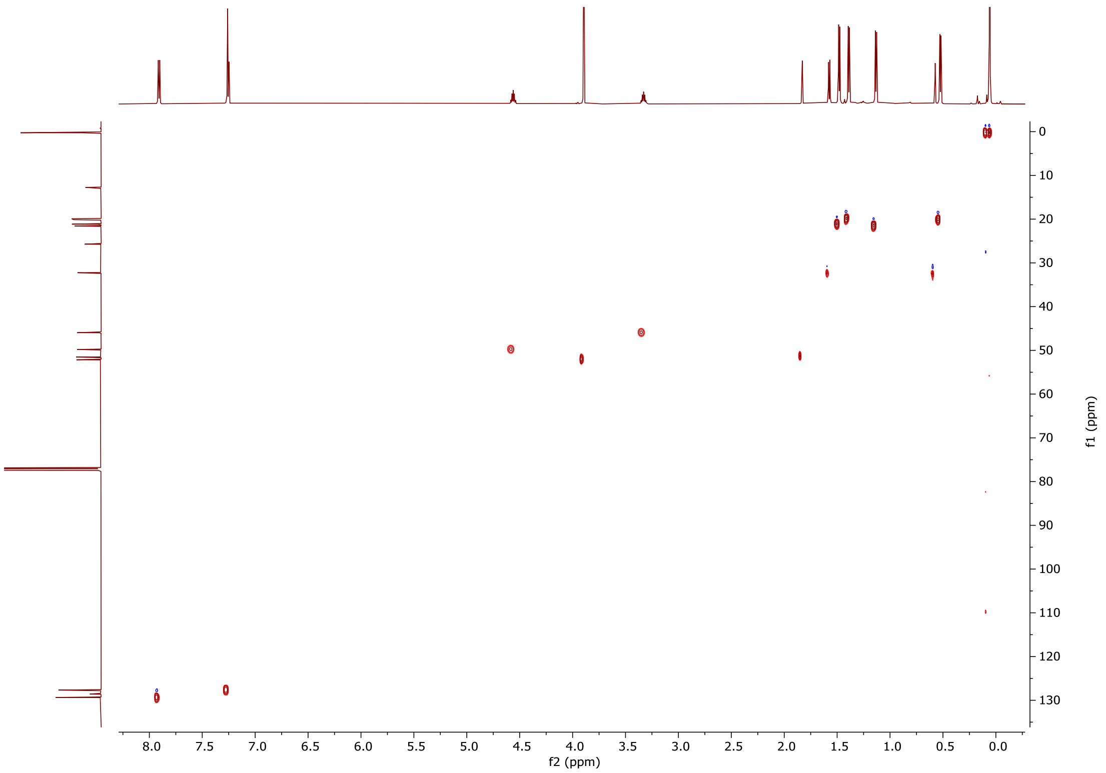

$^{1}H/^{13}C$ **HMBC** (600/151 MHz, Chloroform-*d*)

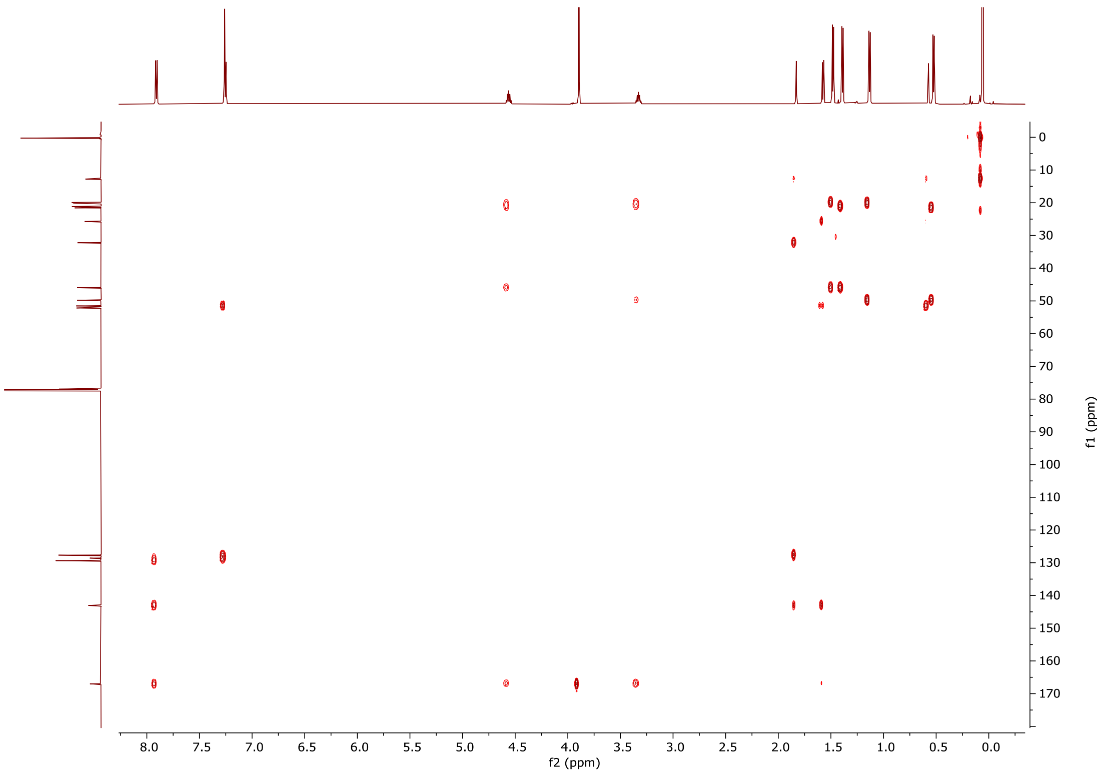

^{1}H **NOSEY** (600 MHz, Chloroform-*d*)

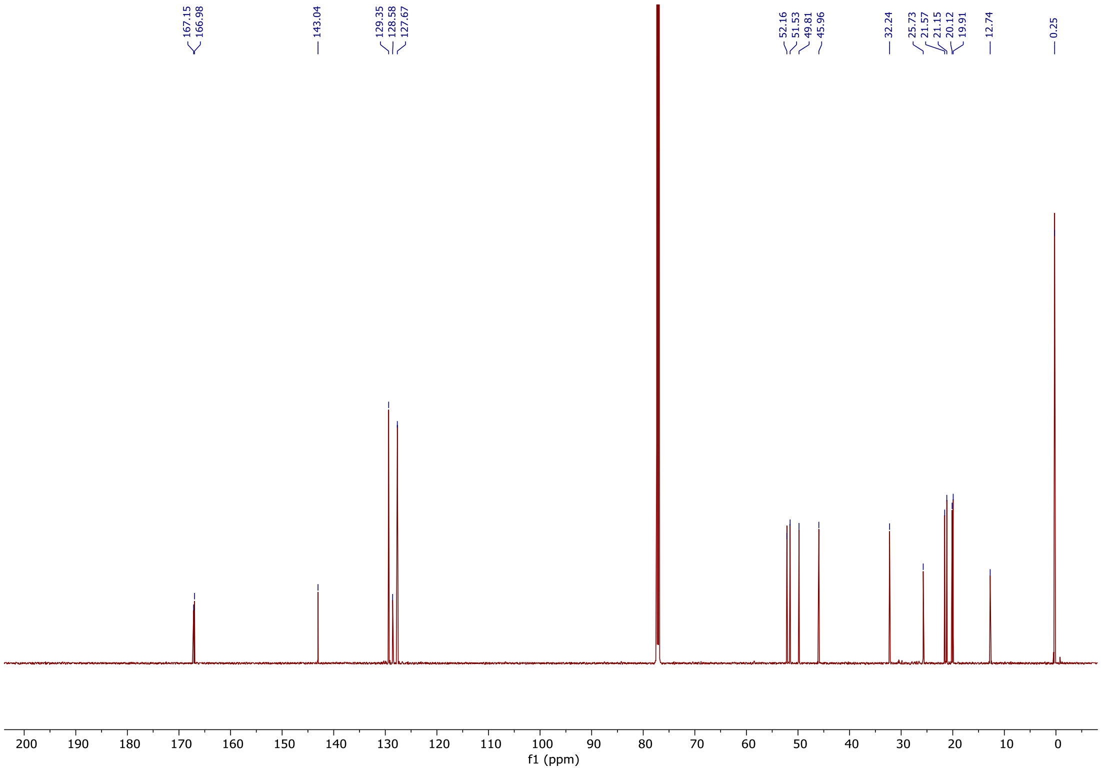

Methyl 4-((1*S,3*S**,4*R**)-1-(diisopropylcarbamoyl)-3,4-dimethylbicyclo[1.1.0]butan-2-yl)benzoate, 3p**

^{1}H NMR (600 MHz, Chloroform-*d*)

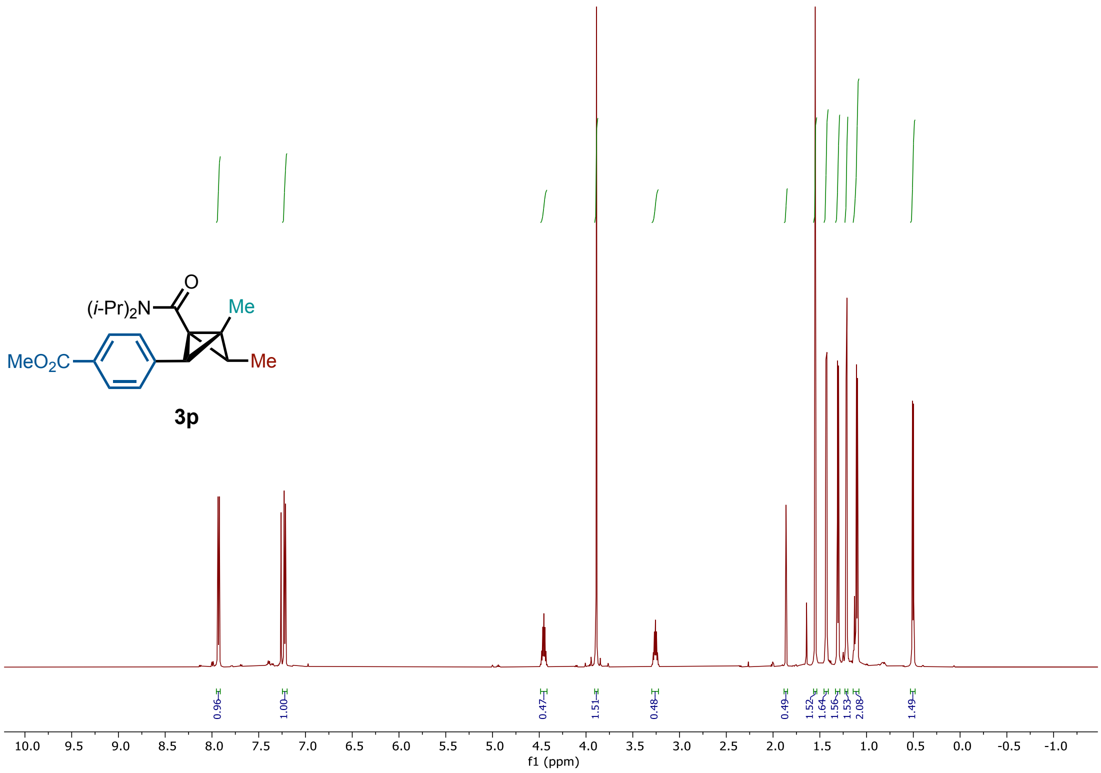

^{13}C NMR (151 MHz, Chloroform-*d*)

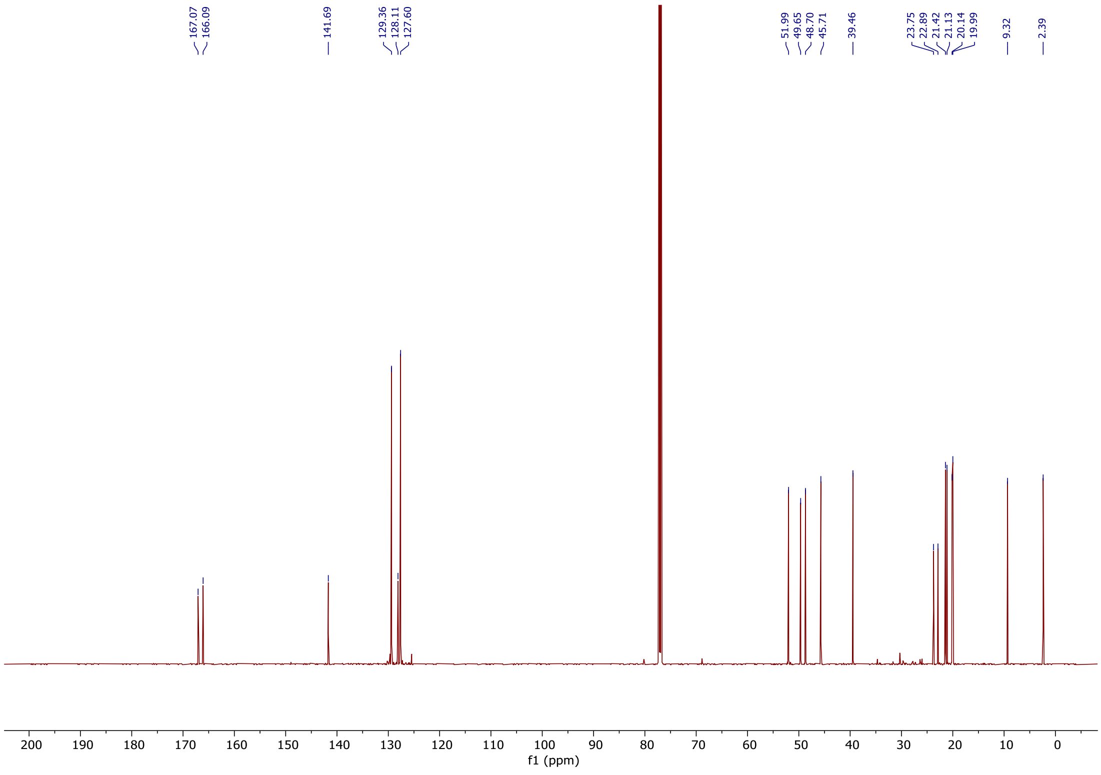

¹H COSY (600 MHz, Chloroform-*d*)

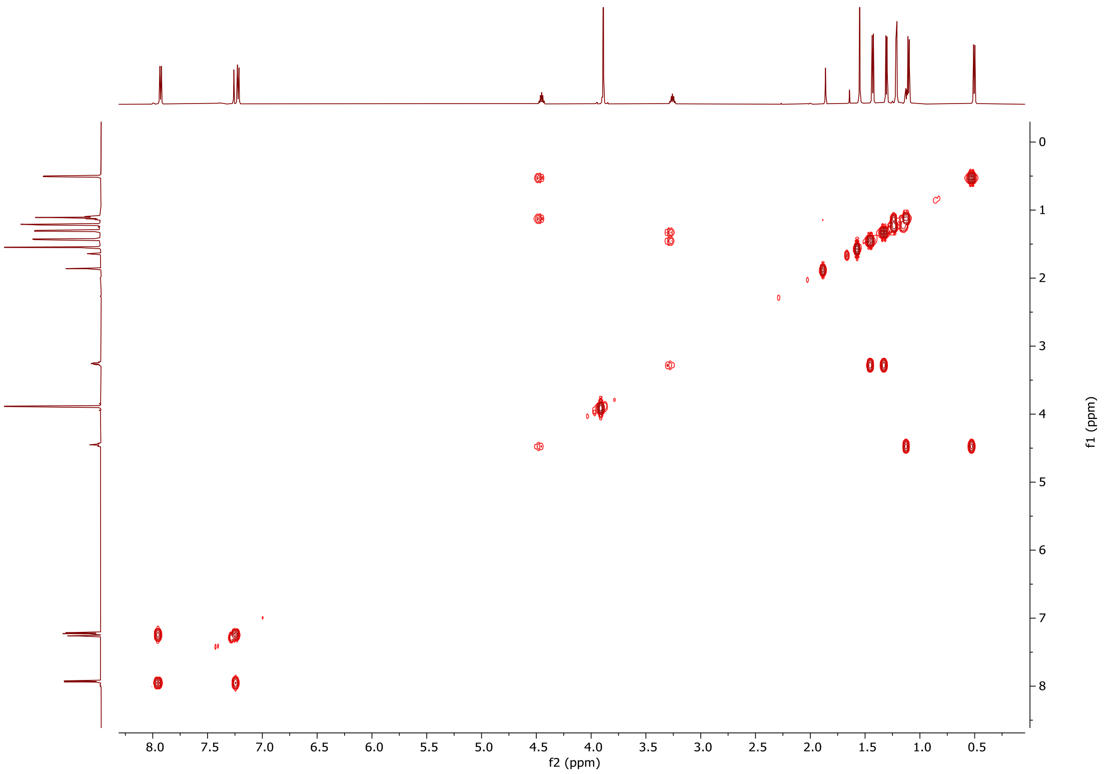

¹H/¹³C HSQC (600/151 MHz, Chloroform-*d*)

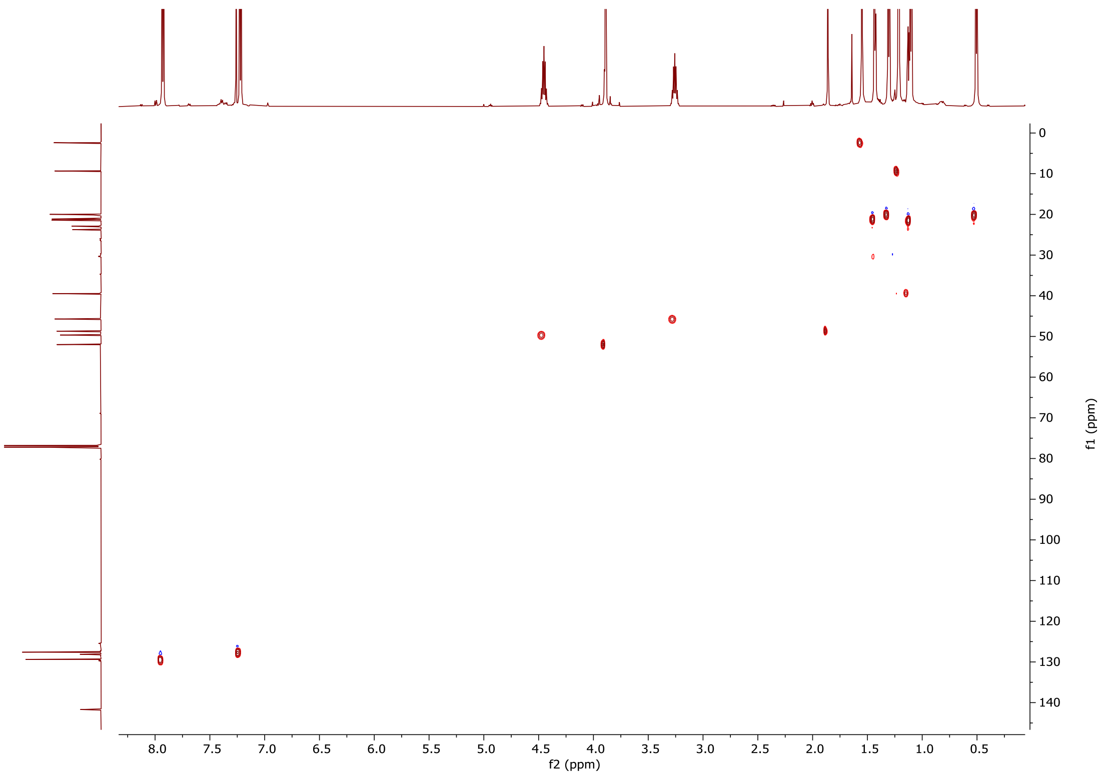

^{1}H/^{13}C HMBC (600/151 MHz, Chloroform-*d*)

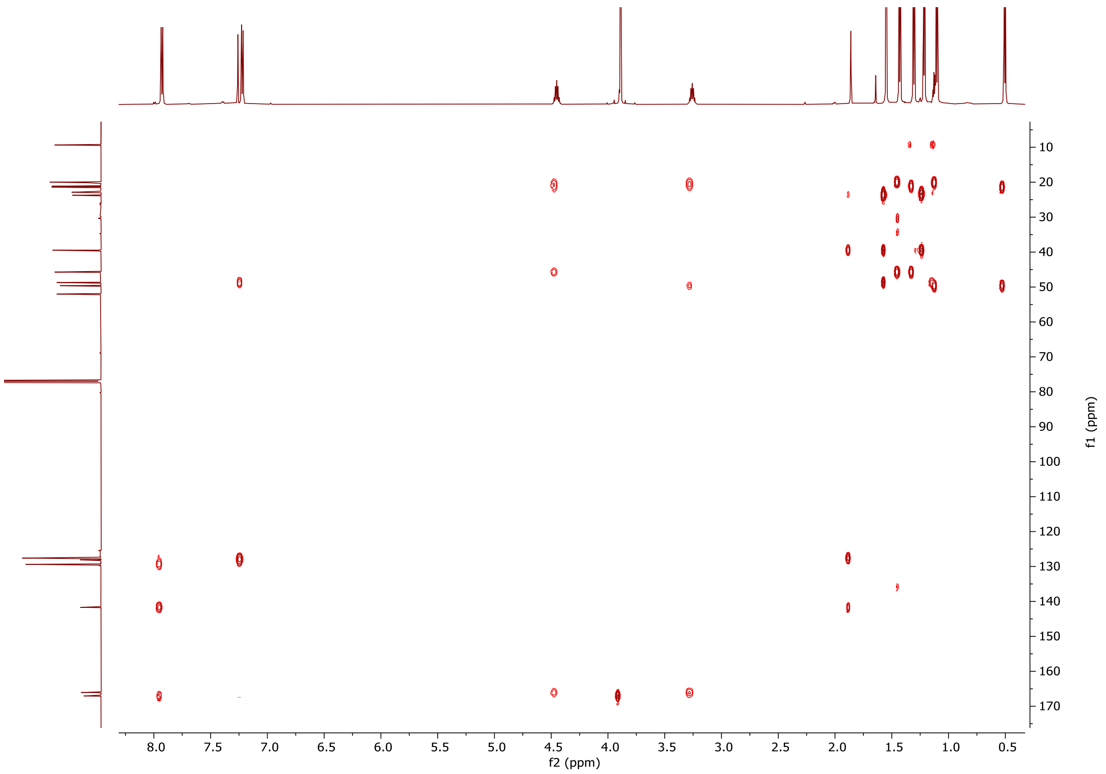

^{1}H NOSEY (600 MHz, Chloroform-*d*)

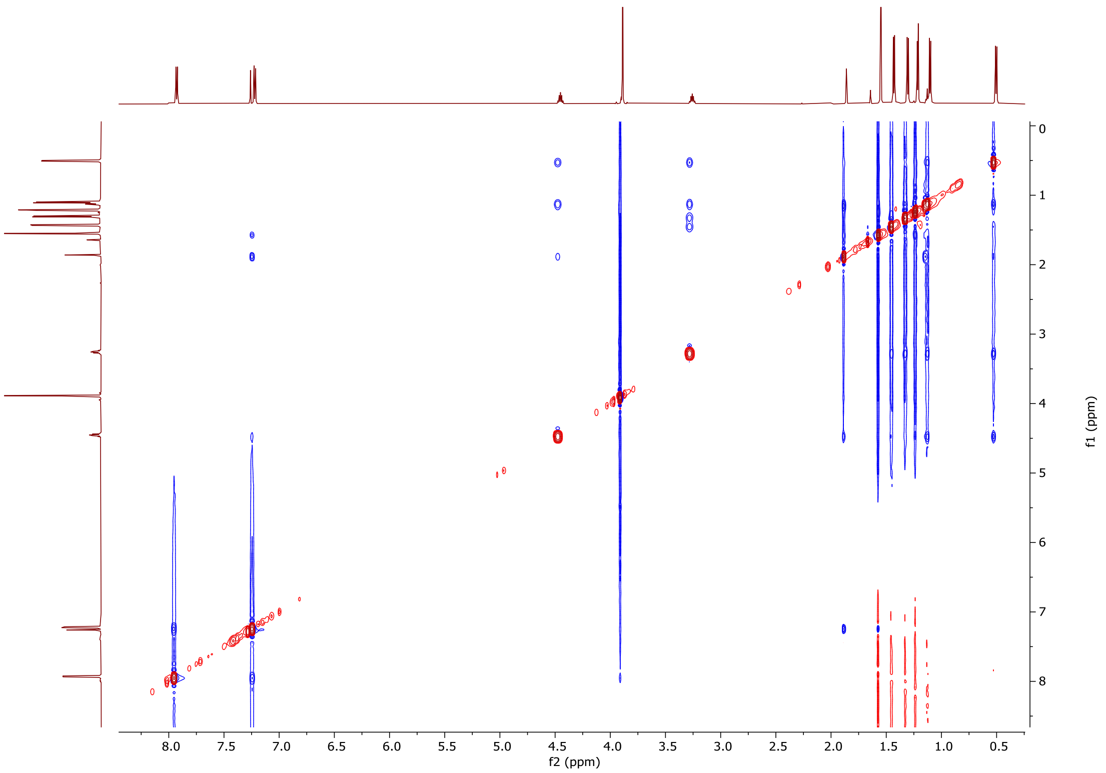

Methyl 4-((1*S,3*S**)-1-(*tert*-butylsulfonyl)-3-methylbicyclo[1.1.0]butan-2-yl)benzoate, 4a**

^{1}H NMR (600 MHz, Chloroform-*d*)

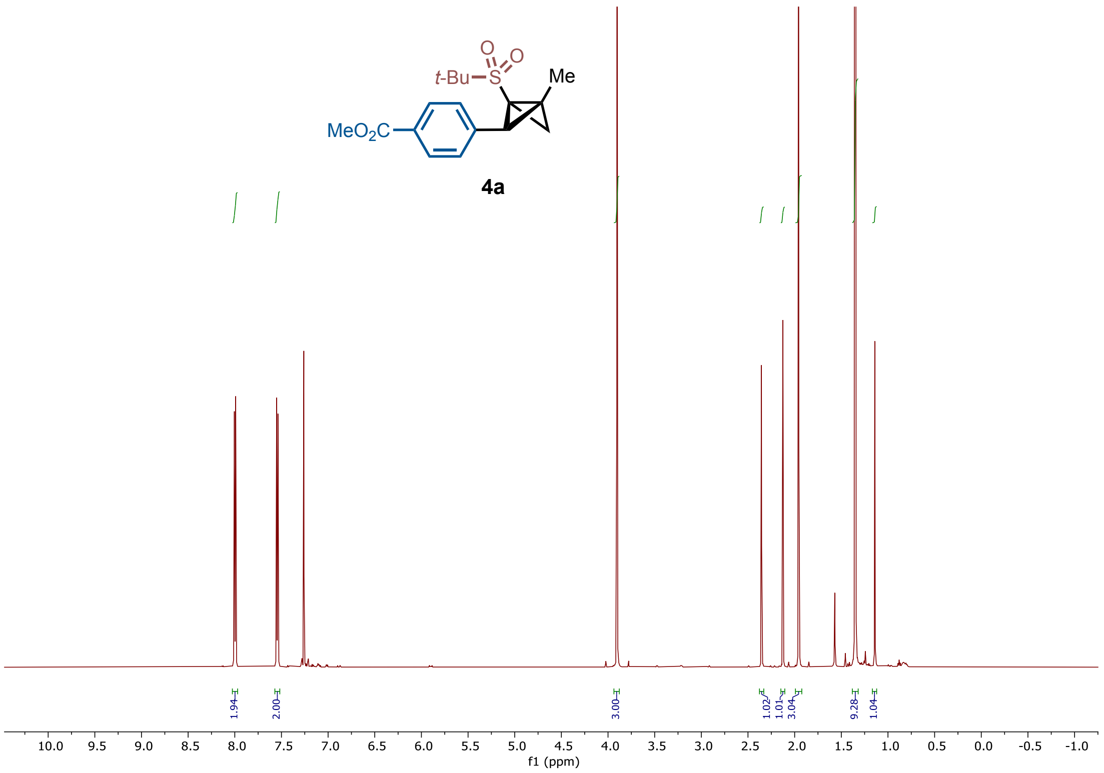

^{13}C NMR (151 MHz, Chloroform-*d*)

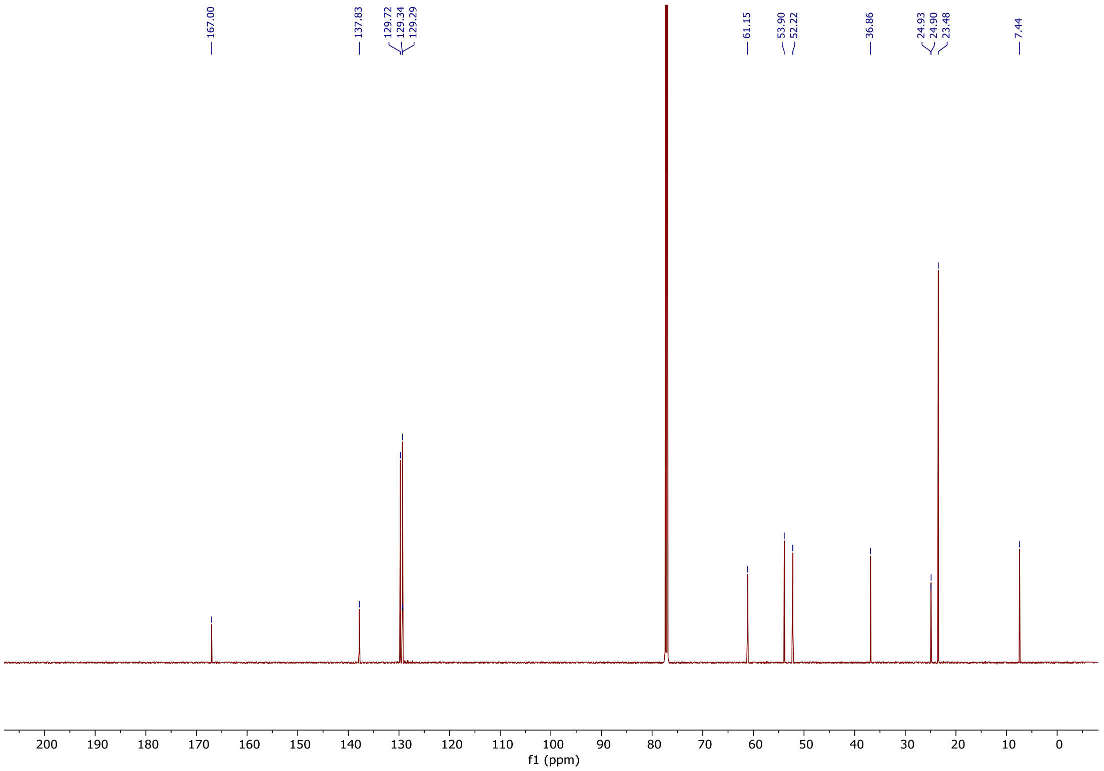

^{1}H COSY (600 MHz, Chloroform-*d*)

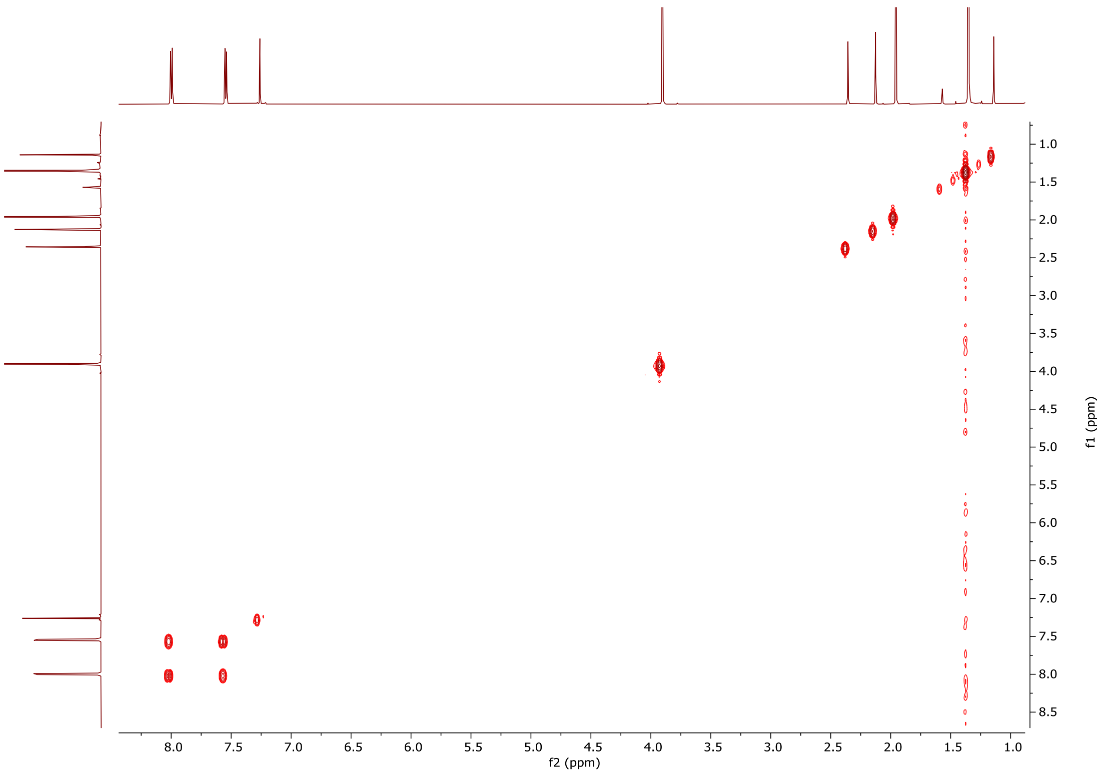

$^{1}H/^{13}C$ HSQC (600/151 MHz, Chloroform-*d*)

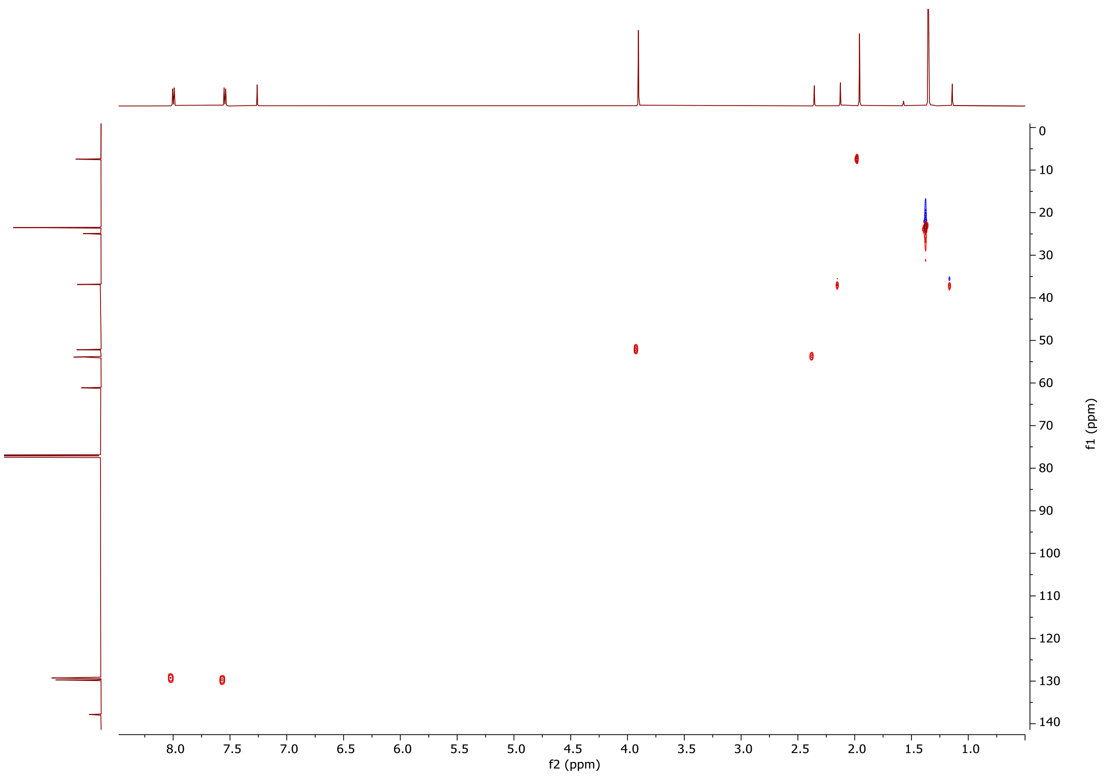

^{1}H/^{13}C HMBC (600/151 MHz, Chloroform-*d*)

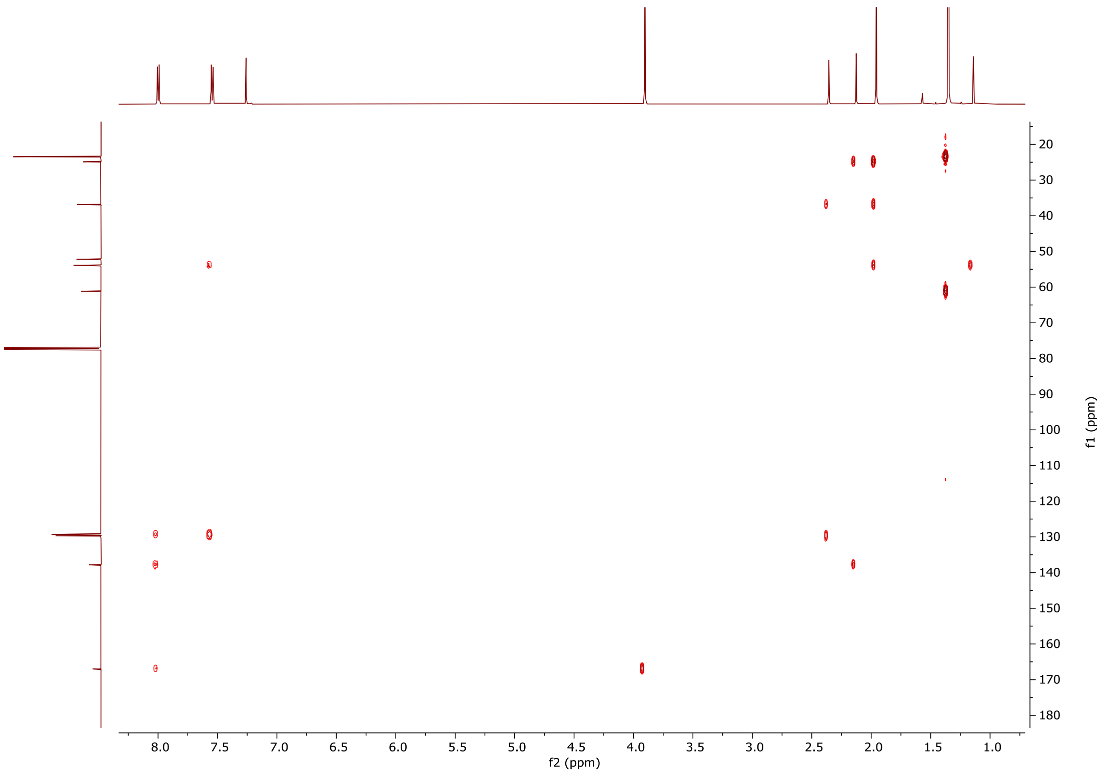

(1*S,2*S**)-1-allyl-*N*,*N*-diisopropyl-3-methylene-2-(4-(trifluoromethyl)phenyl)cyclobutane-1-carboxamide, 6**

^{1}H NMR (600 MHz, Chloroform-*d*)

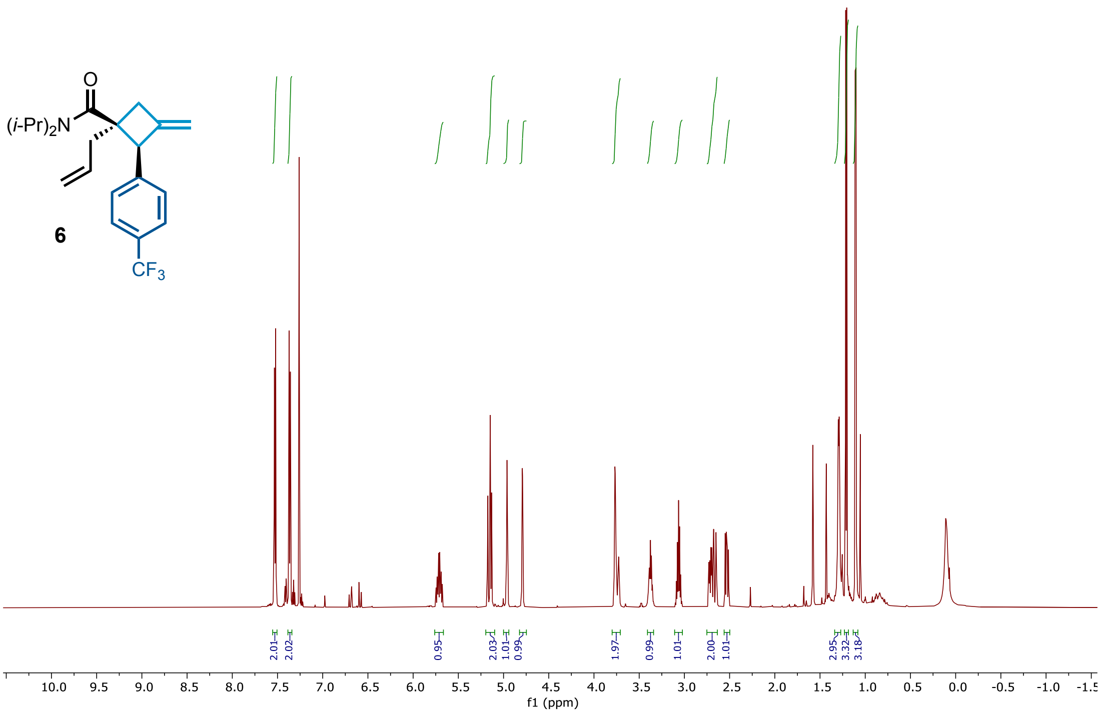

^{13}C NMR (151 MHz, Chloroform-*d*)

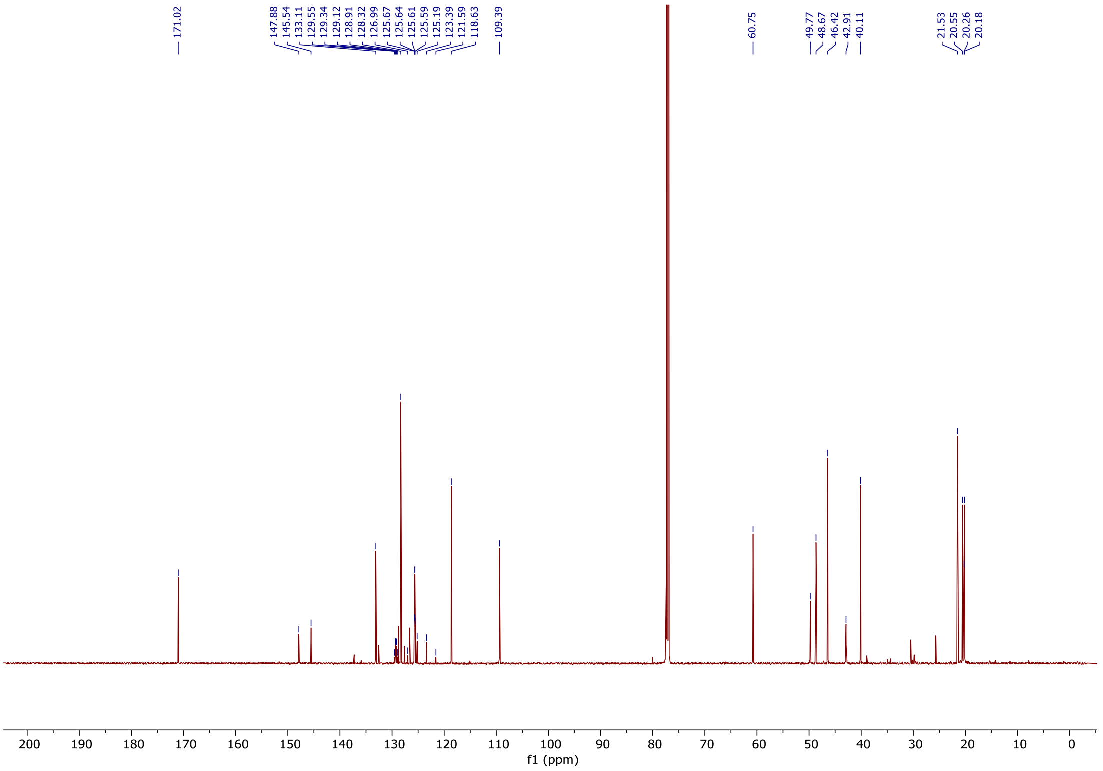

^{1}H COSY (600 MHz, Chloroform-*d*)

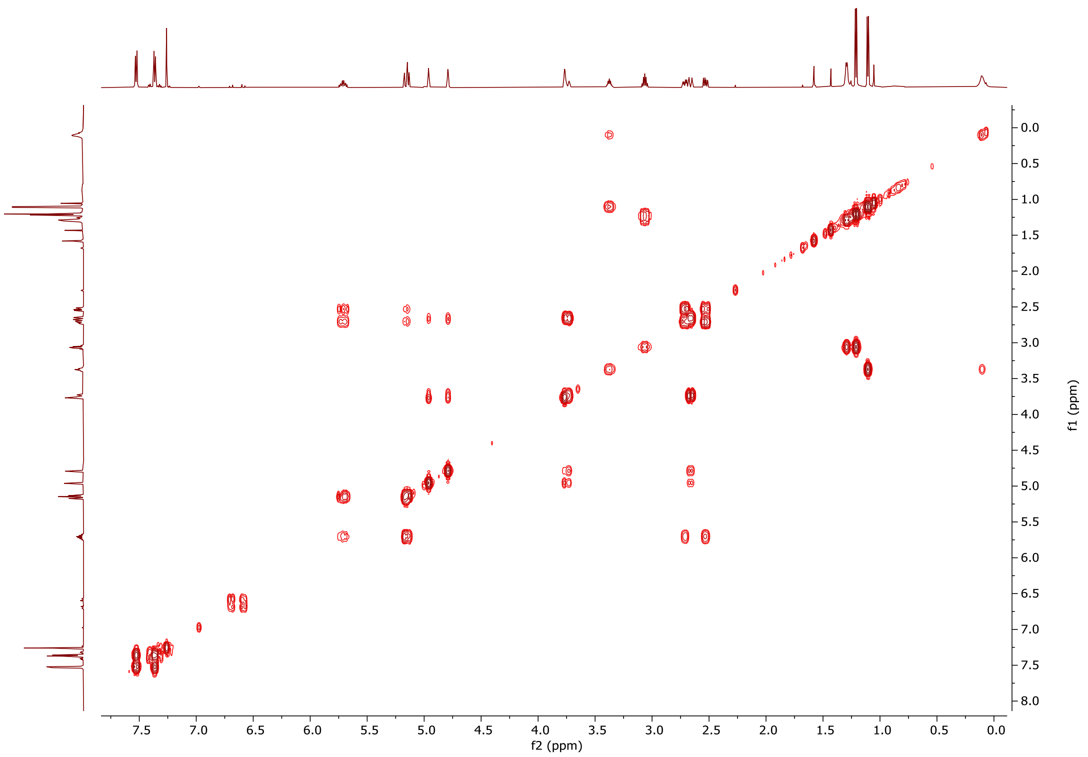

^{1}H/^{13}C HSQC (600/151 MHz, Chloroform-*d*)

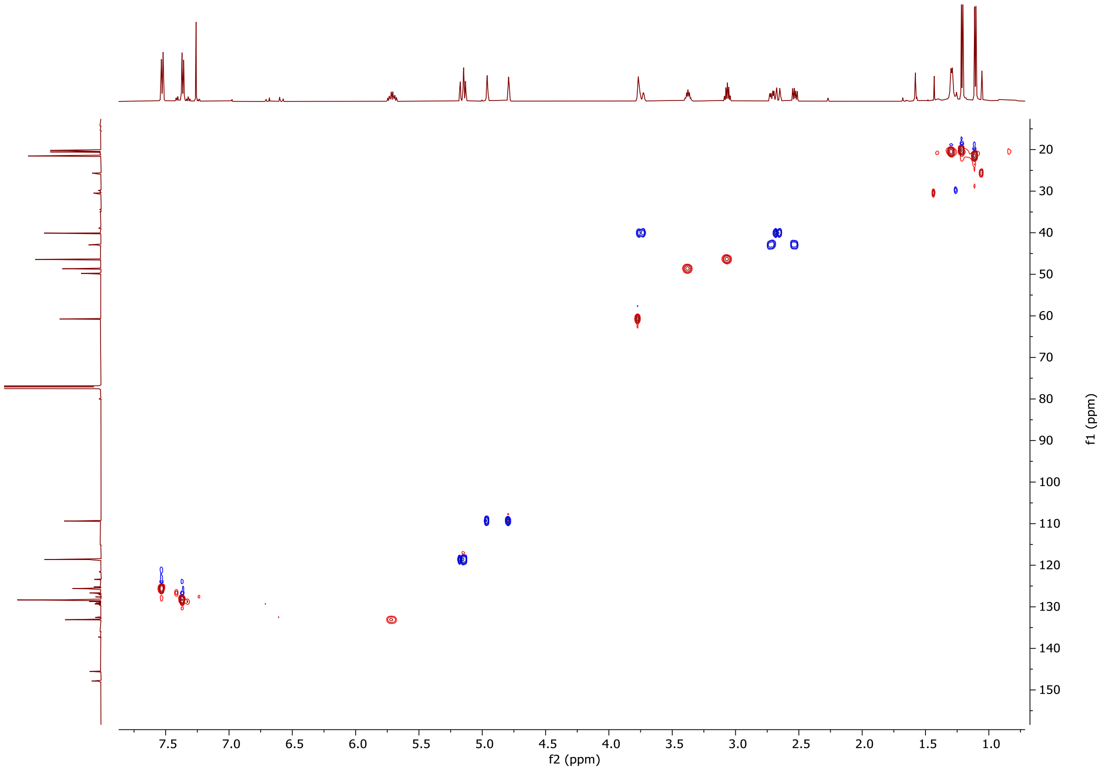

$^{1}H/^{13}C$ **HMBC** (600/151 MHz, Chloroform-*d*)

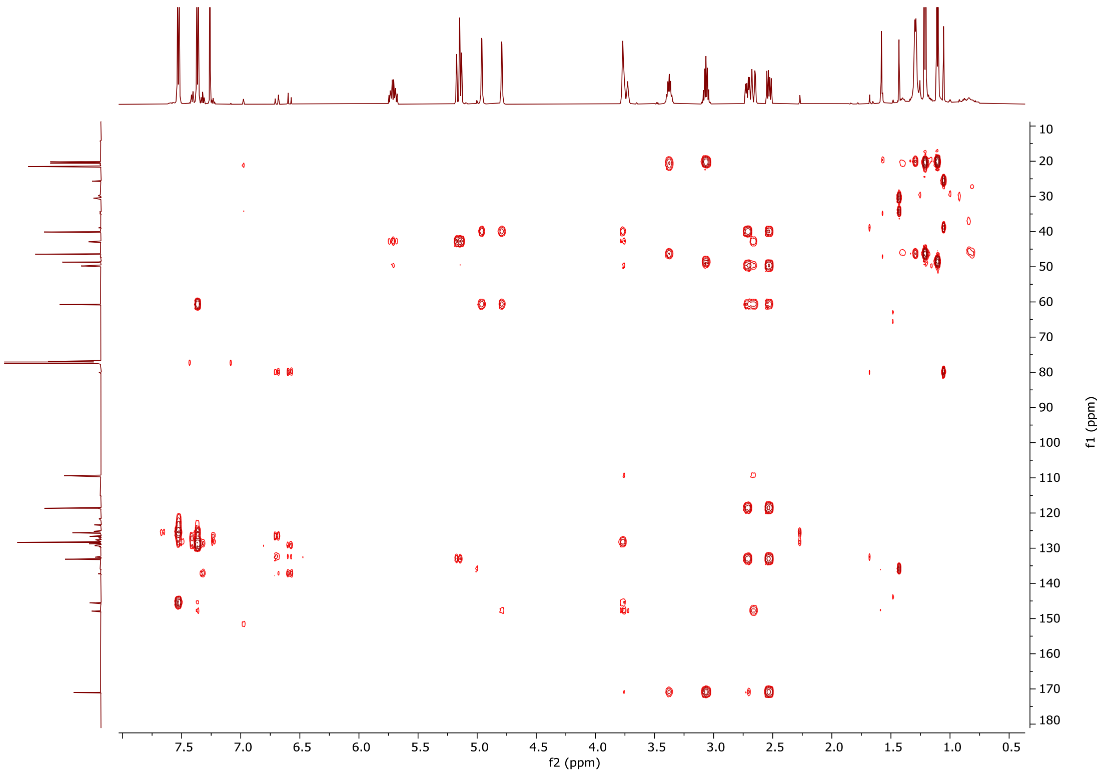

^{19}F **NMR** (471 MHz, Chloroform-*d*)

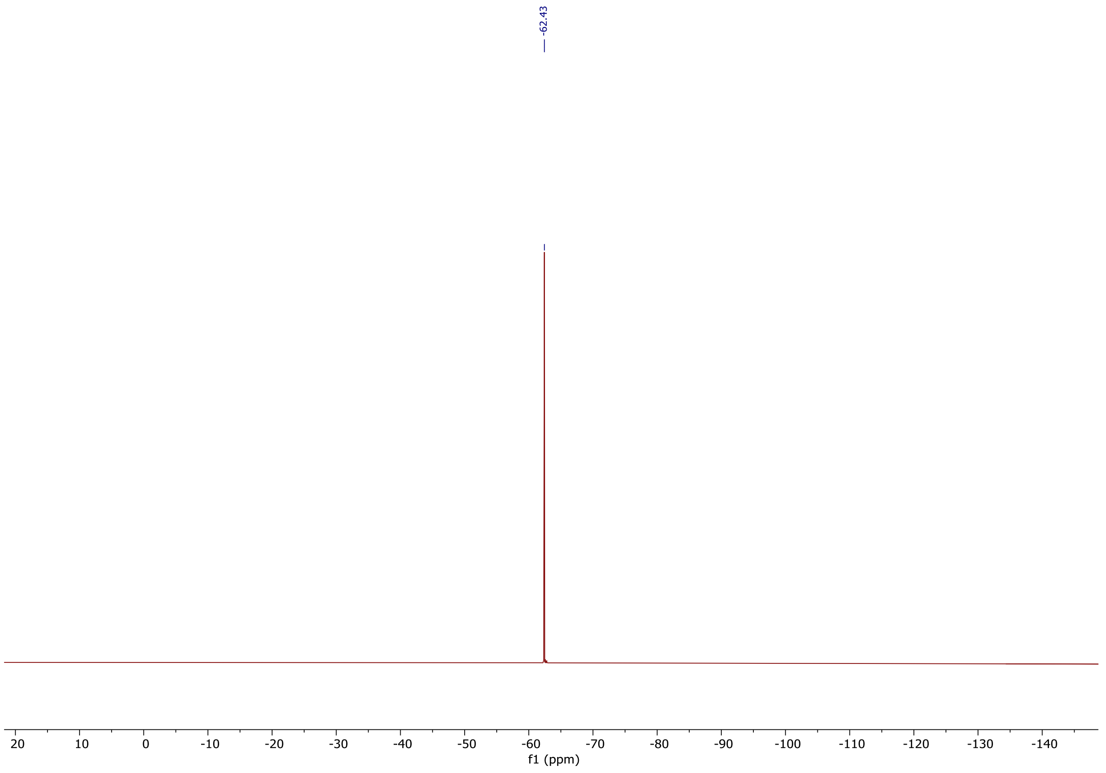

¹H NOSEY (600 MHz, Chloroform-*d*)

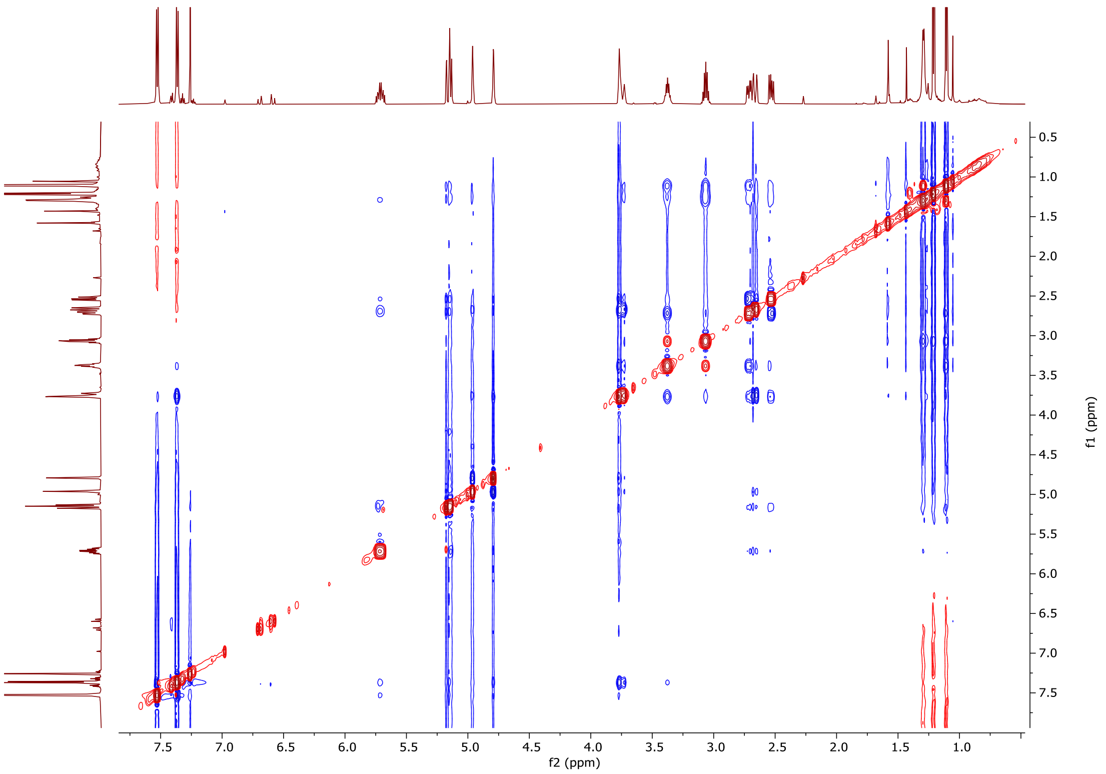

4. References

1. L. Palatinus and G. Chapuis, *J. Appl. Cryst.*, 2007, **40**, 786-790.
2. P. W. Betteridge, J. R. Carruthers, R. I. Cooper, K. Prout and D. J. Watkin, *J. Appl. Cryst.*, 2003, **36**, 1487.
3. R. I. Cooper, A. L. Thompson and D. J. Watkin, *J. Appl. Cryst.*, 2010, **43**, 1100-1107.
